# Jasminumosides F–K, oligomeric secoiridoid glycosides from jasmin: the flowers of *Jasminum sambac* (L.) Aiton

**DOI:** 10.1007/s11418-026-02018-5

**Published:** 2026-02-24

**Authors:** Naoki Inoue, Yoshiaki Manse, Toshio Morikawa

**Affiliations:** 1 Pharmaceutical Research and Technology Institute, Kindai University, 3-4-1 Kowakae, Higashi-osaka, Osaka, 577-8502 Japan; 2 Antiaging Center, Kindai University, 3-4-1 Kowakae, Higashi-osaka, Osaka, 577-8502 Japan; 3 Present Address: School of Pharmacy and Pharmaceutical Sciences, Mukogawa Women’s University, 11–68 Koshien Kyuban-cho, Nishinomiya, 663–9942 Hyogo Japan

**Keywords:** Jasmine, *Jasminum sambac*, Jasminumoside, Oligomeric secoiridoid glycoside, Oleaceae, MONOTORI

## Abstract

Six new oligomeric secoiridoid glycosides, namely jasminumosides F–K (**1**–**6**), were isolated from the methanolic extract of jasmine, the flower of *Jasminum sambac* (L.) Aiton (Oleaceae). Their structures were determined based on their spectroscopic properties and chemical evidence.

**Graphical abstract:**

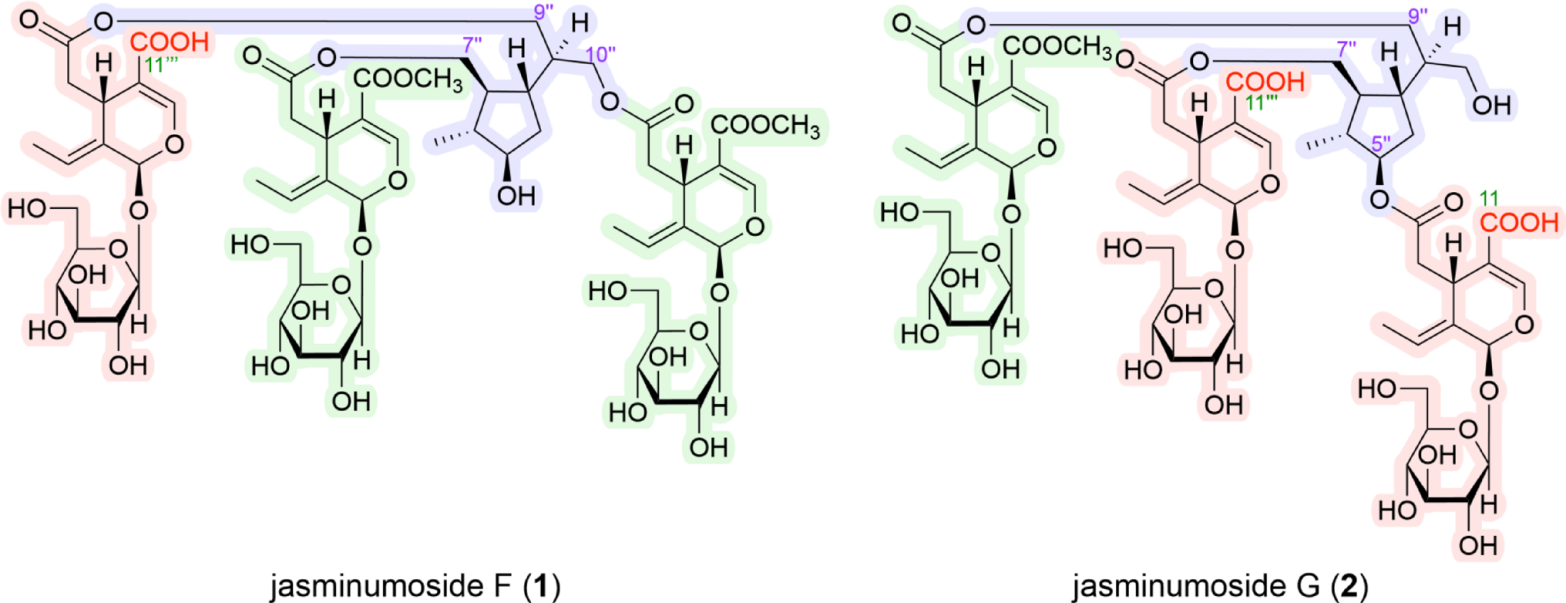

**Supplementary Information:**

The online version contains supplementary material available at 10.1007/s11418-026-02018-5.

## Introduction

Iridoids are naturally occurring monoterpenoid compounds characterized by a 1-isopropyl-2,3-dimethylcyclopentane skeleton. Most of them possess a six-membered ring containing an oxygen atom fused to a cyclopentane ring. In the plant kingdom, iridoids derive from 9-hydroxynerol via phosphorylation, followed by additional steps including cyclization, oxidation, and glycosidation. Secoiridoids, a subclass of iridoids, are formed via oxidative cleavage of the C7-C8 bond in the cyclopentane ring. Iridoids and secoiridoids are often found in medicinal plants as glycosides and their oligomers [1–7]. A variety of biological and pharmacological activities have been reported for iridoids and secoiridoids including antibacterial, antifungal, antileishmanial, anticancer, antidiabetic, antihyperlipidemic, anti-osteoporosis, antioxidant, anti-protozoal, hepatoprotective, immunomodulatory, neuroprotective, and neuritogenic activities [1, 3, 4, 7–14]. Our previous studies on the chemical constituents of medicinal plants such as *Cistanche tubulosa* (Schrenk) R. Wight [15–17], *Picrorhiza kurroa* Royle ex Benth [18–20]., and *Jasminum sambac* (L.) Aiton [21] have led to the isolation of several iridoids and secoiridoids. In this study, we further pursued the separation of the constituents of *J. sambac* flowers and isolated six new oligomeric secoiridoid glycosides, named jasminumosides F–K (**1**–**6**), from a methanol extract. This work describes the isolation and structural elucidation of **1**–**6**.

## Results and discussion

### Isolation of Jasminumosides F–K (**1**–**6**)

**Fig. 1:**
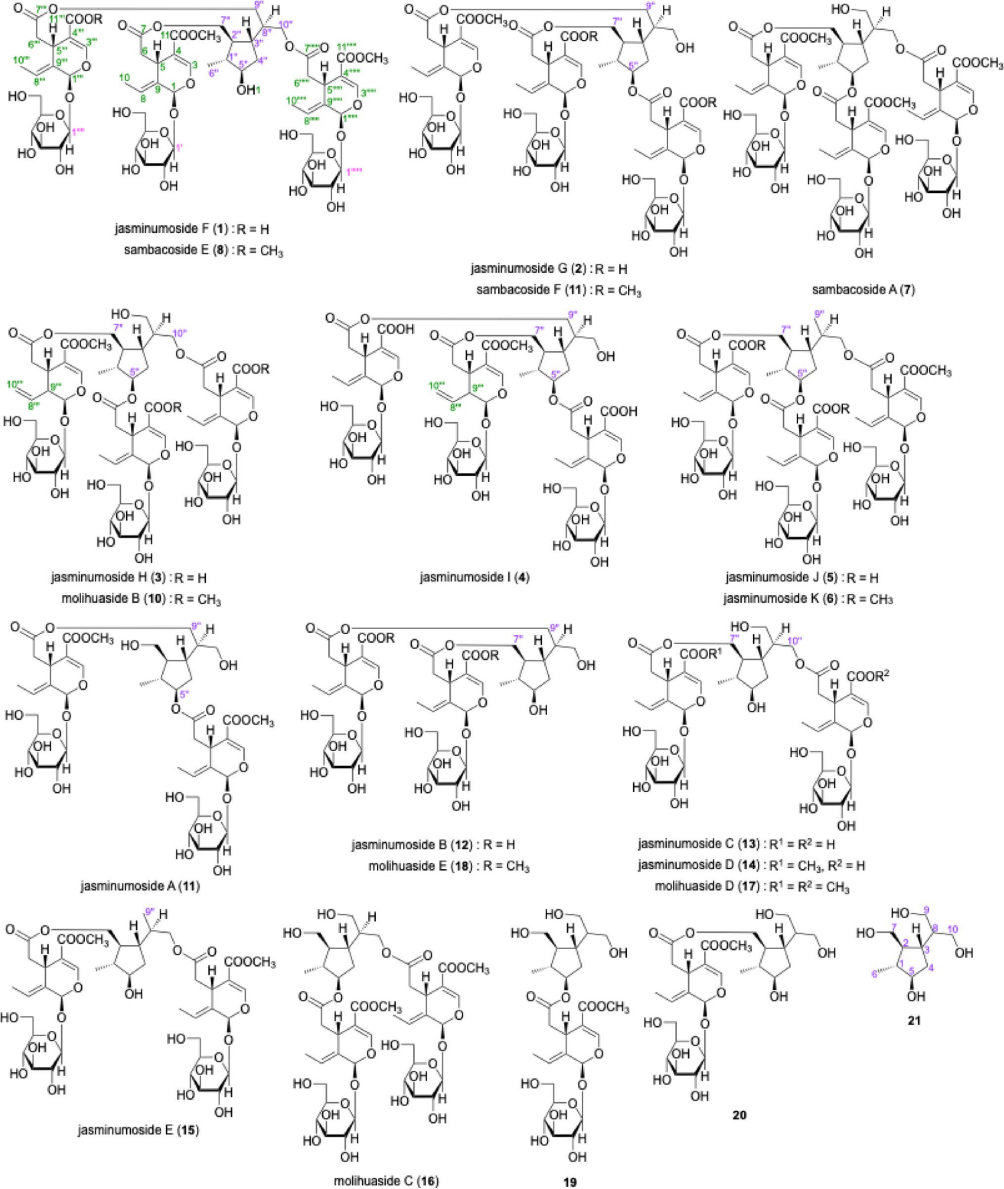
Structures of iridoid constituents including jasminamosides F–K (**1**–**6**) obtained from the flowers of *J. sambac*

The Oleaceae plant *J. sambac*, popularly known as jasmine, originates from southern and southeastern Asia. The flowers of this plant are commonly used in the preparation of an essential oil and to make the beverage jasmine tea, as well as in folk medicine for the treatment of diarrhea, abdominal pain, conjunctivitis, asthma, cancer, toothache, dermatitis, and wound healing [22]. We previously reported the isolation of 14 secoiridoids (**7**–**20**), including five new oligomeric secoiridoid glycosides, jasminumosides A–E (**11**–**15**), and a monoterpene (**21**), from the methanol extract of the flowers of *J. sambac*. In addition, the extract and the principal oligomeric secoiridoid glycosides sambacoside A (**7**) and molihuasides C (**16**) and D (**17**) were found to exhibit hepatoprotective activity against d-galactosamine/lipopolysaccharide-induced liver injury in a mouse model [21]. In the present study, we isolated six new oligomeric secoiridoid glycosides, including jasminumosides F (**1**, 0.0106%, from plant material), G (**2**, 0.0249%), H (**3**, 0.0209%), I (**4**, 0.0039%), J (**5**, 0.0083%), and K (**6**, 0.0039%), using normal-phase silica gel and reversed-phase ODS column chromatography, followed by preparative HPLC, as shown in Fig. 1.

### Structure determination

#### Jasminumoside F (**1**)

**Fig. 2:**
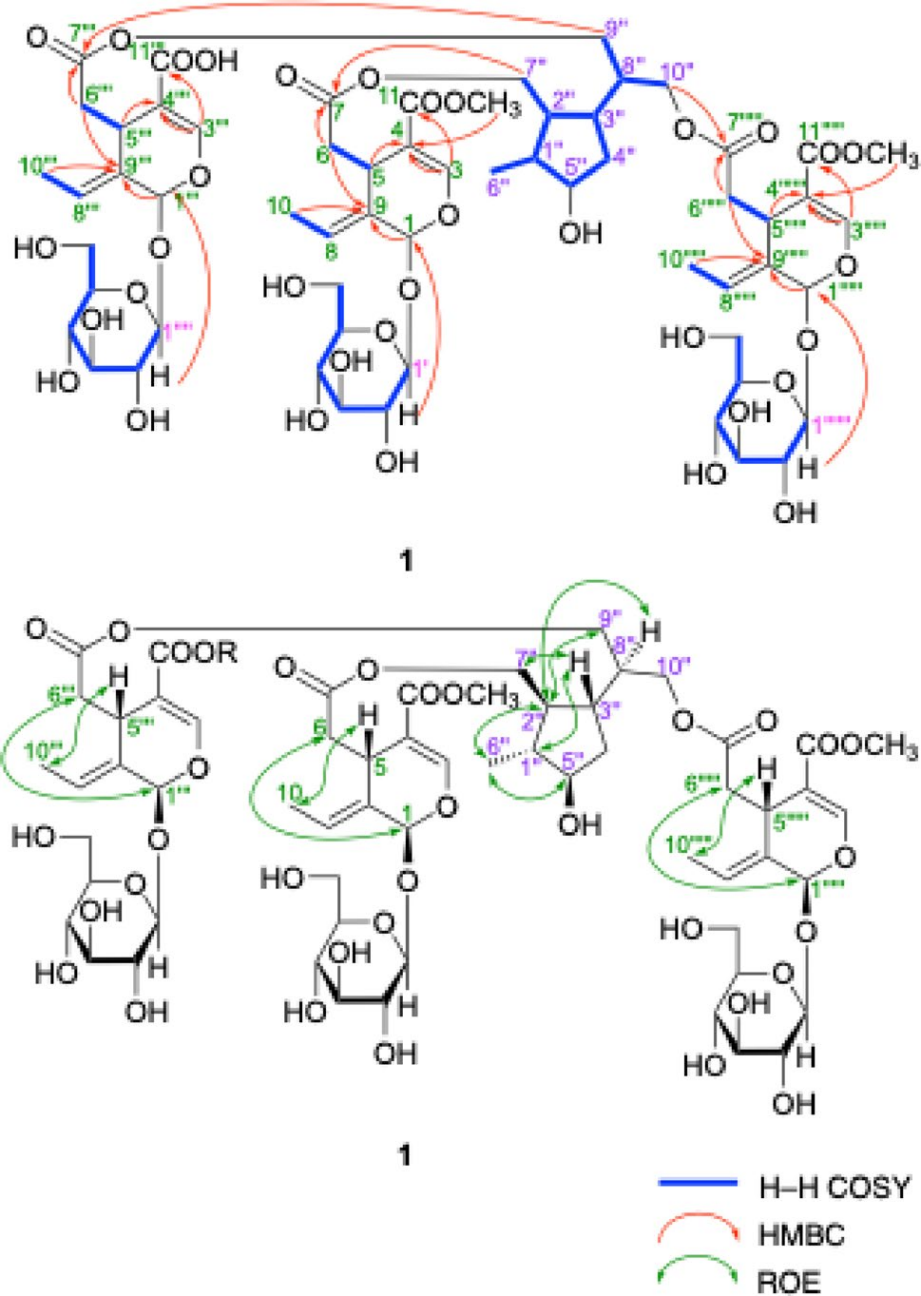
^1^H–^1^H COSY, HMBC, and pROESY correlations of **1**

Jasminumoside F (**1**) was obtained as a white powder with a negative optical rotation ([*α*]_D_^25^ − 156.0 in MeOH). In the UV spectrum of **1**, an absorption maximum was observed at 235 nm (log *ε* 4.46), while the IR spectrum showed absorption bands at 1717 and 1636 cm^–1^, indicating the presence of the chromophore –OC(= O)–C = CH–O– moiety [23–25], as well as broad bands at 3393 and 1076 cm^–1^ indicative of a glycoside structure. According to positive-ion ESIMS, a quasi-molecular ion peak was observed at *m/z* 1371 [M + Na]^+^, while HRESIMS analysis indicated a molecular formula of C_60_H_84_O_34_. Further treatment of **1** with 1 M HCl liberated d-glucose, which was identified by HPLC using an optical rotation detector [21, 26, 27]. The ^1^H and ^13^C NMR spectra (CD_3_OD, Table 1), which were assigned with the aid of distortionless enhancement by polarization transfer (DEPT), ^1^H-^1^H homonuclear correlation (COSY), heteronuclear multiple quantum coherence (HMQC), and heteronuclear multiple-bond correlation (HMBC) experiments (Fig. 2), exhibited triple signals for the protons of the above-mentioned chromophore [*δ *7.49, 7.516, 7.521 (1H each, all s, H-3, 3’’’, 3’’’’’)], methylenes [*δ *2.48 (1H, dd, *J* = 8.9, 14.4 Hz), 2.49 (2H, dd, *J* = 8.9, 14.4 Hz), 2.72 (1H, dd, *J* = 4.6, 14.4 Hz), 2.73 (1H, dd, *J* = 4.6, 14.4 Hz), 2.76 (1H, dd, *J* = 4.6, 14.4 Hz), H_2_-6, 6’’’, 6’’’’’], methines [*δ *3.99, 3.99, 3.99 (1H each, all m, H-5, 5’’’, 5’’’’’)], ethylidene groups [*δ *1.74, 1.74, 1.74 (3H each, all d, *J* = 6.9 Hz, H_3_-10, 10’’’, 10’’’’’), 6.10, 6.10, 6.10 (1H each, all q, *J* = 7.1 Hz, H-8, 8’’’, 8’’’’’)], acetals [*δ *5.92, 5.92, 5.93 (1H each, all br s, H-1, 1’’’, 1’’’’’)], and *β*-glucopyranosyl parts [*δ *4.81, 4.81, 4.81 (1H each, all d, *J* = 7.8 Hz, H-1’, 1’’’’, 1’’’’’’)] along with signals due to a methyl [*δ *1.04 (3H, d, *J* = 6.6 Hz, H_3_-6’’)], methylene, and three methylene groups bearing an oxygen function {*δ *[1.70 (1H, m), 1.84 (1H, ddd, *J* = 6.4, 6.6, 12.0 Hz), H_2_-4’’], [4.01 (1H, dd, *J* = 5.0, 11.2 Hz), 4.13 (1H, dd, *J* = 6.2, 11.2 Hz), H_2_-7’’], [4.03 (1H, dd, *J* = 6.2, 11.2 Hz), 4.16 (1H, dd, *J* = 5.0, 11.2 Hz), H_2_-10’’], 4.09 (2H, dd, *J* = 6.0, 11.2 Hz, H_2_-9’’)}, four methine groups and a methine bearing an oxygen function [*δ *1.65, 1.67, 2.07, 2.09 (1H each, all m, H-1’’, 2’’, 8’’, 3’’), 3.69 (1H, m, H-5’’)]. Additionally, two carbomethoxy signals [*δ *3.71, 3.71 (3H each, both s) and *δ*_C_ 52.0, 52.0 (COO*C*H_3_), and *δ*_C_ 168.6, 168.6 (*C*OOCH_3_)] and a free carboxyl group [*δ*_C_ 170.1 (*C*OOH)] were observed in the ^1^H and ^13^C NMR spectra of **1**. These signals were superimposable with those of sambacoside E (**8**) [21, 25], except for the absence of two methyl ester moieties. Methylation of **1** with trimethylsilyldiazomethane (TMSCHN_2_) produced **8**. Accordingly, the connectivity of the three oleoside ester units to the 5’’,7’’,9’’,10’’-tetraol monoterpene (**21**) moiety in **1** were clarified to be at the C-7’’, C-9’’, and C-10’’ positions.

**Table 1:** ^1^H and ^13^C NMR spectroscopic data (CD_3_OD) of Jasminumoside F (**1**) and sambacoside E (**8**)

Position	**1**		**8** ^a^	
	δ _H_	δ _C_	δ _H_	δ _C_
1/1’’’/1’’’’’	5.92/5.92/5.93 (br s)	95.0/95.6/95.21	5.93 (3 H, br s)	
3/3’’’/3’’’’’	7.49/7.516/7.521 (s)	155.1/155.1/155.1	7.53 (3 H, s)	
4/4’’’/4’’’’’		109.4/109.4/109.4		
5/5’’’/5’’’’’	3.99/3.99/3.99 (m)	31.80/31.89/32.01		
6/6’’’/6’’’’’	2.48 (dd, 8.9, 14.4)/2.49 (dd, 8.9, 14.4)/ 2.49 (dd, 8.9, 14.4)	41.3/41.4/41.4	2.48 (2 H, dd, 9.0, 14.0)/ 2.50 (dd, 9.0, 14.0)	
	2.72 (dd, 4.6, 14.4)/2.73 (dd, 4.6, 14.4)/ 2.76 (dd, 4.6, 14.4)		2.72 (dd, 5.0, 14.0)/ 2.74 (2 H, dd, 5.0, 14.0)	
7/7’’’/7’’’’’		173.1/173.21/173.23		
8/8’’’/8’’’’’	6.10/6.10/6.10 (q, 7.1)	124.6/124.9/125.0	6.11 (3 H, br q, 7.0)	
9/9’’’/9’’’’’		130.67/130.73/131.1		
10/10’’’/10’’’’’	1.74/1.74/1.74 (3 H, d, 6.9)	13.79/13.81/13.81	1.75 (9 H, br d, 6.8)	
11/11’’’/11’’’’’		168.6/170.1/168.6		
COO*CH*_*3*_	3.71/—/3.71 (3 H, s)	52.0/—/52.0	3.71 (9 H, s)	
1’/1’’’’/1’’’’’’	4.81/4.81/4.81 (d, 7.8)	100.79/100.83/100.9	4.81 (3 H, d, 8.0)	
2’/2’’’’/2’’’’’’	3.31/3.31/3.31 (m)	74.7/74.8/74.8		
3’/3’’’’/3’’’’’’	3.33/3.33/3.33 (m)	78.31/78.37/78.39		
4’/4’’’’/4’’’’’’	3.32/3.32/3.32 (m)	71.5/71.5/71.6		
5’/5’’’’/5’’’’’’	3.41/3.41/3.41 (dd, 8.7, 8.7)	78.0/78.0/78.0		
6’/6’’’’/6’’’’’’	3.67/3.67/3.67 (m) 3.88/3.88/3.88 (br d, ca. 12)	62.75/62.75/62.80		
1”	1.65 (m)	46.4		46.5
2’’	1.67 (m)	48.7		49.8
3’’	2.09 (m)	39.4		39.5
4’’	1.70 (m) 1.84 (ddd, 6.4, 6.6, 12.0)	36.9		37.0
5’’	3.69 (m)	79.4		79.4
6’’	1.04 (3 H, d, 6.6)	18.3	1.05 (3 H, d, 6.0)	18.4
7’’	4.01 (dd, 5.0, 11.2) 4.13 (dd, 6.2, 11.2)	68.1		68.2
8’’	2.07 (m)	41.7		41.8
9’’	4.09 (2 H, dd, 6.0, 11.2)	65.6		65.6
10’’	4.03 (dd, 6.2, 11.2) 4.16 (dd, 5.0, 11.2)	64.1		64.2

Measured by 800 MHz for ^1^H NMR and 200 MHz for ^13^C NMR

^a^Reference [25].

**Fig. 3:**
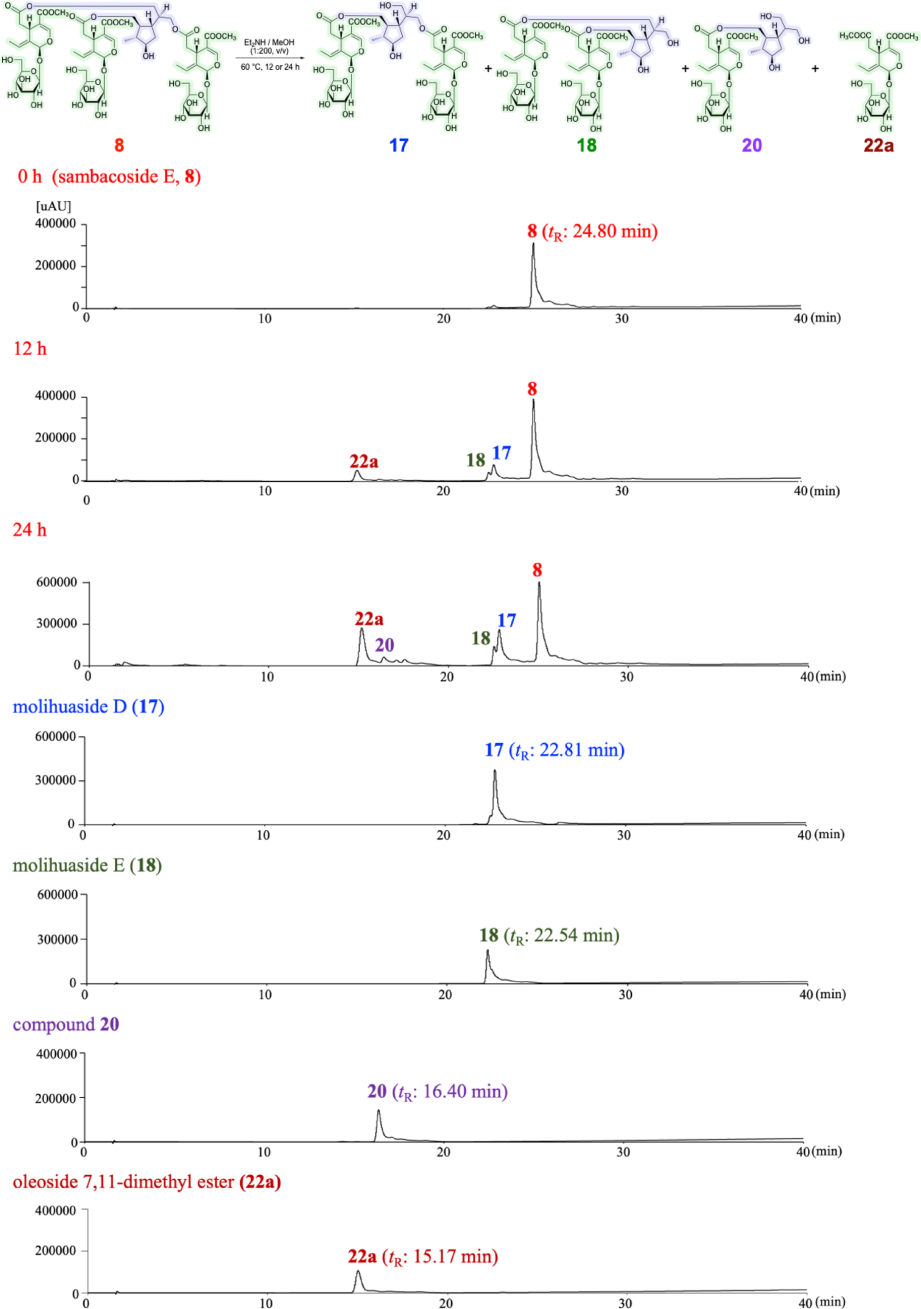
Partial alkaline hydrolysis of **8**. *LC-MS conditions*: Instruments: Thermo Fisher Scientific Ultimate + EvactivePlus, Column: Cosmosil 5C_18_-MS-II (2.0 mm i.d. × 150 mm), HPLC detection: UV (230 nm), Mobile phase: 0–5 min hold MeOH–H_2_O (25:75, v/v) → 40–50 min hold MeOH, Flow rate: 0.2 mL/min, Injection: 2 *µ*L, Ionization mode: ESI, positive

**Fig. 4:**
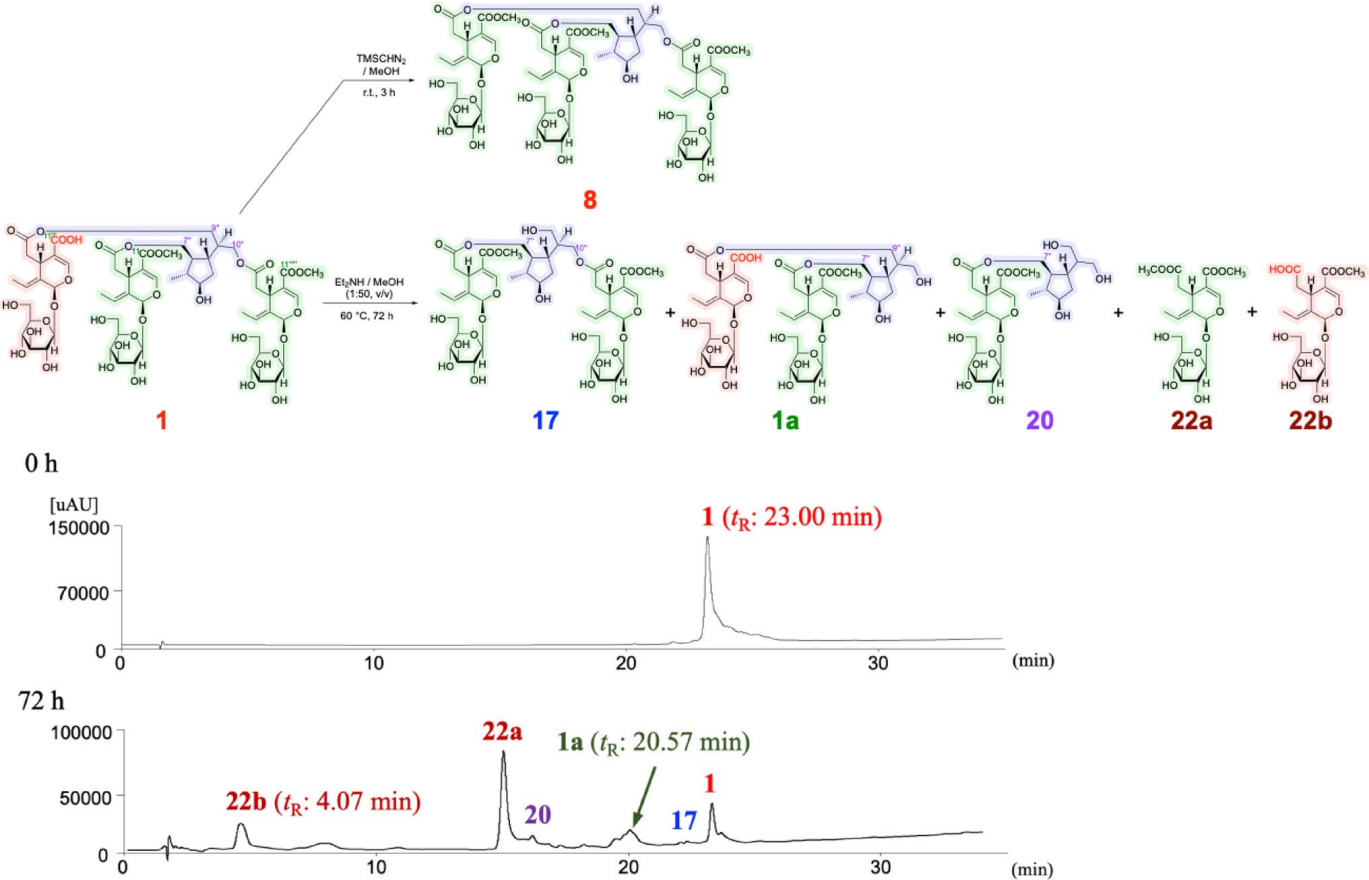
Methylation and partial alkaline hydrolysis of **1**. *LC-MS conditions*: Instruments: Thermo Fisher Scientific Ultimate + EvactivePlus, Column: Cosmosil 5C_18_-MS-II (2.0 mm i.d. × 150 mm), HPLC detection: UV (230 nm), Mobile phase: 0–5 min hold MeOH–H_2_O (25:75, v/v) → 40–50 min hold MeOH, Flow rate: 0.2 mL/min, Injection: 2 *µ*L, Ionization mode: ESI, positive

After treatment of **8** and **1** with diethylamine (Et_2_NH)–MeOH, several partial hydrolysates with common cleaved positions were obtained. As shown in Fig. 3, the 9’’- and 10’’-monodesacyl derivatives, molihuasides D (**17**, *t*_R_: 22.76 min) [21, 28] and E (**18**, *t*_R_: 22.26 min) [21, 28], as well as the 9’’,10’’-bisdeacyl derivative (**20**, *t*_R_: 16.40 min) [21, 29] were identified from **8** together with oleoside 7,11-dimethyl ester (**22a**, *t*_R_: 15.17 min) [21, 30]. The common partial hydrolysates **17**, **20**, and **22a** were also identified upon a similar alkaline hydrolysis of **1** along with the mono-demethyl ester derivative of molihuaside E [**1a**, *t*_R_: 20.57 min, *m/z*: 985.3507 [M + Na]^–^ (calcd for C_43_H_62_O_24_Na, 985.3523)] and oleoside 7-methyl ester (**22b**) [21, 28]. Subsequent methylation of **1a** afforded **18** (Fig. 4). Consequently, the structure of jasminumoside F was determined to be the 11’’’-monodemethyl ester derivative of sambacoside E (**1**) and the absolute configurations were assigned by comparison with previously reported sambacosides and monoterpene (**21**) [21, 28, 29].

#### Jasminumoside G (**2**)

**Fig. 5:**
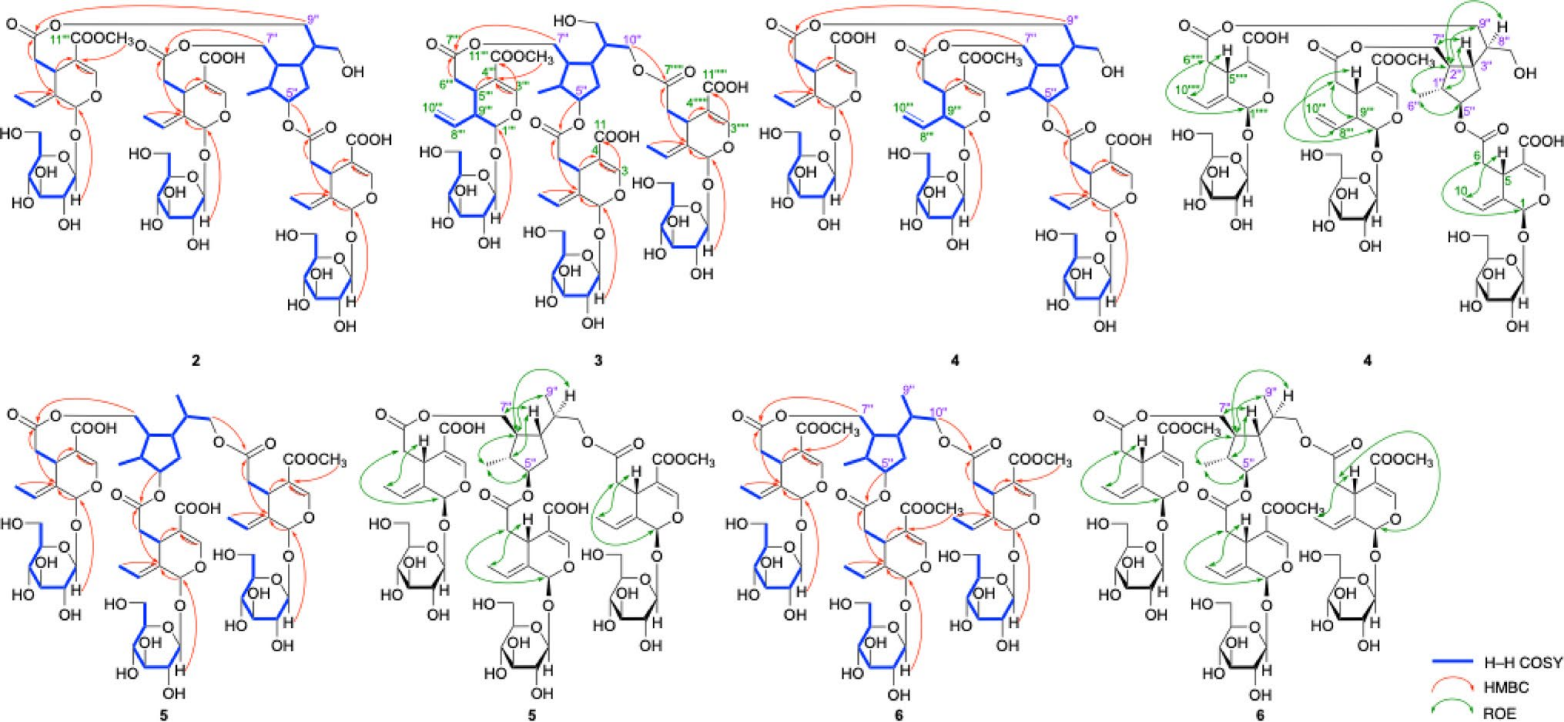
^1^H–^1^H COSY, HMBC, and pROESY correlations of **2**–**6**

**Fig. 6:**
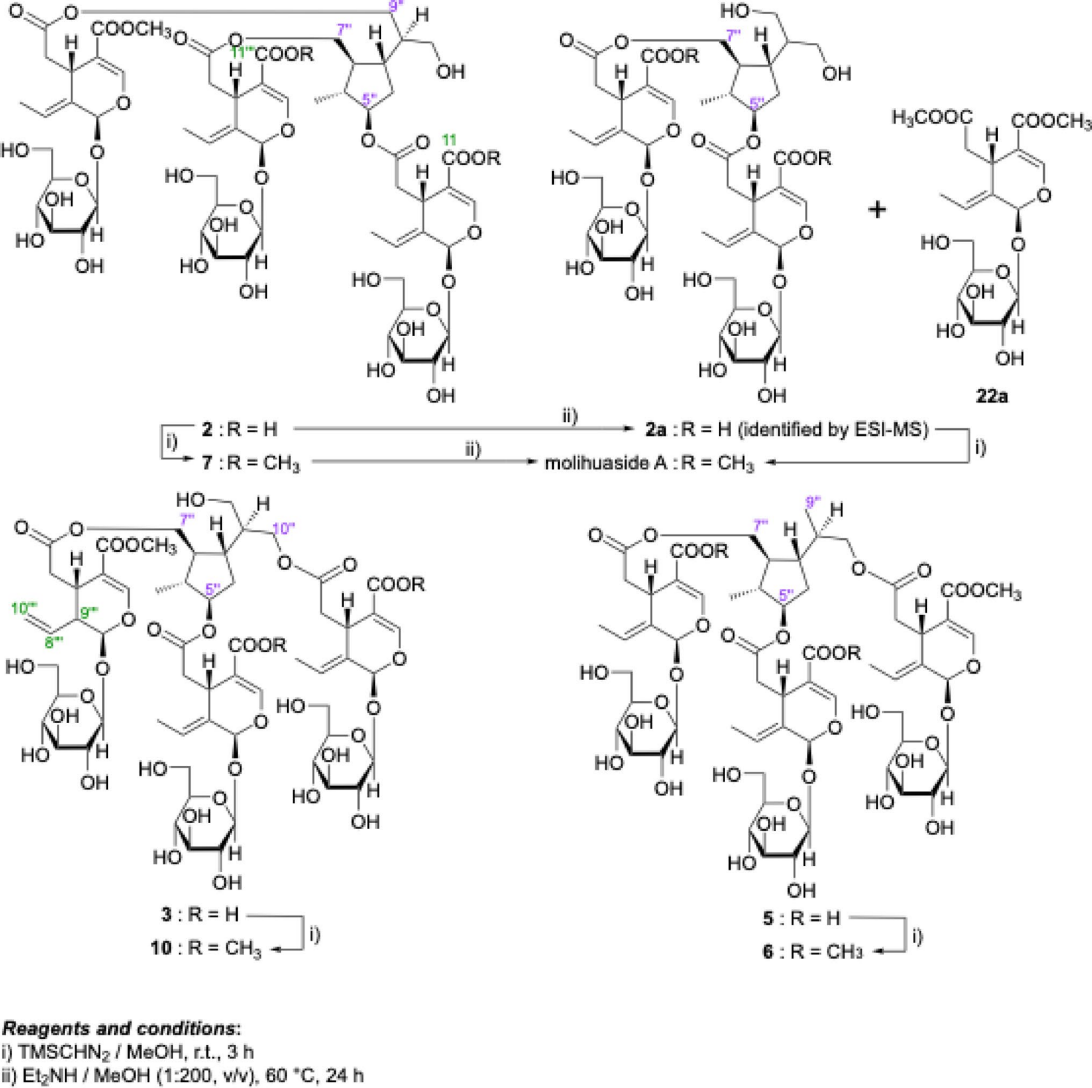
Chemical conversions of **2**, **3**, **5**, and **7**

Jasminumoside G (**2**) was obtained as a white powder with a negative optical rotation ([*α*]_D_^25^ − 173.7 in MeOH). A quasi-molecular ion peak was observed at *m/z* 1357 [M + Na]^+^ by positive-ion ESI-MS analysis, while HRESIMS measurements revealed a molecular formula of C_59_H_82_O_34_. Acid hydrolysis of **2** with 1 M HCl liberated d-glucose. The ^1^H and ^13^C NMR (Table 2) spectroscopic properties of **2**, which were assigned with the aid of the various 2D NMR experiments (Fig. 5), were superimposable with those of sambacoside F (**9**) [25], except for the absence of two carbomethoxy signals, triple signals for the secoiridoid glycosidic ester part {the characteristic iridoid chromophore [*δ *7.52, 7.52, 7.52 (1H each, all s, H-3, 3’’’, 3’’’’’)], methylenes [*δ *2.48 (2 H, dd, *J* = 8.9, 14.2 Hz), 2.49 (1H, dd, *J* = 8.9, 14.2 Hz), 2.65 (2 H, dd, *J* = 4.6, 14.2 Hz), 2.73 (1H, dd, *J* = 4.6, 14.2 Hz), H_2_-6, 6’’’, 6’’’’’], methines [*δ *4.00, 4.00, 4.00 (1H each, all m, H-5, 5’’’, 5’’’’’)], ethylidene groups [*δ *1.75, 1.75, 1.75 (3 H each, all d, *J* = 6.9 Hz, H_3_-10, 10’’’, 10’’’’’), 6.10, 6.10, 6.10 (1H each, all q, *J* = 7.1 Hz, H-8, 8’’’, 8’’’’’)], acetals [*δ *5.92, 5.93, 5.93 (1H each, all br s, H-1, 1’’’, 1’’’’’)], and *β*-glucopyranosyl parts [*δ *4.81, 4.81, 4.82 (1H each, all d, *J* = 7.8 Hz, H-1’, 1’’’’, 1’’’’’’)]} together with the 5’’,7’’,9’’,10’’-tetraol monoterpene (**21**) moiety {{a methyl [*δ *1.05 (3 H, d, *J* = 7.0 Hz, H_3_-6’’)], a methylene and three methylene bearing an oxygen function {*δ *1.88 (2 H, m, H_2_-4’’), [4.06 (1H, dd, *J* = 7.0, 11.4 Hz), 4.16 (1H, dd, *J* = 5.5, 11.4 Hz), H_2_-7’’], [3.58 (1H, dd, *J* = 6.2, 11.4 Hz), 3.66 (1H, m), H_2_-10’’], 4.13 (2 H, d, *J* = 6.4 Hz, H_2_-9’’)}, four methine and a methine bearing an oxygen function [*δ *1.77, 1.89, 1.91, 2.05 (1H each, all m, H-2’’, 8’’, 1’’, 3’’), 4.66 (1H, m, H-5’’)]}}. Additionally, a carbomethoxy signal [*δ *3.71 (3 H, s) and *δ*_C_ 52.1 (COO*C*H_3_), and *δ*_C_ 168.7 (*C*OOCH_3_)] and two free carboxyl groups [*δ*_C_ 170.1, 170.1 (*C*OOH)] were observed in the ^1^H and ^13^C NMR spectra of **2**. Because the methylation of **2** provided **9**, the connectivity of the three ester bonds between the 7-ester carbonyl groups in the three secoiridoid glycoside units and the common monoterpene (**21**) moiety in **2** were determined to be the same as those in **9**. Next, since one of the three secoiridoid glycoside units in **2** exhibited two free carboxyl groups at the 11-position, chemical conversion of **2** into molihuaside A [28] via **2a** was performed. As shown in Fig. 6, partial hydrolysis of **2** with Et_2_NH–MeOH provided the 5’’,7’’-diacyl ester derivative of **21** bearing the secoiridoid glycoside unit with a free carboxyl group (**2a**), which was identified by ESI-MS [*m/z*: 971.3338 [M + Na]^–^ (calcd for C_42_H_60_O_24_Na, 971.3367)] together with **22a**. Subsequently, the TMSCHN_2_ methylation of **2a** provided molihuaside A, which was also obtained upon treatment of **7** with 0.1% NaOMe–MeOH. Based on this evidence, the structure of jasminumoside G was determined to be the 11,11’’’-bisdemethyl ester derivative of sambacoside F (**2**).

**Table 2:** ^1^H and ^13^C NMR spectroscopic data (CD_3_OD) of Jasminumoside G (**2**) and sambacoside F (**9**)

Position	**2**		**9** ^a^	
	δ _H_	δ _C_	δ _H_	δ _C_
1/1’’’/1’’’’’	5.92/5.93/5.93 (br s)	94.9/95.0/95.2	5.93 (3 H, br s)	
3/3’’’/3’’’’’	7.52/7.52/7.52 (s)	155.1/155.2/155.8	7.53 (3 H, s)	
4/4’’’/4’’’’’		109.4/109.4/109.7		
5/5’’’/5’’’’’	4.00/4.00/4.00 (m)	31.85/31.85/31.93		
6/6’’’/6’’’’’	2.48 (dd, 8.9, 14.2)/2.48 (dd, 8.9, 14.2)/ 2.49 (dd, 8.9, 14.2)	41.32/41.33/41.33	2.49 (3 H, dd, 9.0, 14.0)	
	2.65 (dd, 4.6, 14.2)/2.65 (dd, 4.6, 14.2)/ 2.73 (dd, 4.6, 14.2)		2.67 (dd, 5.0, 14.0)/ 2.75 (2 H, dd, 5.0, 14.0)	
7/7’’’/7’’’’’		173.1/173.28/173.29		
8/8’’’/8’’’’’	6.10/6.10/6.10 (q, 7.1)	124.5/124.7/125.0	6.11 (3 H, br q, 7.0)	
9/9’’’/9’’’’’		130.6/130.9/131.0		
10/10’’’/10’’’’’	1.75/1.75/1.75 (3 H, d, 6.9)	13.8/13.8/13.9	1.75 (9 H, br d, 7.0)	
11/11’’’/11’’’’’		168.7/170.1/170.1		
COO*CH*_*3*_	—/—/3.71 (3 H, s)	—/—/52.1	3.72 (9 H, s)	
1’/1’’’’/1’’’’’’	4.81/4.81/4.82 (d, 7.8)	100.7/100.8/100.9	4.82 (3 H, d, 8.0)	
2’/2’’’’/2’’’’’’	3.30/3.30/3.30 (m)	74.7/74.8/74.8		
3’/3’’’’/3’’’’’’	3.33/3.33/3.33 (m)	78.36/78.39/78.43		
4’/4’’’’/4’’’’’’	3.32/3.32/3.32 (m)	71.4/71.47/71.50		
5’/5’’’’/5’’’’’’	3.40/3.40/3.40 (m)	77.9/77.9/77.9		
6’/6’’’’/6’’’’’’	3.66/3.66/3.66 (m) 3.87/3.87/3.87 (m)	62.7/62.77/62.83		
1”	1.91 (m)	44.2		44.3
2’’	1.77 (m)	48.1		49.3
3’’	2.05 (m)	40.2		40.3
4’’	1.88 (2 H, m)	34.8		34.9
5’’	4.66 (m)	83.1	4.66 (m)	83.1
6’’	1.05 (3 H, d, 7.0)	18.9	1.06 (3 H, d, 7.0)	18.9
7’’	4.06 (dd, 7.0, 11.4) 4.16 (dd, 5.5, 11.4)	68.2		68.2
8’’	1.89 (m)	44.3		44.3
9’’	4.13 (2 H, d, 6.4)	65.7		65.8
10’’	3.58 (dd, 6.2, 11.4) 3.66 (m)	60.9		61.0

Measured by 800 MHz for ^1^H NMR and 200 MHz for ^13^C NMR

^a^Reference [25].

#### Jasminumosides H (**3**) and I (**4**)

Jasminumosides H (**3**) and I (**4**) were also obtained as white powders with negative optical rotations (**3**: [*α*]_D_^25^ − 161.5, **4**: [*α*]_D_^25^ − 162.2 both in MeOH). In the positive- and negative-mode ESI-MS spectra of **3** and **4**, similar quasi-molecular ion peaks were observed at *m/z* 1357 [M + Na]^+^ and *m/z* 1333 [M – H]^–^, while the molecular formula C_59_H_82_O_34_ was determined by HRESIMS measurements. Acid hydrolysis of **3** and **4** liberated d-glucose. The ^1^H and ^13^C NMR spectra (Table 3) of **3** showed triple protons signals for the characteristic iridoid chromophore (*vide ante*) [*δ *7.47 (1H, s, H-3’’’), 7.511, 7.517 (1H each, both s, H-3, 3’’’’’)], methylenes {[*δ *2.34 (1H, dd, *J* = 8.7, 16.1 Hz), 2.91 (1H, dd, *J* = 5.3, 16.1 Hz), H_2_-6’’’], 2.48 (1H, dd, *J* = 8.9, 14.3 Hz), 2.50 (1H, dd, *J* = 8.9, 14.4 Hz), 2.69 (1H, dd, *J* = 4.4, 14.3 Hz), 2.77 (1H, dd, *J* = 5.0, 14.4 Hz), H_2_-6, 6’’’’’]}, methines [*δ *3.33 (1H, m, H-5’’’), 3.98, 3.98 (1H each, both m, H-5, 5’’’’’)], two ethylidene and a mono-substituted olefin groups {*δ *[1.74, 1.74 (3H each, both d, *J* = 6.9 Hz, H_3_-10, 10’’’’’), 6.10, 6.10 (1H each, both q, *J* = 7.1 Hz, H-8, 8’’’’’)], [5.25 (2H, m, H-10’’’), 5.63 (1H, ddd, *J* = 9.6, 10.1, 17.1 Hz, H-8’’’)]}, acetals [*δ *5.47 (1H, d. *J* = 4.1 Hz, H-1’’’), 5.92, 5.92 (1H each, both br s, H-1, 1’’’’’)], and *β*-glucopyranosyl parts [*δ *4.66 (1H, d, *J* = 8.0 Hz, H-1’’’’), 4.81, 4.82 (1H each, both d, *J* = 7.8 Hz, H-1’, 1’’’’’)] along with signals due to the common monoterpene (**21**) moiety {{a methyl [*δ *1.05 (3H, d, *J* = 7.1 Hz, H_3_-6’’)], a methylene and three methylene bearing an oxygen function {*δ *1.74, 1.86 (1H each, both m, H_2_-4’’), 3.59 (2H, dd, *J* = 6.4, 11.2 Hz, H_2_-9’’), [4.01 (1H, dd, *J* = 6.4, 11.2 Hz), 4.21 (1H, dd, *J* = 4.8, 11.2 Hz), H_2_-10’’], [4.05 (1H, dd, *J* = 6.4, 11.2 Hz), 4.20 (1H, dd, *J* = 4.6, 11.2 Hz), H_2_-7’’], four methine and a methine bearing an oxygen function [*δ *1.75, 1.86, 1.92, 2.10 (1H each, all m, H-2’’, 8’’, 1’’, 3’’), 4.64 (1H, m, H-5’’)]}}. Additionally, a carbomethoxy signals [*δ *3.67 (3H, s) and *δ*_C_ 52.1 (COO*C*H_3_), and *δ*_C_ 168.8 (*C*OOCH_3_)] and two free carboxyl group [*δ*_C_ 170.1, 170.1 (*C*OOH)] were observed in the ^1^H and ^13^C NMR spectra of **3**. As shown in Fig. 5, the ^1^H-^1^H COSY spectrum of **3** indicated the presence of partial structures, as denoted by the thick lines. In the HMBC spectrum of **3**, long-range correlations were observed between the H-5’’ and H_2_-9’’ protons and the ester carbonyl carbons assignable to two oleoside units [*δ*_C_ 170.1, 170.1 (C-7, 7’’’’’)], and between the H_2_-7’’ protons and the ester carbonyl carbons assignable to the secologanoside 11-methyl ester unit, which were quite similar to those of molihuaside B (**10**) [*δ *3.68 (3H, s) and *δ*_C_ 51.8 (COO*C*H_3_), and *δ*_C_ 168.8 (C-7’’’) [28]. Finally, the methylation of **3** provided **10**), resulting in the connectivities of the ester bonds between the 7-ester carbonyl groups in the two oleoside units and the 5’’- and 10’’-positions and between the 7-ester carbonyl part in a secologanoside 11-methyl ester unit and the 7’’-position in **21**. Therefore, the structure of jasminumoside J was determined to be the 11,11’’’’’-bisdemethyl ester derivative of molihuaside B (**3**). The proton and carbon signals in the ^1^H and ^13^C NMR spectra (Table 4) of **4**, which were assigned with the aid of various 2D NMR experiments (Fig. 5), were indicative of the same units as those of **21**. Furthermore, by comparing the ^13^C NMR data for **4** with those of **21**, the signals due to C-5’’ (*δ*_C_ 83.1), C-7’’ (*δ*_C_ 67.9), and C-9’’ (*δ*_C_ 65.6) in **4** were observed at lower fields compared to those of **21** [*δ*_C_ C-5’’ (*δ*_C_ 79.6), C-7’’ (*δ*_C_ 66.1), and C-9’’ (*δ*_C_ 63.1)]. In contrast, the signals due to C-1’’ (*δ*_C_ 44.2), C-2’’ (*δ*_C_ 49.1), C-4’’ (*δ*_C_ 35.0), and C-8’’ (*δ*_C_ 44.4) were observed at higher fields compared with those of **21** [C-1’’ (*δ*_C_ 46.2), C-2’’ (*δ*_C_ 52.0), C-4’’ (*δ*_C_ 37.7), and C-8’’ (*δ*_C_ 48.4)] [21, 29]. Based on these acylation shifts, the connectivities of the secoiridoid ester moieties at the C-5’’, C-7’’, and C-9’’ positions were determined, as shown in Table 4. The stereostructure was characterized by rotating-frame nuclear Overhauser enhancement spectroscopy (ROESY), which revealed rotating-frame nuclear Overhauser effect (ROE) correlations between the following proton pairs: H-1’’ [*δ* 1.91 (1H, m)] and H-3’’ [*δ* 2.05 (1H, m)]; H-2’’ [*δ* 1.77 (1H, m)] and H_3_-6’’ [*δ* 1.05 (3H, d, *J* = 7.0 Hz)], H-8’’ [*δ* 1.89 (1H, m)], H_2_-9’’ [*δ* 4.13 (2H, d, *J* = 6.2 Hz)]; H-3’’ and H-5’’ [*δ* 4.66 (1H, m)], H_2_-7’’ [*δ* 4.05 (1H, dd, *J* = 7.0, 11.2 Hz), 4.15 (1H, dd, *J* = 5.5, 11.2 Hz)]; H-5’’ and H_3_-6’’ (Fig. 5). Thus, the structure of jasminumoside I (**4**) was elucidated.

**Table 3:** ^1^H and ^13^C NMR spectroscopic data (CD_3_OD) of Jasminumoside H (**3**) and Molihuaside B (**10**)

Position	**3**		**10** ^a^	
	δ _H_	δ _C_	δ _H_	δ _C_
1/1’’’’’	5.92/5.92 (br s)	95.0/95.1	5.93/5.94 (s)	95.2
3/3’’’’’	7.511/7.517 (s)	154.9/154.9	7.53/7.54 (s)	155.1
4/4’’’’’		110.0/110.0		109.5
5/5’’’’’	3.98/3.98 (m)	31.9/32.0		32.0
6/6’’’’’	2.48 (dd, 8.9, 14.3)/2.50 (dd, 8.9, 14.4)	41.3/41.3		41.3
	2.69 (dd, 4.4, 14.3)/2.77 (dd, 5.0, 14.4)			
7/7’’’’’		173.1/173.3		173.0
8/8’’’’’	6.10/6.10 (q, 7.1)	124.5/124.6	6.11 (2 H, br q, 7.0)	124.9
9/9’’’’’		130.9/131.1		130.8
10/10’’’’’	1.74/1.74 (3 H, d, 6.9)	13.76/13.80	1.75 (6 H, d, 7.0)	13.8
11/11’’’’’		170.1/170.1		168.6
COO*CH*_*3*_			3.72 (6 H, s)	52.0
1’/1’’’’’’	4.81/4.82 (d, 7.8)	100.8/100.9	4.81/4.82 (d, 8.0)	100.9
2’/2’’’’’’	3.31/3.31 (m)	74.8/74.8		74.6
3’/3’’’’’’	3.33/3.33 (m)	78.39/78.41		78.4
4’/4’’’’’’	3.32/3.32 (m)	71.51/71.54		71.6
5’/5’’’’’’	3.41/3.41 (dd, 8.7, 8.7)	77.96/77.96		78.0
6’/6’’’’’’	3.67/3.67 (m) 3.88/3.88 (br d, ca. 12)	62.7/62.8		62.8
1’’’	5.47 (d, 4.1)	97.6	5.48 (d, 4.0)	97.6
3’’’	7.47 (s)	153.8	7.48 (d, 1.6)	153.7
4’’’		110.0		110.0
5’’’	3.33 (m)	29.0		29.1
6’’’	2.34 (dd, 8.7, 16.1)	35.6		35.6
	2.91 (dd, 5.3, 16.1)			
7’’’		174.3		174.3
8’’’	5.63 (ddd, 9.6, 10.1, 17.1)	134.5	5.64 (m)	135.6
9’’’	2.78 (m)	131.1		45.4
10’’’	5.25 (2 H, m)	120.7	5.25 (2 H, dd, 10, 18)	120.7
11’’’		168.8		168.8
COO*CH*_*3*_	3.67 (3 H, s)	52.1	3.68 (3 H, s)	51.8
1’’’’	4.66 (d, 8.0)	100.0	4.66 (d, 8.0)	100.1
2’’’’	3.31 (m)	74.6		74.8
3’’’’	3.33 (m)	78.41		78.4
4’’’’	3.32 (m)	71.42		71.6
5’’’’	3.41 (dd, 8.7, 8.7)	78.0		78.0
6’’’’	3.67 (m) 3.89 (br d, ca. 12)	62.82		62.9
1”	1.92 (m)	44.0		44.2
2’’	1.75 (m)	49.1		49.7
3’’	2.10 (m)	39.9		40.0
4’’	1.74 (m) 1.86 (m)	34.6		34.7
5’’	4.64 (m)	83.1		83.2
6’’	1.05 (3 H, d, 7.1)	18.8	1.06 (3 H, d, 7.0)	18.9
7’’	4.05 (dd, 6.4, 11.2) 4.20 (dd, 4.6, 11.2)	67.5		67.6
8’’	1.86 (m)	44.1		44.1
9’’	3.59 (2 H, dd, 6.4, 11.2)	62.6		62.6
10’’	4.01 (dd, 6.4, 11.2) 4.21 (dd, 4.8, 11.2)	64.2		64.4

Measured by 800 MHz for ^1^H NMR and 200 MHz for ^13^C NMR

^a^Reference [28].

**Table 4:** ^1^H and ^13^C NMR spectroscopic data (CD_3_OD) of Jasminumoside I (**4**) and **21**

Position	**4**		**21** ^a^	Δδ_C_ (**21**–**4**)^b^
	δ _H_	δ _C_	δ _C_	
1/1’’’’’	5.92/5.92 (br s)	95.08/95.13		
3/3’’’’’	7.47/7.47 (s)	153.8/153.8		
4/4’’’’’		110.0/110.0		
5/5’’’’’	3.99/3.99 (m)	31.6/32.0		
6/6’’’’’	2.48 (dd, 8.9, 14.2)/2.49 (dd, 8.9, 14.2)	41.3/41.4		
	2.65 (dd, 4.6, 14.2)/2.73 (dd, 4.6, 14.2)			
7/7’’’’’		173.1/173.4		
8/8’’’’’	6.10/6.10 (q, 7.1)	124.4/124.6		
9/9’’’’’		131.0/131.2		
10/10’’’’’	1.75/1.75 (3 H, d, 6.9)	13.8/13.8		
11/11’’’’’		170.1/170.1		
1’/1’’’’’’	4.81/4.82 (d, 7.8)	100.8/100.9		
2’/2’’’’’’	3.30/3.30 (m)	74.6/74.78		
3’/3’’’’’’	3.33/3.33 (m)	78.37/78.39		
4’/4’’’’’’	3.32/3.32 (m)	71.5/71.6		
5’/5’’’’’’	3.40/3.40 (m)	77.97/78.04		
6’/6’’’’’’	3.66/3.66 (m) 3.87/3.87 (m)	62.74/62.79		
1’’’	5.47 (d, 4.2)	97.6		
3’’’	7.50 (s)	154.6		
4’’’		110.0		
5’’’	3.30 (m)	29.0		
6’’’	2.35 (dd, 8.5, 16.1)	35.6		
	2.90 (dd, 5.3, 16.1)			
7’’’		174.4		
8’’’	5.63 (ddd, 9.6, 10.1, 17.1)	134.6		
9’’’	2.77 (m)	45.4		
10’’’	5.25 (2 H, m)	120.7		
11’’’		168.6		
COO*CH*_*3*_	3.71 (3 H, s)	51.9		
1’’’’	4.66 (d, 8.0)	100.1		
2’’’’	3.30 (m)	74.79		
3’’’’	3.33 (m)	78.43		
4’’’’	3.32 (m)	71.6		
5’’’’	3.40 (m)	78.2		
6’’’’	3.66 (m) 3.87 (m)	62.81		
1”	1.91 (m)	44.2	46.2	+ 2.0
2’’	1.77 (m)	49.1	52.0	+ 2.9
3’’	2.05 (m)	40.1	38.2	– 1.9
4’’	1.90 (2 H, m)	35.0	37.7	+ 2.7
5’’	4.66 (m)	83.1	79.6	– 3.5
6’’	1.05 (3 H, d, 7.0)	18.8	18.6	– 0.2
7’’	4.05 (dd, 7.0, 11.2) 4.15 (dd, 5.5, 11.2)	67.9	66.1	– 1.8
8’’	1.89 (m)	44.4	48.4	+ 4.0
9’’	4.13 (2 H, d, 6.2)	65.6	63.1	– 2.5
10’’	3.58 (dd, 6.2, 11.1) 3.67 (m)	61.0	62.5	+ 1.5

Measured by 800 MHz for ^1^H NMR and 200 MHz for ^13^C NMR

^a^Reference [29].

^b^By means of acylation shift, down-field and up-field shifts were observed by comparison of the ^13^C NMR data for **4** with those of **21**

#### Jasminumosides J (**5**) and K (**6**)

Jasminumoside J (**5**) was obtained as a white powder with a negative optical rotation ([*α*]_D_^25^ − 171.8 in MeOH). The molecular formula of C_59_H_82_O_33_ was confirmed by HRESIMS measurements [*m/z* 1341.4614 [M + Na]^+^ (calcd for C_59_H_82_O_33_Na, 1341.4631)]. Acid hydrolysis of **5** liberated d-glucose, as identified by HPLC analysis. The ^1^H and ^13^C NMR (Table 5) spectra of **5** contained signals assignable to triplet signals for the oleoside ester part {[*δ *7.51, 7.53, 7.53 (1H each, all s, H-3, 3’’’, 3’’’’’)], methylenes [*δ *2.50 (1H, dd, *J* = 8.5, 14.2 Hz), 2.51 (1H, dd, *J* = 8.7, 14.0 Hz), 2.65 (1H, dd, *J* = 8.6, 14.0 Hz), 2.65 (1H, dd, *J* = 4.6, 14.2 Hz), 2.71 (1H, dd, *J* = 4.8, 14.2 Hz), 2.76 (1H, dd, *J* = 4.6, 14.0 Hz), H_2_-6, 6’’’, 6’’’’’], methines [*δ *3.99, 3.99, 3.99 (1H each, all m, H-5, 5’’’, 5’’’’’)], ethylidene groups [*δ *1.74, 1.74, 1.74 (3 H each, all d, *J* = 6.9 Hz, H_3_-10, 10’’’, 10’’’’’), 6.10, 6.10, 6.10 (1H each, all q, *J* = 6.4 Hz, H-8, 8’’’, 8’’’’’)], acetals [*δ *5.91, 5.92, 5.92 (1H each, all br s, H-1, 1’’’, 1’’’’’)], and *β*-glucopyranosyl moieties [*δ *4.80, 4.81, 4.81 (1H each, all d, *J* = 7.8 Hz, H-1’, 1’’’’, 1’’’’’’)]} together with the monoterpene triol moiety {{two methyl [*δ *1.02 (3 H, d, *J* = 6.6 Hz, H_3_-9’’), 1.03 (3 H, d, *J* = 7.1 Hz, H_3_-6’’)], a methylene and two methylene bearing an oxygen function {*δ *1.78, 1.87 (1H each, both m, H_2_-4’’), [3.82 (1H, dd, *J* = 6.6, 11.0 Hz), 4.20 (1H, dd, *J* = 4.8, 11.0 Hz), H_2_-10’’], [3.93 (1H, dd, *J* = 7.8, 10.5 Hz), 4.21 (1H, dd, *J* = 4.1, 10.5 Hz), H_2_-7’’]}, four methine and a methine bearing an oxygen function [*δ *1.68, 1.94, 1.85, 1.85, 4.65 (1H each, all m, H-2’’, 1’’, 8’’, 3’’, 5’’)]}}. Additionally, carbomethoxy signals [*δ *3.71 (3 H, s) and *δ*_C_ 52.0 (COO*C*H_3_), and *δ*_C_ 168.6 (*C*OOCH_3_)] were observed in the ^1^H and ^13^C NMR spectra of **5** along with signals from two free carboxyl groups [*δ*_C_ 170.2, 170.2 (*C*OOH)]. The ^1^H and ^13^C NMR spectroscopic properties of **5** were very similar to those of sambacoside A (**7**) [21, 25], except for the signals due to the 9’’-hydroxymethyl group in the monoterpene moiety of **7** being replaced by a methyl group and absence of two carbomethoxy signals, which were assigned according to ^1^H–^1^H COSY, HMBC, and ROESY analyses (Fig. 5). The molecular formula of jasminumoside K (**6**) was determined to be C_61_H_86_O_33_ using positive- and negative-mode ESI-MS and HR-ESI-MS measurements. The signals in the ^1^H and ^13^C NMR (Table 5) spectra of **6** were compared with those of **5** were observed quite similar except for the number of carbomethoxy signals [*δ *3.71, 3.71, 3.72 (3 H each, all s) and *δ*_C_ 52.0, 52.0, 52.0 (COO*C*H_3_), and *δ*_C_ 168.6, 168.6, 168.6 (*C*OOCH_3_)]. Finally, the methylation of **5** provided **6**, enabling to identify the structure of **6** as that of the 9’’-dehydroxy analog of sambacoside A.

**Table 5:** ^1^H and ^13^C NMR spectroscopic data (CD_3_OD) of Jasminumosides J (**5**) and K (**6**)

Position	**5**		**6**	
	δ_H_	δ_C_	δ_H_	δ_C_
1/1’’’/1’’’’’	5.92/5.92/5.91 (brs)	95.0/95.2/95.2	5.93/5.93/5.93 (br s)	95.1/95.1/95.2
3/3’’’/3’’’’’	7.51/7.53/7.53 (s)	154.9/155.1/155.2	7.53/7.53/7.53 (s)	155.11/155.14/155.14
4/4’’’/4’’’’’		109.4/110.0/110.0		109.4/109.4/109.4
5/5’’’/5’’’’’	3.99/3.99/3.99 (m)	31.89/31.94/32.0	3.99/3.99/3.99 (m)	31.88/31.91/31.91
6/6’’’/6’’’’’	2.50 (dd, 8.5, 14.2)/2.51 (dd, 8.7, 14.0)/2.65 (dd, 8.6, 14.0)	41.17/41.22/41.3	2.47 (dd, 9.3, 14.2)/2.50 (dd, 8.5, 14.2)/2.51 (dd, 8.7, 14.0)	41.25/41.31/41.34
	2.65 (dd, 4.6, 14.2)/2.71 (dd, 4.8,14.2)/2.76 (dd, 4.6, 14.0)		2.66 (dd, 4.5, 14.2)/2.71 (dd, 4.8, 14.0)/2.72 (dd, 4.8, 14.2)	
7/7’’’/7’’’’’		173.18/173.25/173.31		173.0/173.2/173.3
8/8’’’/8’’’’’	6.10/6.10/6.10 (q, 6.4)	124.5/124.8/124.8	6.10/6.10/6.10 (q, 6.7)	124.7/124.8/124.8
9/9’’’/9’’’’’		130.8/131.2/131.2		130.8/130.8/131.19
10/10’’’/10’’’’’	1.74/1.74/1.74 (3 H, d, 6.9)	13.7/13.8/13.8	1.74/1.74/1.74 (3 H, d, 6.9)	13.75/13.78/13.78
11/11’’’/11’’’’’		170.2/170.2/168.6		168.6/168.6/168.6
COO*CH*_*3*_	—/—/3.71 (3 H, s)	—/—/52.0	3.71/3.71/3.72 (3 H, s)	52.0/52.0/52.0
1’/1’’’’/1’’’’’’	4.80/4.81/4.81 (d, 7.8)	100.7/100.8/100.9	4.80/4.80/4.80 (d, 7.8)	100.7/100.8/100.8
2’/2’’’’/2’’’’’’	3.31/3.31/3.31 (m)	74.8/74.8/74.8	3.31/3.31/3.31 (m)	74.8/74.8/74.8
3’/3’’’’/3’’’’’’	3.33/3.33/3.33 (m)	78.4/78.4/78.5	3.33/3.33/3.33 (m)	78.4/78.4/78.5
4’/4’’’’/4’’’’’’	3.30/3.30/3.30 (m)	71.54/71.58/71.61	3.30/3.30/3.30 (m)	71.5/71.5/71.6
5’/5’’’’/5’’’’’’	3.41/3.41/3.41 (ddd, 2.8, 8.8, 9.2)	78.0/78.0/78.0	3.40/3.40/3.40 (ddd, 2.3, 9.1, 9.1)	77.9/77.9/77.9
6’/6’’’’/6’’’’’’	3.67/3.67/3.67 (m) 3.88/3.88/3.88 (br d, ca. 12)	62.7/62.8/62.9	3.66/3.66/3.66 (m) 3.87/3.87/3.87 (dd, 1.5, 12.0)	62.8/62.8/62.9
1”	1.94 (m)	44.3	1.95 (m)	44.3
2’’	1.68 (m)	48.5	1.69 (m)	48.5
3’’	1.85 (m)	43.6	1.85 (m)	43.6
4’’	1.78 (m) 1.87 (m)	35.3	1.79 (m) 1.85 (m)	35.3
5’’	4.65 (m)	83.1	4.65 (m)	83.2
6’’	1.03 (3 H, d, 7.1)	19.0	1.04 (3 H, d, 7.1)	19.0
7’’	3.93 (dd, 7.8, 10.5) 4.21 (dd, 4.1, 10.5)	68.5	3.93 (dd, 7.8, 11.0) 4.20 (dd, 4.9, 11.0)	68.6
8’’	1.85 (m)	37.5	1.85 (m)	37.5
9’’	1.02 (3 H, d, 6.6)	16.4	1.02 (3 H, d,6.4)	16.4
10’’	3.82 (dd, 6.6, 11.0) 4.20 (dd, 4.8, 11.0)	69.1	3.83 (dd, 6.7, 11.0) 4.10 (dd, 4.7, 11.0)	69.0

Measured by 800 MHz for ^1^H NMR and 200 MHz for ^13^C NMR

## Conclusion

In conclusion, six new oligomeric secoiridoid glycosides, jasminumosides F–K (**1**–**6**), were isolated from the methanol extract of jasmine, the flower of *J. sambac*. We have previously found that several oligomeric secoiridoid glycosides, sambacoside A (**7**) and molihuasides C (**16**) and D (**17**), from this plant material exhibited the hepatoprotective activity [21]. Due to the structural similarity, newly obtained new oligomeric secoiridoid glycosides, jasminumosides F–K (**1**–**6**), in this study are expected to have similar effects. The detailed structural requirements of secoiridoids for the hepatoprotective effect need to be elucidated further.

## Materials and methods

### General

The following instruments were used to obtain physical data: specific rotations, Horiba SEPA-300 digital polarimeter (*l* = 5 cm); UV spectra, Shimadzu UV-1600 spectrometer; IR spectra, Shimadzu FTIR-8100 spectrometer; ^1^H NMR spectra, JNM-ECA800 (800 MHz), JNM-ECS400 and JNM-AL400 (400 MHz) spectrometers; ^13^C NMR spectra, JNM-ECA800 (200 MHz), JNM-ECS400 and JNM-AL400 (100 MHz) spectrometers with tetramethylsilane as an internal standard; ESIMS and HRESIMS, Exactive Plus mass spectrometer (Thermo Fisher Scientific Inc., Waltham, MA, USA); HPLC detectors, Shimadzu RID-6 A refractive index (RI) and SPD-10 A UV–Vis detector (detection: 230 nm) and Shodex OR-2 optical rotation detector; HPLC column, Cosmosil 5C_18_-MS-II (Nacalai Tesque, Inc., Kyoto, Japan) and Wakopak Navi C30-5 (FUJIFILM Wako Pure Chemical Co., Osaka, Japan), 4.6 mm i.d. ⋅ 250 mm and 20 mm i.d. ⋅ 250 mm) for analytical and preparative purposes, respectively. In addition, Kaseisorb LC NH_2_−60-5 (Tokyo Kasei Co., Ltd., Tokyo, Japan, 4.6 mm i.d. ⋅ 250 mm) was used to identify the sugar part.

The following experimental conditions were used for column chromatography (CC): highly porous synthetic resin, Diaion HP-20 (Mitsubishi Chemical Co., Tokyo, Japan); normal-phase silica gel CC, silica gel 60 N (Kanto Chemical Co., Ltd., Tokyo, Japan; 63–210 mesh, spherical, neutral); reversed-phase ODS CC, Chromatorex ODS DM1020T (Fuji Silysia Chemical, Ltd., Aichi, Japan; 100–200 mesh); TLC, pre-coated TLC plates with silica gel 60F_254_ (Merck, Darmstadt, Germany, 0.25 mm) (normal-phase) and silica gel RP-18 WF_254S_ (Merck, Darmstadt, Germany, 0.25 mm) (reversed-phase); reversed-phase HPTLC, pre-coated TLC plates with silica gel RP-18 WF_254S_ (Merck, Darmstadt, Germany, 0.25 mm); detection was carried out by spraying 1% Ce(SO_4_)_2_–10% aqueous H_2_SO_4_, followed by heating.

### Plant material

*J. sambac* flowers were cultivated in the Guangxi Zhuang Autonomous Region, China, as previously described [21]. The plant material was identified by one of the authors (T. M.), and a voucher specimen of this plant was deposited in our laboratory.

### Extraction and isolation

Dried flowers of *J. sambac* (3.00 kg) were extracted three times with MeOH (5 L) under reflux for 3 h. Subsequent evaporation of the solvent under reduced pressure provided the MeOH extract (1086.19 g, 36.21%). An aliquot (886.19 g) was partitioned with EtOAc–H_2_O (1:1, v/v) to obtain an EtOAc-soluble fraction (228.60 g, 9.34%) and an aqueous phase. The latter was subjected to Diaion HP-20 CC (3.0 kg, H_2_O → MeOH) to yield H_2_O-eluted (402.23 g, 16.43%) and MeOH-eluted (255.00 g, 10.42%) fractions. The MeOH-eluted fraction (186.00 g) was subjected to normal-phase silica gel CC [3.5 kg, CHCl_3_–MeOH–H_2_O (10:3:1 → 7:3:0.5 → 6:4:1, v/v/v) → MeOH] to afford seven fractions [Fr. 1 (2.79 g), Fr. 2 (32.40 g), Fr. 3 (30.28 g), Fr. 4 (59.30 g), Fr. 5 (4.84 g), Fr. 6 (40.23 g), and Fr. 7 (16.10 g)]. Fraction 2 (32.40 g) was subjected to reverse-phase ODS CC [195 g, MeOH–H_2_O (10:90 → 30:70 → 50:50 → 70:30, v/v) → MeOH] to yield 13 fractions [Fr. 2-1 (1.13 g), Fr. 2-2 (3.27 g), Fr. 2-3 (0.37 g), Fr. 2-4 (2.10 g), Fr. 2-5 (0.73 g), Fr. 2-6 (0.28 g), Fr. 2-7 (0.34 g), Fr. 2-8 (5.87 g), Fr. 2-9 (3.87 g), Fr. 2-10 (0.56 g), Fr. 2-11 (0.11 g), Fr. 2-12 (0.68 g), and Fr. 2-13 (12.50 g)], as previously described [21]. Fraction 2-12 (359.4 mg) was subjected to HPLC [detection: UV (230 nm), column: Cosmosil 5C_18_-MS-II, MeOH–1% aqueous AcOH (55:45, v/v)] to yield jasminumoside K (**6**, 18.4 mg, 0.0039%). Fraction 6 (40.2 g) was subjected to reverse-phase ODS CC [1.20 kg, MeOH–H_2_O (20:80 → 40:60 → 60:40, v/v) → MeOH] to yield seven fractions [Fr. 6-1 (1.13 g), Fr. 6-2 (18.00 g), Fr. 6-3 (7.69 g), Fr. 6-4 (3.52 g), Fr. 6-5 (2.15 g), Fr. 6-6 (0.58 g), and Fr. 6-7 (6.70 g)], as previously described [21]. Fraction 6-3 (498.3 mg) was subjected to HPLC [detection: UV (230 nm), column: Cosmosil 5C_18_-MS-II, MeOH–1% aqueous AcOH (35:65, v/v)] to afford six fractions {Fr. 6-3-1 (106.8 mg), Fr.6-3-2 (8.3 mg), Fr. 6-3-3 (48.6 mg), Fr. 6-3-4 (21.7 mg), Fr. 6-3-5 [= jasminumoside G (**2**, 28.8 mg, 0.0249%)], and Fr. 6-3-6 (149.4 mg)}. Fraction 6 − 4 (525.1 mg) was subjected to HPLC [detection: UV (230 nm), column: Cosmosil 5C_18_-MS-II, MeOH–1% aqueous AcOH (40:60, v/v)] to afford five fractions {Fr. 6-4-1 (73.7 mg), Fr. 6-4-2 [= jasminumoside H (**3**, 55.7 mg, 0.0209%)], Fr. 6-4-3 (33.1 mg), Fr. 6-4-4 [jasminumoside F (**1**, 28.2 mg, 0.0106%)], and Fr. 6-4-5 [= jasminumoside J (**5**, 22.1 mg, 0.0083%)]}. Fr. 6-4-3 (33.1 mg) was further subjected to HPLC [detection: UV (230 nm), column: Cosmosil 5C_18_-MS-II, CH_3_CN–1% aqueous AcOH (20:80, v/v)] to provide jasminumoside I (**4**, 10.5, 0.0039%).

### Jasminumoside F (**1**)

White powder, [*α*]_D_^25^ − 156.0 (*c* 0.80, MeOH); UV [MeOH, nm (long *ε*)]: 235 (4.46); IR (KBr) *v*_max_: 3308, 1732, 1717, 1699, 1636, 1076 cm^–1^ (Figure S1); ^1^H (800 MHz, CD_3_OD) and ^13^C NMR (200 MHz, CD_3_OD): data shown in Table 1 (Figures S2 and S3); ^1^H–^1^H COSY, HMQC, HMBC, and pROESY spectra are provided in the Supporting Information (Figures S4–S7); positive-ion ESI-MS *m/z*: 1371 [M + Na]^+^; HRESIMS *m/z*: 1371.4723 [M + Na]^+^ (calcd for C_60_H_84_O_34_Na, 1371.4736) (Figure S8); negative-ion ESI-MS *m/z*: 1347 [M – H]^–^; HRESIMS *m/z*: 1347.4779 [M – H]^–^ (calcd for C_60_H_83_O_34_, 1347.4760) (Figure S9).

### Jasminumoside G (**2**)

White powder, [*α*]_D_^25^ − 173.7 (*c* 0.75, MeOH); UV [MeOH, nm (long *ε*)]: 235 (4.66); IR (KBr) *v*_max_: 3433, 1732, 1717, 1701, 1636, 1076 cm^–1^ (Figure S10); ^1^H (800 MHz, CD_3_OD) and ^13^C NMR (200 MHz, CD_3_OD): data shown in Table 2 (Figures S11 and S12); ^1^H–^1^H COSY, HMQC, HMBC, and pROESY spectra are provided in the Supporting Information (Figures S13–16); positive-ion ESI-MS *m/z*: 1357 [M + Na]^+^; HRESIMS *m/z*: 1357.4583 [M + Na]^+^ (calcd for C_59_H_82_O_34_Na, 1357.4580) (Figure S17); negative-ion ESI-MS *m/z*: 1333 [M – H]^–^; HRESIMS *m/z*: 1333.4590 [M – H]^–^ (calcd for C_59_H_81_O_34_, 1333.4604) (Figure S18).

### Jasminumoside H (**3**)

White powder, [*α*]_D_^25^ − 161.5 (*c* 0.72, MeOH); UV [MeOH, nm (long *ε*)]: 234 (4.47); IR (KBr) *v*_max_: 3416, 1734, 1717, 1701, 1636, 1076 cm^–1^ (Figure S19); ^1^H (800 MHz, CD_3_OD) and ^13^C NMR (200 MHz, CD_3_OD): data shown in Table 3 (Figures S20 and S21); ^1^H–^1^H COSY, HMQC, HMBC, and pROESY spectra are provided in the Supporting Information (Figures S22–S25); positive-ion ESI-MS *m/z*: 1357 [M + Na]^+^; HRESIMS *m/z*: 1357.4560 [M + Na]^+^ (calcd for C_59_H_82_O_34_Na, 1357.4580) (Figure S26); negative-ion ESI-MS *m/z*: 1333 [M – H]^–^; HRESIMS *m/z*: 1333.4624 [M – H]^–^ (calcd for C_59_H_81_O_34_, 1333.4604) (Figure S27).

### Jasminumoside I (**4**)

White powder, [*α*]_D_^25^ − 162.2 (*c* 0.50, MeOH); UV [MeOH, nm (long *ε*)]: 232 (4.29); IR (KBr) *v*_max_: 3327, 1734, 1717, 1699, 1636, 1076 cm^–1^ (Figure S28); ^1^H (800 MHz, CD_3_OD) and ^13^C NMR (200 MHz, CD_3_OD): data shown in Table 4 (Figures S29 and S30); ^1^H–^1^H COSY, HMQC, HMBC, and pROESY spectra are provided in the Supporting Information (Figures S31–S34); positive-ion ESI-MS *m/z*: 1357 [M + Na]^+^; HRESIMS *m/z*: 1357.4579 [M + Na]^+^ (calcd for C_59_H_82_O_34_Na, 1357.4580) (Figure S35); negative-ion ESI-MS *m/z*: 1333 [M – H]^–^; HRESIMS *m/z*: 1333.4617 [M – H]^–^ (calcd for C_59_H_81_O_34_, 1333.4604) (Figure S36).

### Jasminumoside J (**5**)

White powder, [*α*]_D_^25^ − 171.8 (*c* 0.67, MeOH); UV [MeOH, nm (long *ε*)]: 235 (4.53); IR (KBr) *v*_max_: 3424, 1734, 1717, 1701, 1636, 1076 cm^–1^ (Figure S37); ^1^H (800 MHz, CD_3_OD) and ^13^C NMR (200 MHz, CD_3_OD): data shown in Table 5 (Figures S38 and S39); ^1^H–^1^H COSY, HMQC, HMBC, and pROESY spectra are provided in the Supporting Information (Figures S40–S43); positive-ion ESI-MS *m/z*: 1341 [M + Na]^+^; HRESIMS *m/z*: 1341.4614 [M + Na]^+^ (calcd for C_59_H_82_O_33_Na, 1341.4631) (Figure S44); negative-ion ESI-MS *m/z*: 1317 [M – H]^–^; HRESIMS *m/z*: 1317.4674 [M – H]^–^ (calcd for C_59_H_81_O_33_, 1317.4655) (Figure S45).

### Jasminumoside K (**6**)

White powder, [*α*]_D_^25^ − 280.3 (*c* 1.10, MeOH); UV [MeOH, nm (long *ε*)]: 237 (4.47); IR (KBr) *v*_max_: 3422, 1736, 1713, 1632, 1082 cm^–1^ (Figure S46); ^1^H (800 MHz, CD_3_OD) and ^13^C NMR (200 MHz, CD_3_OD): data shown in Table 5 (Figures S47 and S48); ^1^H–^1^H COSY, HMQC, HMBC, and NOESY spectra are provided in the Supporting Information (Figures S49–S52); positive-ion ESI-MS *m/z*: 1369 [M + Na]^+^; HRESIMS *m/z*: 1369.4937 [M + Na]^+^ (calcd for C_61_H_86_O_33_Na, 1369.4944) (Figure S53); negative-ion ESI-MS *m/z*: 1345 [M – H]^–^; HRESIMS *m/z*: 1345.4973 [M – H]^–^ (calcd for C_61_H_85_O_33_, 1345.4968) (Figure S54).

### Acid hydrolysis of Jasminumosides F–K (**1**–**6**)

A solution of **1**–**6** (2.0 mg each) in 1 M HCl (1.0 mL) was stirred at 80 °C for 1 h. After cooling, the solution was neutralized with Amberlite IRA-400 (OH^−^ form) and the resin was removed by filtration. After removal of the solvent from the filtrate under reduced pressure, the resulting residue was partitioned in EtOAc–H_2_O (1:1, v/v). Both organic and aqueous phases were evaporated in vacuo. The aqueous layer was then subjected to HPLC analysis using the following conditions: HPLC column, Kaseisorb LC NH2-60-5, 4.6 mm i.d. × 250 mm (Tokyo Kasei Co., Ltd.); detection, optical rotation [Shodex OR-2 (Showa Denko Co., Ltd.)]; mobile phase, CH_3_CN–H_2_O (85:15, v/v); and flow rate, 0.8 mL/min. The identification of D-glucose [*t*_R_: 13.9 min (positive optical rotation)] in the aqueous phase was carried out by comparing its retention time and optical rotation with those of an authentic sample [21, 26, 27].

### Methylation of Jasminumosides F–H (**1**–**3**) or J (**5**) with TMSCHN_2_

A solution of **1** (1.0 mg) in MeOH (0.2 mL) was treated with trimethylsilyldiazomethane (TMSCHN_2_, ca. 10% in hexane, Tokyo Chemical Industry Co., Ltd., Tokyo, Japan, 0.1 mL) and the mixture was stirred at room temperature (25 °C) for 1 h. Removal of the solvent under reduced pressure afforded **8** (1.1 mg). In a similar manner, **2**, **3**, or **5** (1.0 mg) was converted to **9** (1.0 mg from **2**), **10** (1.1 mg from **3**), or **6** (1.0 mg, from **5**), respectively.

### Time-course treatment of sambacoside E (**8**) or Jasminumoside F (**1**) with Et_2_NH–MeOH

A solution of **8** (4.6 mg) in Et_2_NH–MeOH (1:200, v/v, 0.5 mL) was stirred at 60 °C. At 12 or 24 h after treatment, an aliquot of the reaction mixture was neutralized with Dowex HCR W2 (H^+^ form), and the resin was removed by filtration. After removal of the solvent under reduced pressure, a residue was obtained that was subjected to LC-MS analysis [Instruments: Thermo Fisher Scientific Ultimate + EvactivePlus, column: Cosmosil 5C_18_-MS-II (2.0 mm i.d. × 150 mm), HPLC detection: UV (230 nm), mobile phase: 0–5 min hold MeOH–H_2_O (25:75, v/v) → 40–50 min hold MeOH, flow rate: 0.2 mL/min, injection: 2 *µ*L, ionization mode: positive-ion ESI] to identify **22a** (*t*_R_: 15.17 min) [*t*_R_: 15.17 min, *m/z*: 441.1358 [M + Na]^–^ (calcd for C_18_H_26_O_11_Na, 441.1367)], **20** [*t*_R_: 16.40 min, *m/z*: 613.2460 [M + Na]^–^ (calcd for C_27_H_42_O_14_Na, 613.2467)], molihuasides E [**18**, *t*_R_: 22.54 min, *m/z*: 999.3654 [M + Na]^–^ (calcd for C_44_H_64_O_24_Na, 999.3680)], and D [**17**, *t*_R_: 22.81 min, *m/z*: 999.3661 [M + Na]^–^ (calcd for C_44_H_64_O_24_Na, 999.3680)] along with unreacted **8** (*t*_R_: 24.80 min). According to a similar procedure, **22b** [*t*_R_: 4.07 min, *m/z*: 427.1209 [M + Na]^–^ (calcd for C_17_H_24_O_11_Na, 427.1211)], **22a** (*t*_R_: 15.17 min), **20** (*t*_R_: 16.40 min), the mono-demethyl ester derivative of molihuaside E [**1a**, *t*_R_: 20.57 min, *m/z*: 985.3507 [M + Na]^–^ (calcd for C_43_H_62_O_24_Na, 985.3523)], and **17** (*t*_R_: 22.81 min) were identified along with unreacted **1** (*t*_R_: 23.00 min) upon LCMS analysis of the residue obtained upon treatment of **1** (2.0 mg) in Et_2_NH–MeOH (1:50, v/v, 1.0 mL) at 60 °C for 72 h. The residue was subsequently methylated as described above, and **18** (*t*_R_: 22.54 min) was identified instead of **1a**.

### Treatment of sambacoside A (**7**) or Jasminumoside G (**2**) with Et_2_NH–MeOH

A solution of **7** (5.0 mg) in diethylamine (Et_2_NH)–MeOH (1:200, v/v, 0.5 mL) was stirred at 60 °C for 24 h. After cooling, the reaction mixture was neutralized with Dowex HCR W2 (H+ form) and the resin was removed by filtration. After removal of the solvent under reduced pressure, a residue was obtained that was purified by HPLC [detection: UV (230 nm), column: Cosmosil 5C_18_-MS-II, MeOH–1% aqueous AcOH (40:60, v/v)] to give molihuaside A (0.3 mg) and **22a** (0.3 mg).

Using a similar procedure, a solution of **2** (2.3 mg) in Et_2_NH–MeOH (1:200, v/v, 0.5 mL) was stirred at 60 °C for 24 h. Each neutralized residue was subjected to LC-MS analysis (*vide ante*), leading to the identification fo the bisdemethyl ester derivatives of molihuaside A (**2a**) and **22a**. Each reaction residue was subsequently methylated in a similar manner as described above, and molihuaside A was identified instead of **2a** using LC-MS analysis.

## Supplementary Information

Below is the link to the electronic supplementary material.

Supplementary Material 1
