# Supplementary material for: Jasminumosides F–K, oligomeric secoiridoid glycosides from jasmin: the flowers of Jasminum sambac (L.) Aiton
Source: J Nat Med. 2026 Feb 24;80(3):827–43. doi: 10.1007/s11418-026-02018-5 (PMC13186831; doi:10.1007/s11418-026-02018-5)

Supplementary Information

Jasminumosides F–K, Oligomeric Secoiridoid Glycosides from Jasmin: the Flowers of *Jasminum sambac* (L.) Aiton

Naoki Inoue[1,3] · Yoshiaki Manse[1] · Toshio Morikawa[1,2]*

[1] Pharmaceutical Research and Technology Institute, Kindai University, 3-4-1 Kowakae, Higashi-osaka, Osaka 577-8502, Japan
[2] Antiaging Center, Kindai University, 3-4-1 Kowakae, Higashi-osaka, Osaka 577-8502, Japan
[3] Present address: School of Pharmacy and Pharmaceutical Sciences, Mukogawa Women's University, 11–68 Koshien Kyuban-cho, Nishinomiya, Hyogo 663–9942, Japan

* Correspondence: morikawa@kindai.ac.jp; Tel.: +81-6-4307-4306

Figure S1. IR spectrum of jasminumoside F (**1**) ········· S1
Figure S2. ^{1}H-NMR spectrum of **1** in CD_3OD ········· S2
Figure S3. ^{13}C NMR and DEPT135 spectra of **1** in CD_3OD ········· S3
Figure S4. DQF-COSY spectrum of **1** ········· S4
Figure S5. HSQC spectrum of **1** ········· S5
Figure S6. HMBC spectrum of **1** ········· S6
Figure S7. Phase-sensitive pROESY spectrum of **1** ········· S7
Figure S8. Positive-ion HRESIMS spectrum of **1** ········· S8
Figure S9. Negative-ion HRESIMS spectrum of **1** ········· S9
Figure S10. IR spectrum of jasminumoside G (**2**) ········· S10
Figure S11. ^{1}H-NMR spectrum of **2** in CD_3OD ········· S11
Figure S12. ^{13}C NMR and DEPT135 spectra of **2** in CD_3OD ········· S12
Figure S13. DQF-COSY spectrum of **2** ········· S13
Figure S14. HSQC spectrum of **2** ········· S14

Figure S15. HMBC spectrum of **2** ···· S15
Figure S16. Phase-sensitive pROESY spectrum of **2** ···· S16
Figure S17. Positive-ion HRESIMS spectrum of **2** ···· S17
Figure S18. Negative-ion HRESIMS spectrum of **2** ···· S18
Figure S19. IR spectrum of jasminumoside H (**3**) ···· S19
Figure S20. ^{1}H-NMR spectrum of **3** in CD_3OD ···· S20
Figure S21. ^{13}C NMR and DEPT135 spectra of **3** in CD_3OD ···· S21
Figure S22. DQF-COSY spectrum of **3** ···· S22
Figure S23. HSQC spectrum of **3** ···· S23
Figure S24. HMBC spectrum of **3** ···· S24
Figure S25. Phase-sensitive pROESY spectrum of **3** ···· S25
Figure S26. Positive-ion HRESIMS spectrum of **3** ···· S26
Figure S27. Negative-ion HRESIMS spectrum of **3** ···· S27
Figure S28. IR spectrum of jasminumoside I (**4**) ···· S28
Figure S29. ^{1}H-NMR spectrum of **4** in CD_3OD ···· S29
Figure S30. ^{13}C NMR and DEPT135 spectra of **4** in CD_3OD ···· S30
Figure S31. DQF-COSY spectrum of **4** ···· S31
Figure S32. HSQC spectrum of **4** ···· S32
Figure S33. HMBC spectrum of **4** ···· S33
Figure S34. Phase-sensitive pROESY spectrum of **4** ···· S34
Figure S35. Positive-ion HRESIMS spectrum of **4** ···· S35
Figure S36. Negative-ion HRESIMS spectrum of **4** ···· S36
Figure S37. IR spectrum of jasminumoside J (**5**) ···· S37
Figure S38. ^{1}H-NMR spectrum of **5** in CD_3OD ···· S38
Figure S39. ^{13}C NMR and DEPT135 spectra of **5** in CD_3OD ···· S39
Figure S40. DQF-COSY spectrum of **5** ···· S40
Figure S41. HSQC spectrum of **5** ···· S41

Figure S42. HMBC spectrum of **5** ···· S42
Figure S43. Phase-sensitive pROESY spectrum of **5** ···· S43
Figure S44. Positive-ion HRESIMS spectrum of **5** ···· S44
Figure S45. Negative-ion HRESIMS spectrum of **5** ···· S45
Figure S46. IR spectrum of jasminumoside K (**6**) ···· S46
Figure S47. ^{1}H-NMR spectrum of **6** in CD_3OD ···· S47
Figure S48. ^{13}C NMR and DEPT135 spectra of **6** in CD_3OD ···· S48
Figure S49. DQF-COSY spectrum of **6** ···· S49
Figure S50. HSQC spectrum of **6** ···· S50
Figure S51. HMBC spectrum of **6** ···· S51
Figure S52. Phase-sensitive pNOESY spectrum of **6** ···· S52
Figure S53. Positive-ion HRESIMS spectrum of **6** ···· S53
Figure S54. Negative-ion HRESIMS spectrum of **6** ···· S54

jasminumoside F (1)

SHIMADZU

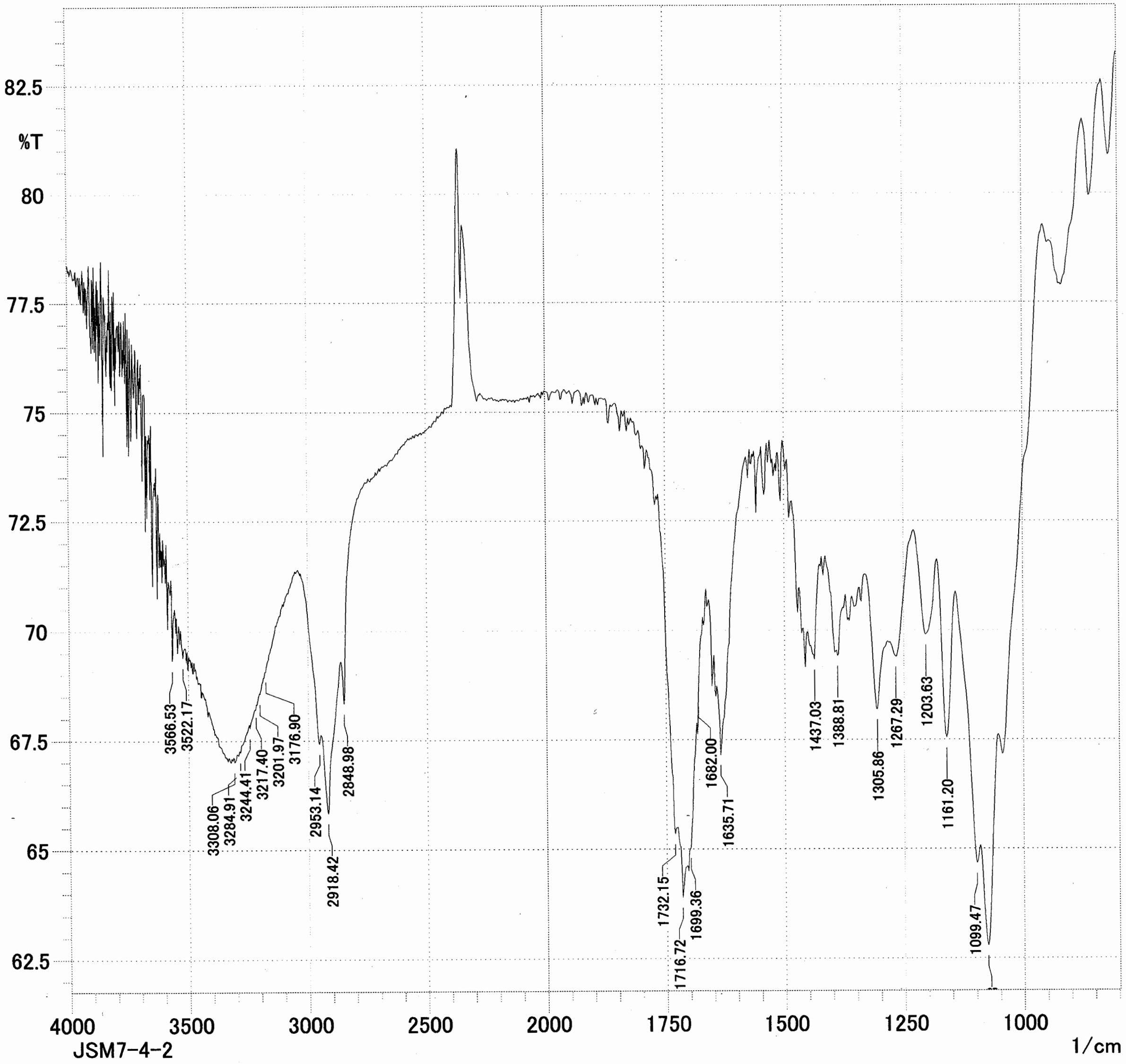

	ピーク	高さ	補正高さ	ベース (H)	ベース (L)	面積	補正面積
1	1076.33	62.791	3.303	1091.76	1053.18	7.292	0.415
2	1099.47	64.675	1.318	1139.98	1091.76	8.254	0.121
3	1161.2	67.551	3.705	1180.49	1141.91	6.125	0.443
4	1203.63	69.9	1.979	1228.71	1182.41	6.949	0.315
5	1267.29	69.403	0.937	1278.86	1230.64	7.29	0.134
6	1305.86	68.198	2.255	1330.94	1284.65	7.296	0.264
7	1388.81	69.426	0.236	1390.74	1373.38	2.669	0.007
8	1437.03	69.338	1.491	1450.53	1427.39	3.581	0.105
9	1635.71	67.153	1.952	1641.49	1581.7	8.999	0.229
10	1682	67.641	0.442	1683.93	1672.36	1.825	0
11	1699.36	65.004	0.368	1701.29	1687.79	2.405	0.013
12	1716.72	63.908	1.095	1726.36	1709	3.293	0.045
13	1732.15	65.485	0.802	1764.94	1728.29	5.956	0.075
14	2848.98	68.342	1.649	2862.49	2789.19	10.784	0.109
15	2918.42	65.837	2.336	2945.43	2864.41	13.855	0.527
16	2953.14	67.426	0.474	3026.44	2947.36	12.545	0.047
17	3176.9	69.087	0.046	3178.83	3134.46	6.983	0.01
18	3201.97	68.571	0.041	3203.9	3180.75	3.758	0.004
19	3217.4	68.208	0.051	3219.33	3205.83	2.227	0.001
20	3244.41	67.791	0.104	3246.34	3223.19	3.873	0.004
21	3284.91	67.238	0.058	3286.84	3248.27	6.577	0.01
22	3308.06	67.01	0.109	3309.99	3298.42	2.007	0.006
23	3522.17	69.397	0.361	3529.88	3514.45	2.431	0.016
24	3566.53	69.338	1.464	3568.46	3556.89	1.791	0.044

コメント;
JSM7-4-2

日 時; 2019/07/08 16:49:31
積 算; 5
分 解; 4 [1/cm]
ｱﾎﾟﾀﾞｲｾﾞｰｼｮﾝ; Happp-Genzel
分析者;Administrator

jasminumoside F (1)

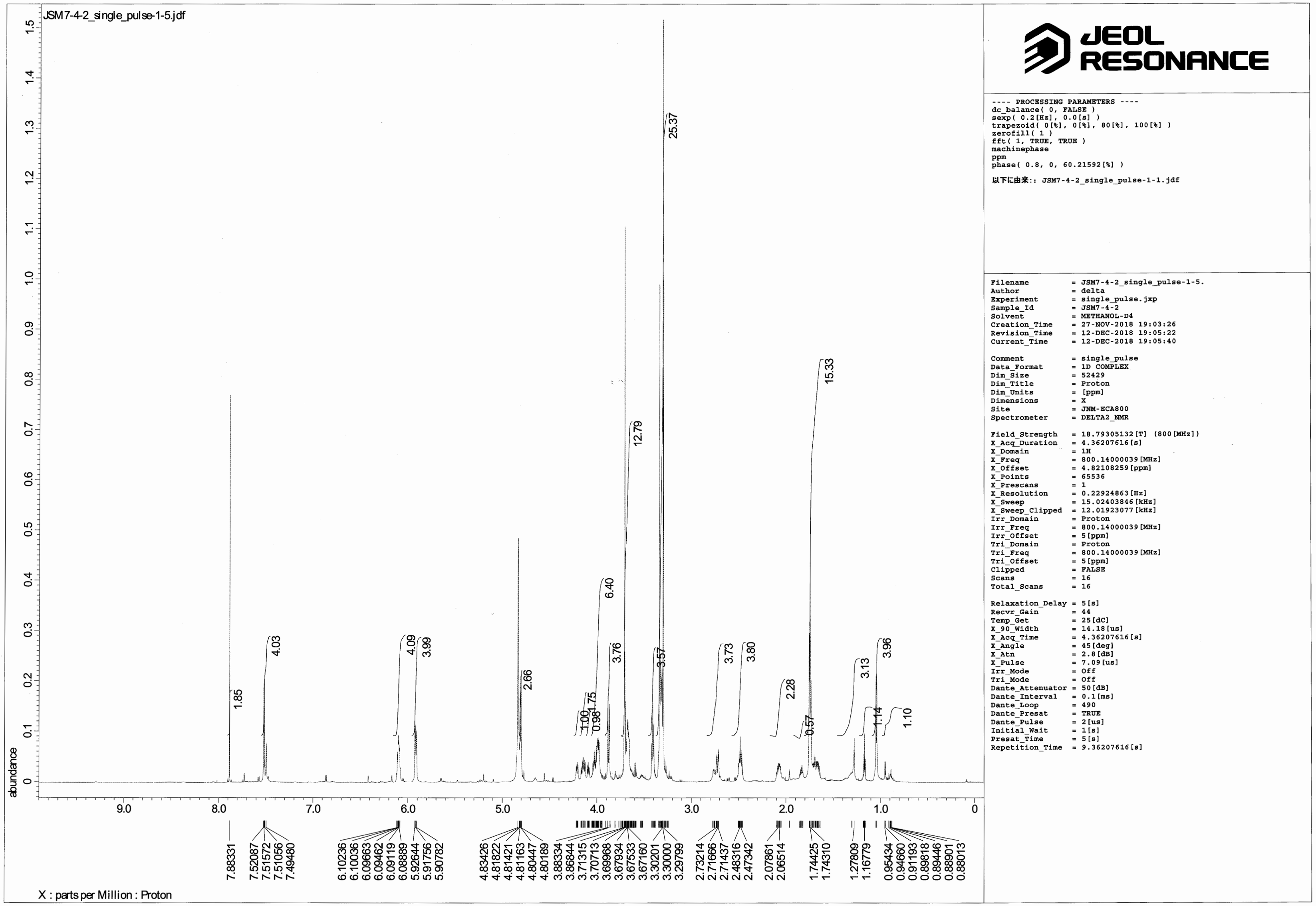

jasminumoside F (1)

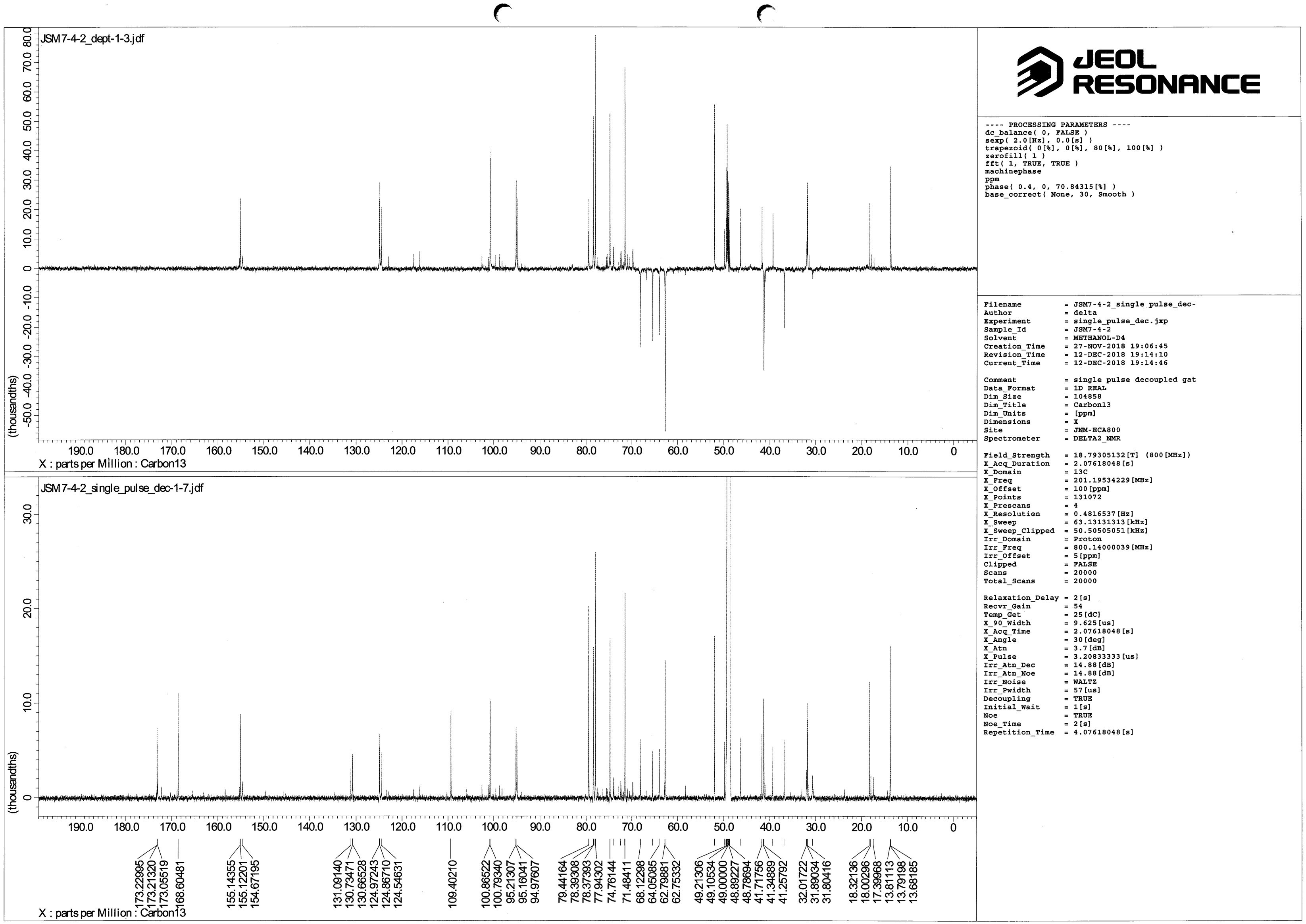

jasminumoside F (1)

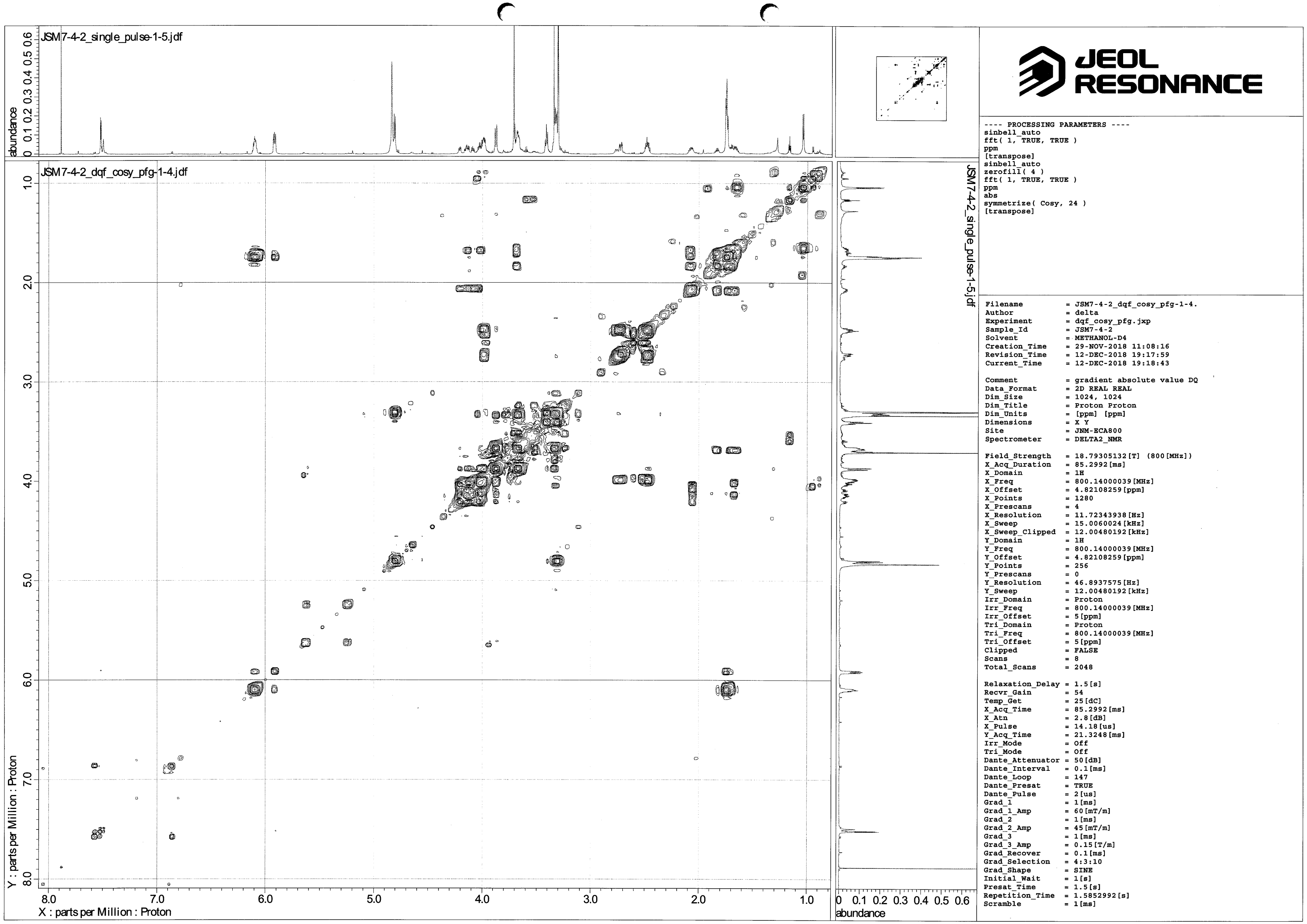

jasminumoside F (1)

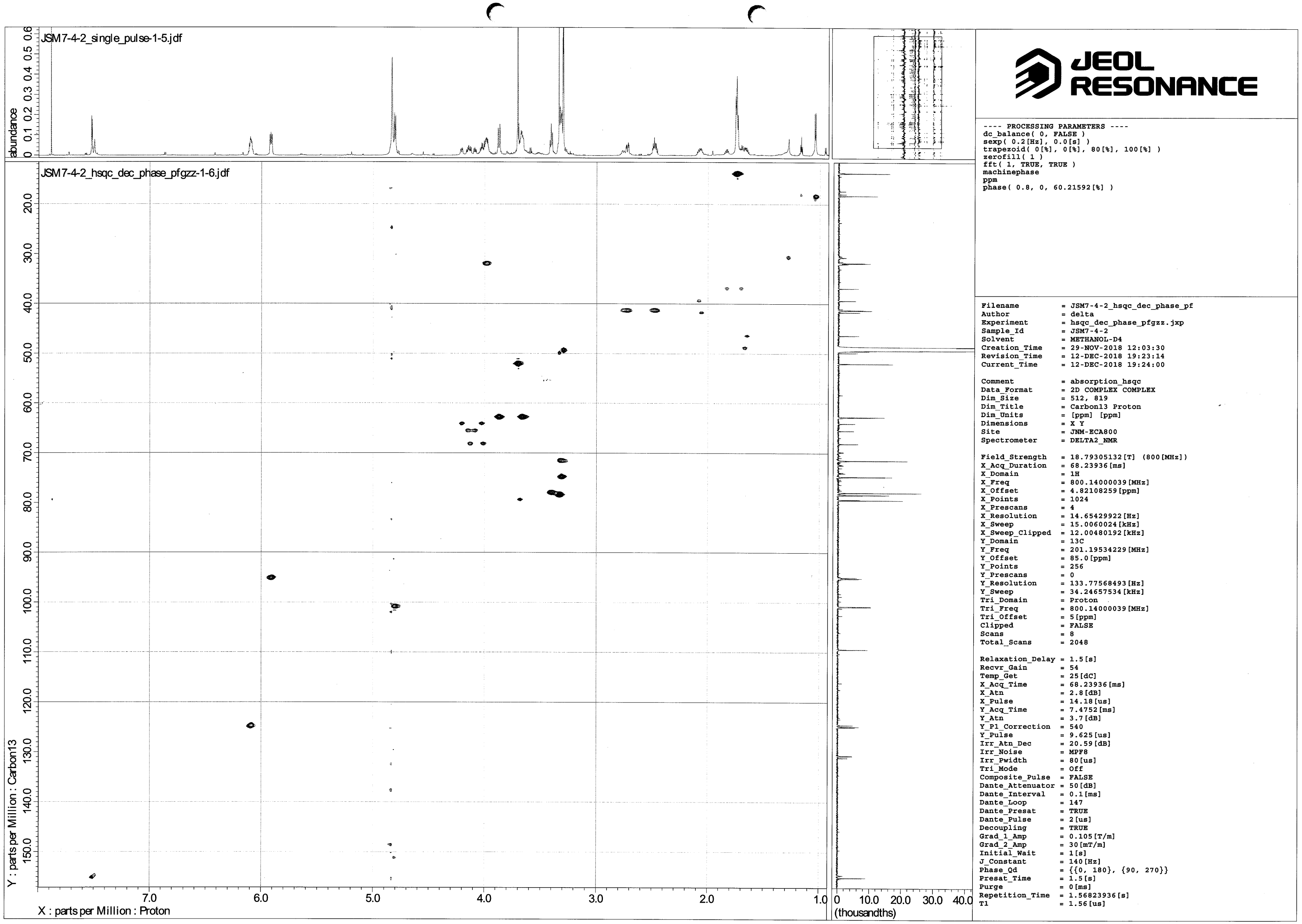

jasminumoside F (1)

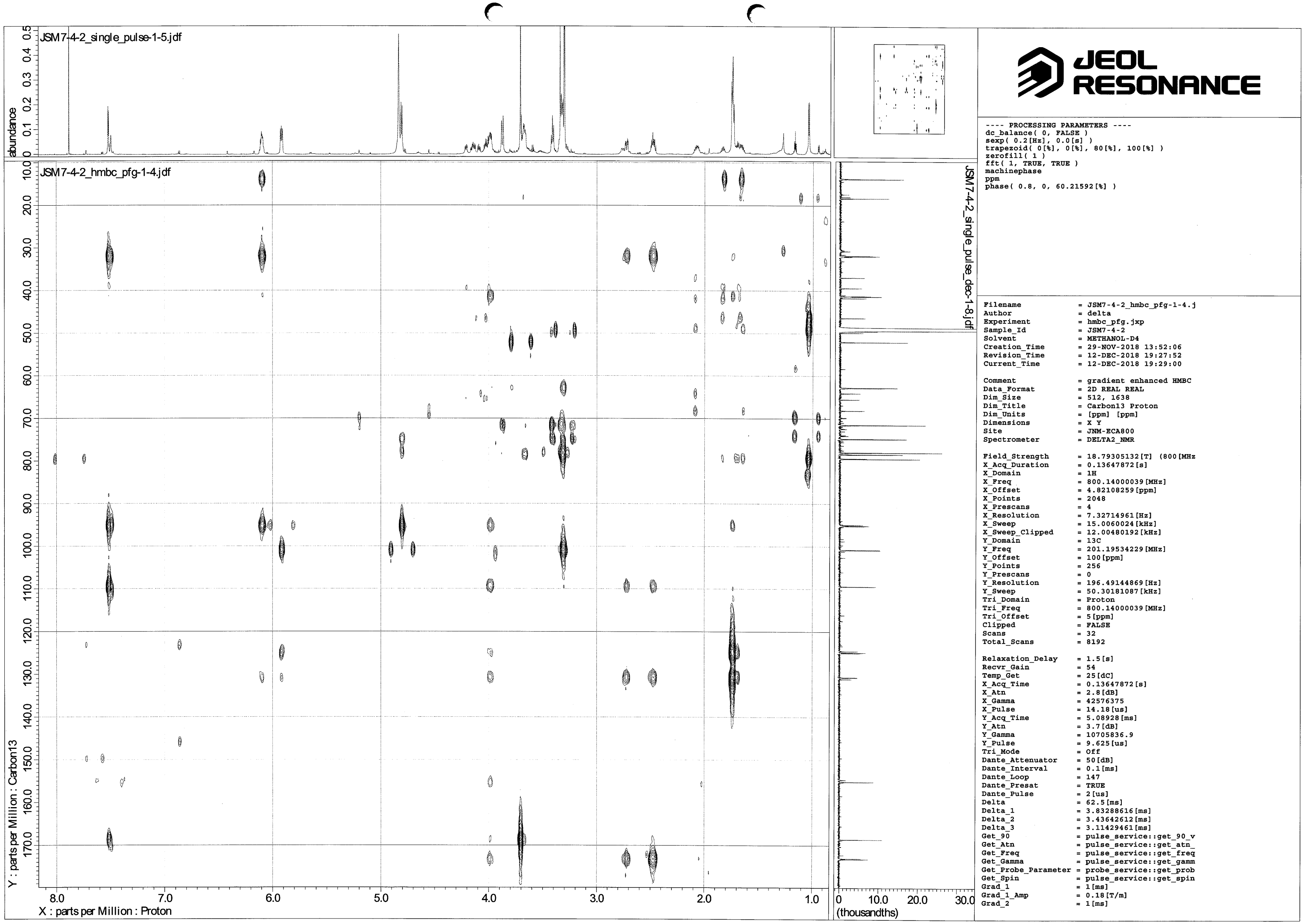

jasminumoside F (1)

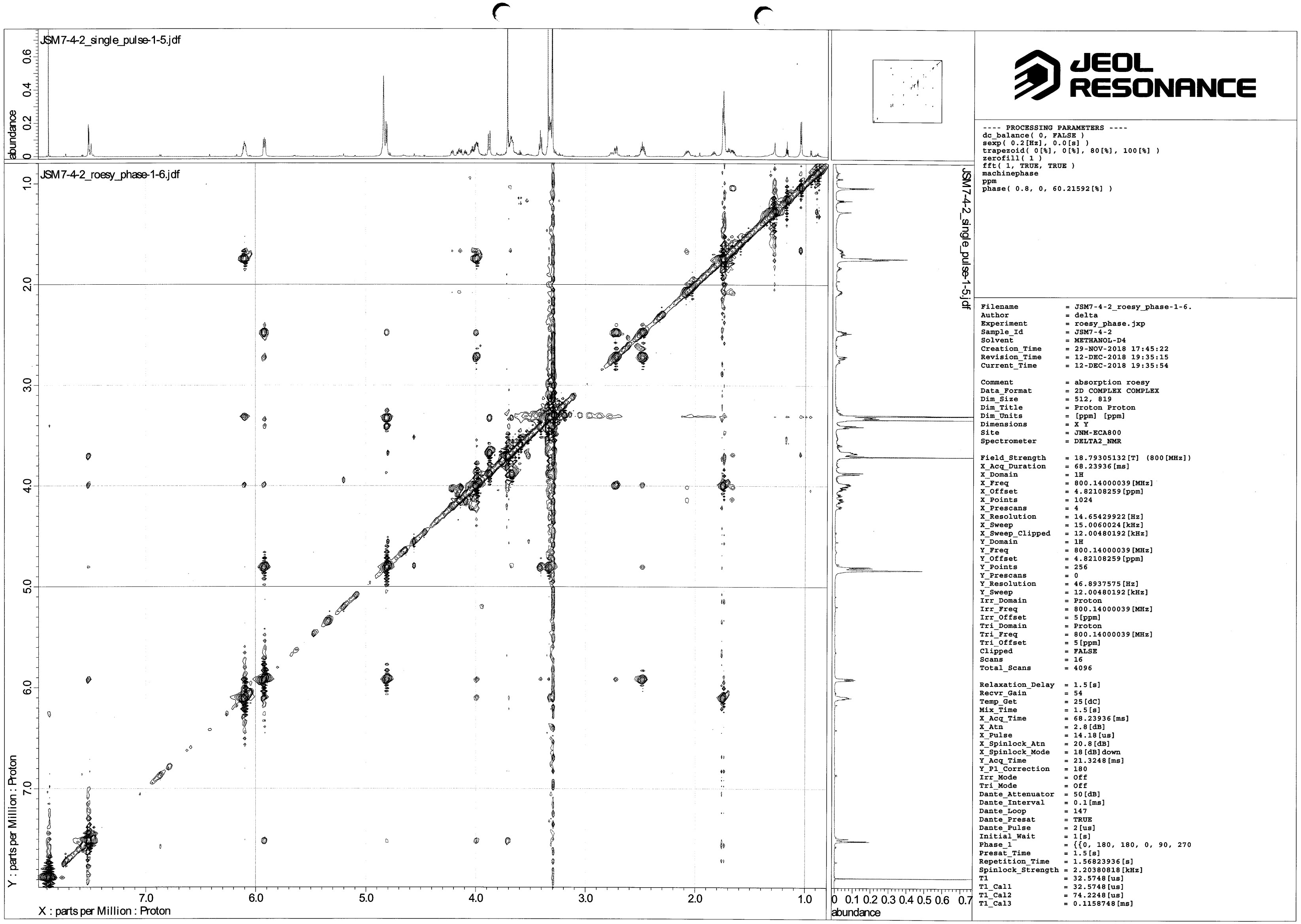

jasminumoside F (1)

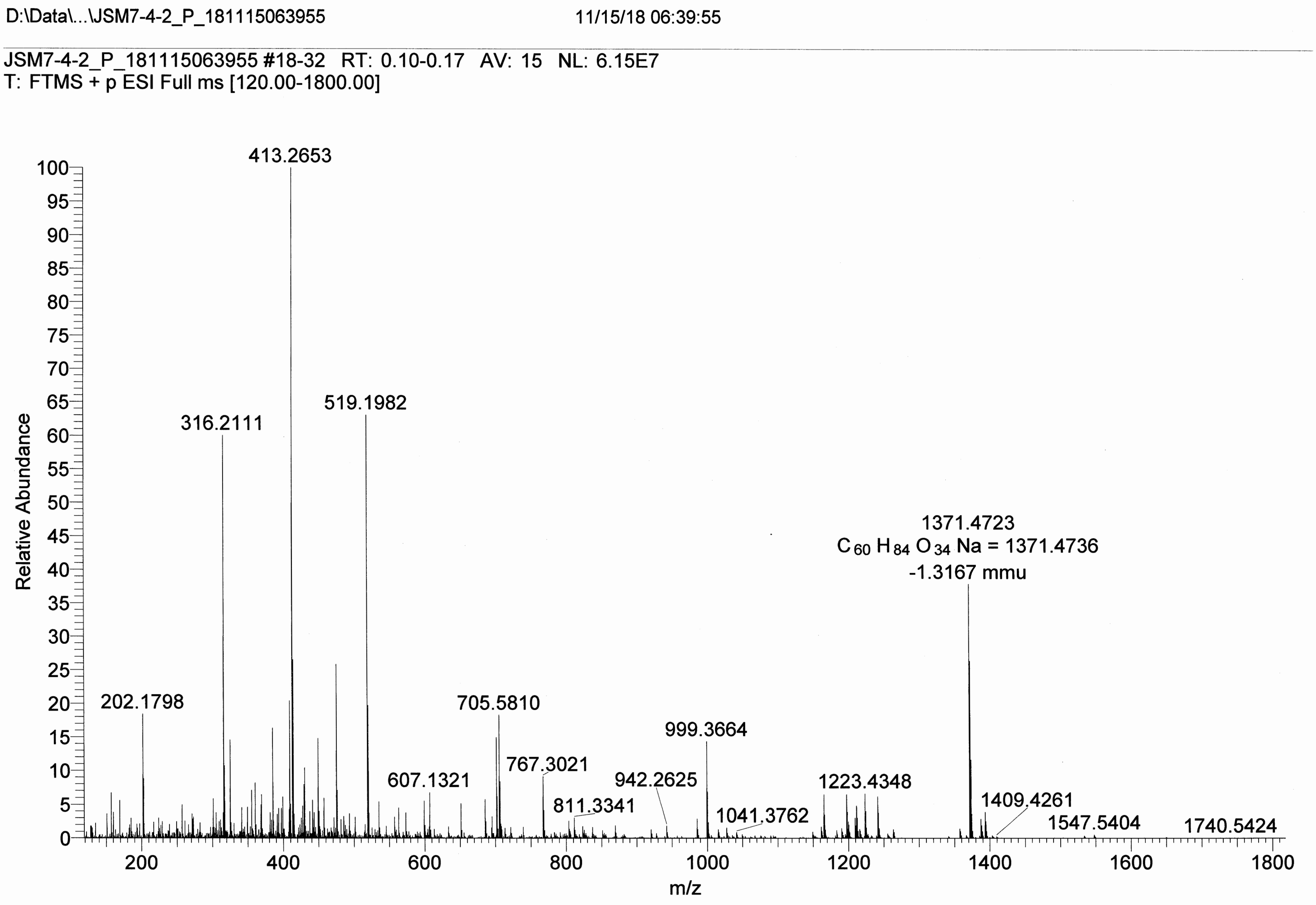

jasminumoside F (1)

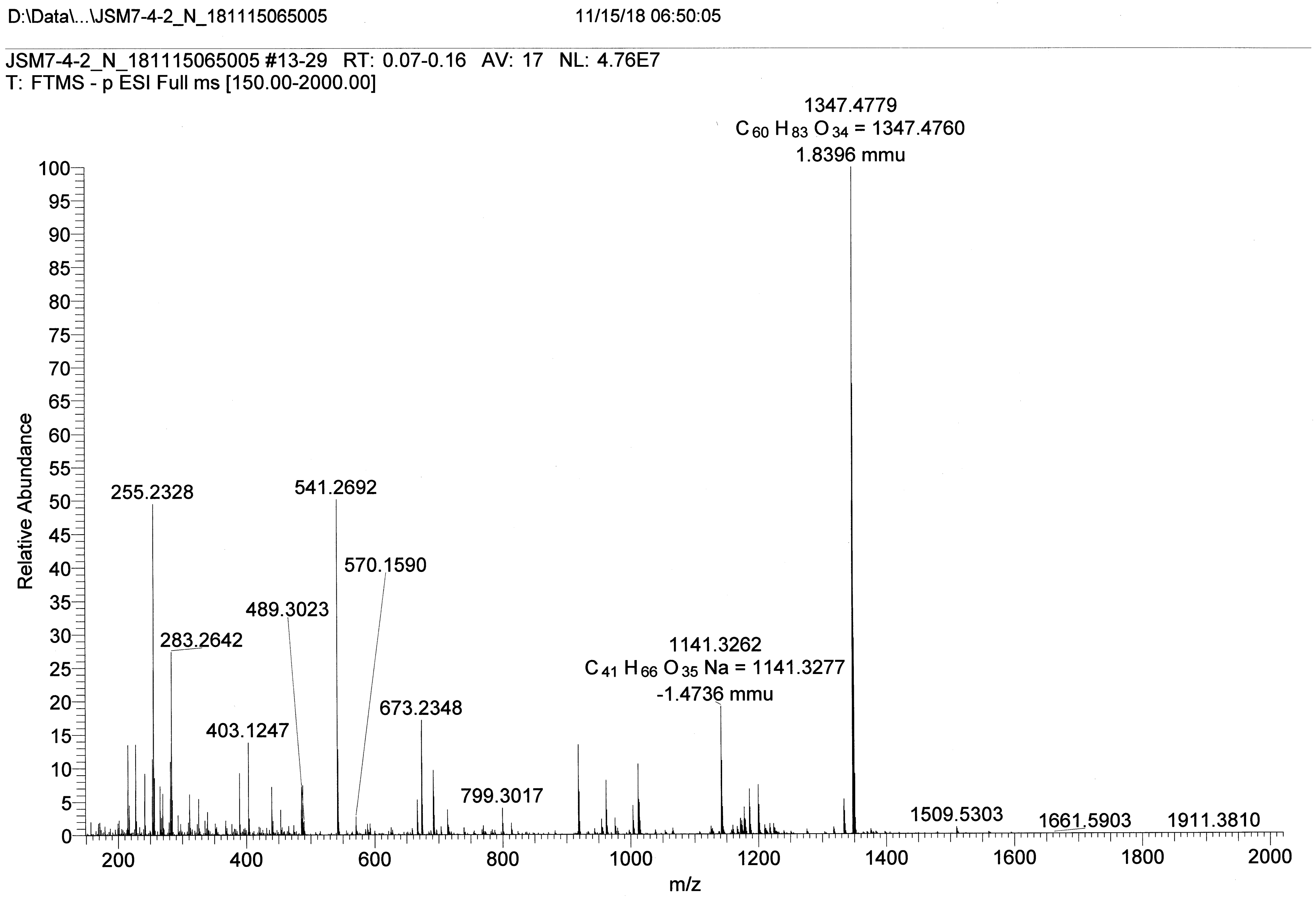

jasminumoside G (2)

SHIMADZU

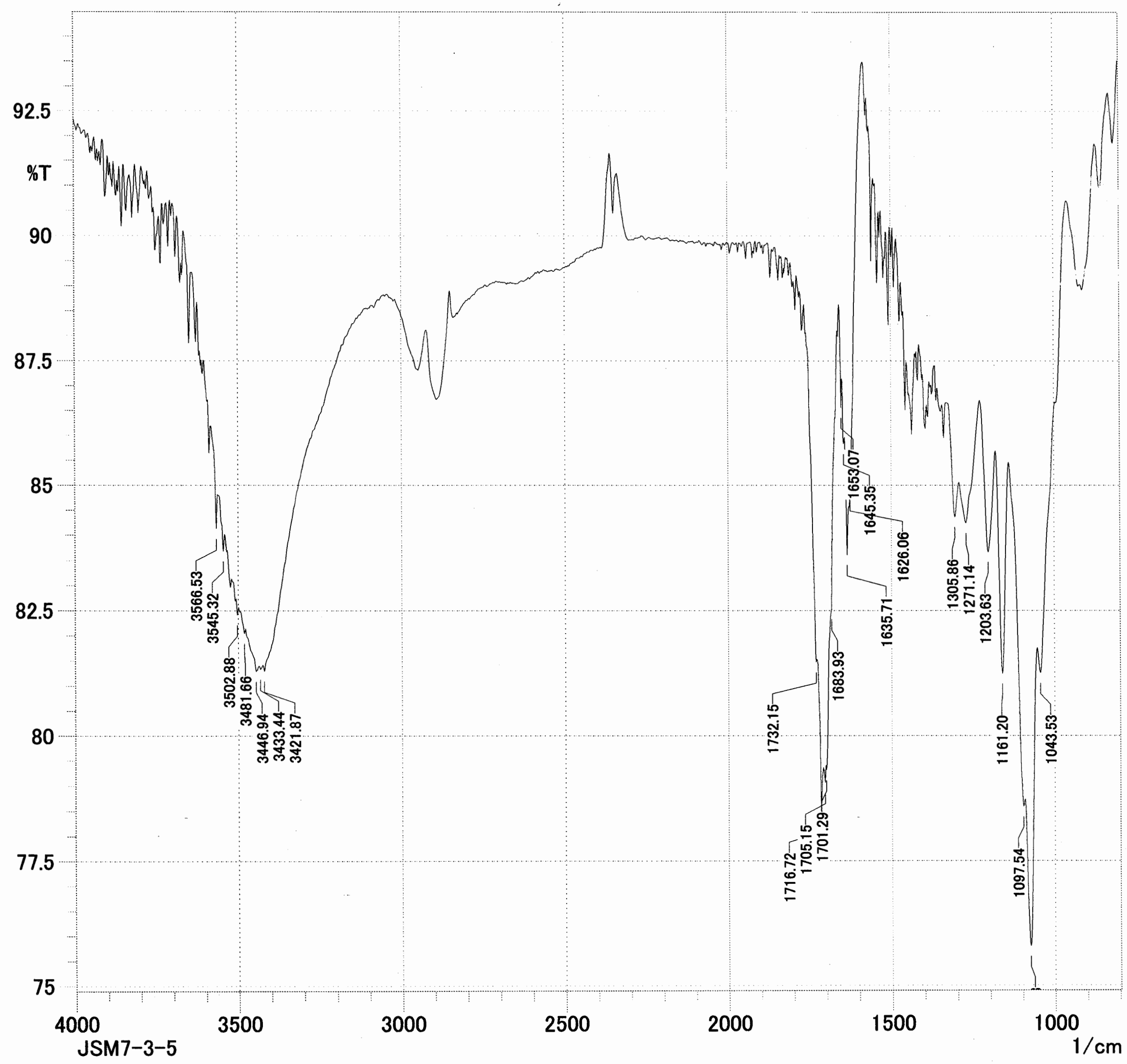

	ピーク	高さ	補正高さ	ベース (H)	ベース (L)	面積	補正面積
1	1043.53	81.263	1.344	1053.18	997.24	4.324	0.168
2	1076.33	75.79	4.197	1091.76	1055.11	3.942	0.447
3	1097.54	78.59	0.946	1139.98	1091.76	4.127	-0.023
4	1161.2	81.245	4.307	1180.49	1141.91	3.051	0.435
5	1203.63	83.671	2.464	1228.71	1182.41	3.296	0.3
6	1271.14	84.245	1.373	1292.36	1230.64	4.322	0.244
7	1305.86	84.366	1.174	1330.94	1294.29	2.513	0.083
8	1626.06	84.567	0.387	1627.99	1583.63	2.051	0.004
9	1635.71	83.615	1.556	1643.42	1629.92	0.977	0.042
10	1645.35	85.832	0.425	1651.14	1643.42	0.492	0.008
11	1653.07	86.561	0.95	1658.85	1651.14	0.441	0.008
12	1683.93	82.45	0.441	1685.86	1658.85	1.733	0.003
13	1701.29	79.318	0.455	1703.22	1687.79	1.427	0.023
14	1705.15	79.063	0.329	1710.93	1703.22	0.78	0.006
15	1716.72	78.506	1.573	1728.29	1710.93	1.7	0.049
16	1732.15	81.476	0.49	1759.16	1730.22	2.048	-0.034
17	3421.87	81.304	0.139	3423.8	3180.75	17.246	0.039
18	3433.44	81.34	0.065	3437.3	3425.72	1.036	0.002
19	3446.94	81.293	0.246	3477.8	3439.23	3.399	0.022
20	3481.66	82.047	0.179	3493.24	3477.8	1.311	0.007
21	3502.88	82.414	0.236	3506.74	3499.02	0.643	0.005
22	3545.32	83.691	0.477	3560.75	3541.46	1.439	0.016
23	3566.53	84.128	0.964	3581.96	3562.68	1.337	0.021

コメント;
JSM7-3-5

日 時; 2019/12/23 17:17:19
積 算; 20
分 解; 4 [1/cm]
アポダイゼーション; Happ-Genzel
分析者;Administrator

jasminumoside G (2)

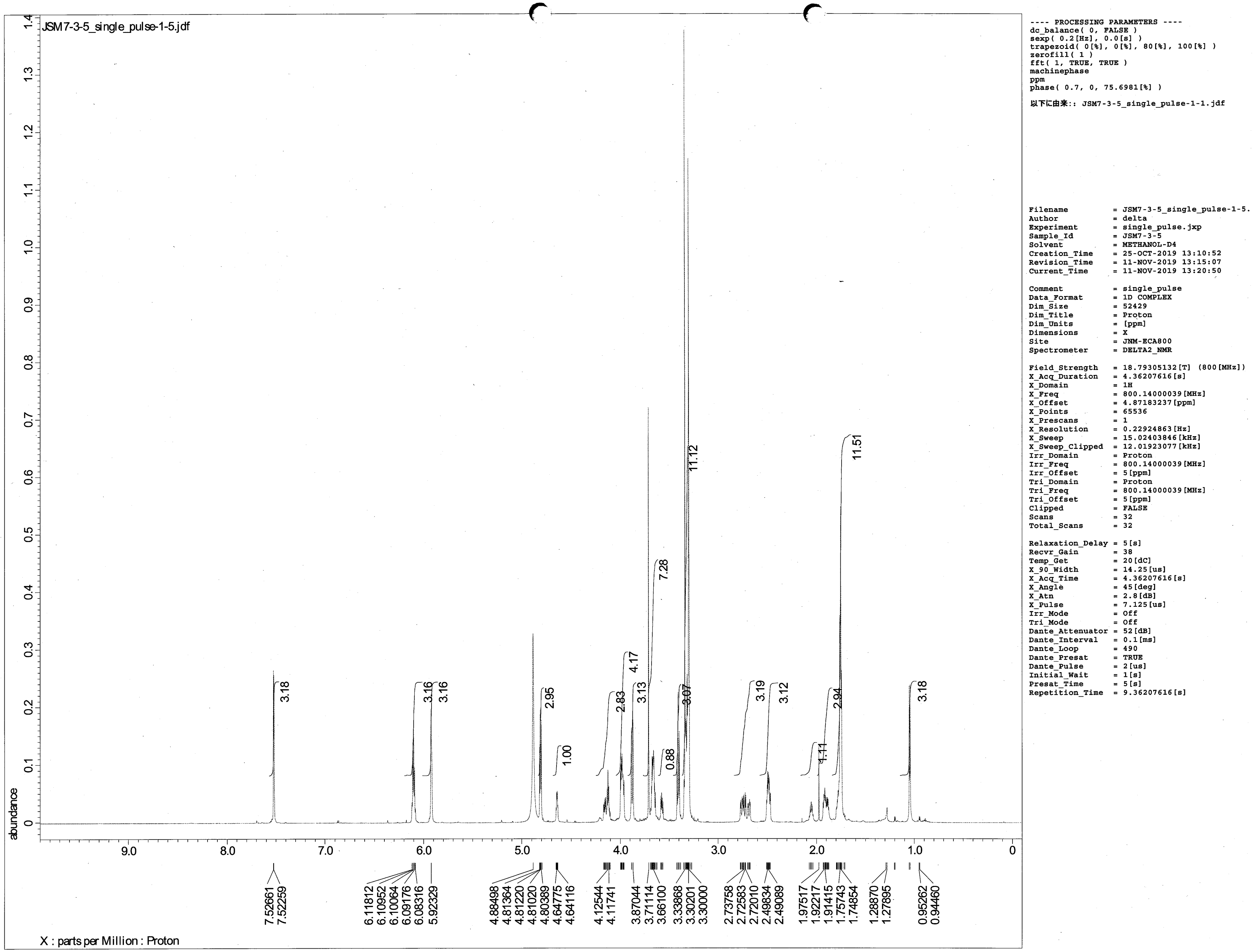


```
---- PROCESSING PARAMETERS ----
dc_balance( 0, FALSE )
sexp( 0.2[Hz], 0.0[s] )
trapezoid( 0[%], 0[%], 80[%], 100[%] )
zerofill( 1 )
fft( 1, TRUE, TRUE )
machinephase
ppm
phase( 0.7, 0, 75.6981[%] )

以下に由来:: JSM7-3-5_single_pulse-1-1.jdf

Filename          = JSM7-3-5_single_pulse-1-5.
Author            = delta
Experiment        = single_pulse.jxp
Sample_Id         = JSM7-3-5
Solvent           = METHANOL-D4
Creation_Time     = 25-OCT-2019 13:10:52
Revision_Time     = 11-NOV-2019 13:15:07
Current_Time      = 11-NOV-2019 13:20:50

Comment           = single_pulse
Data_Format       = 1D COMPLEX
Dim_Size          = 52429
Dim_Title         = Proton
Dim_Units         = [ppm]
Dimensions        = X
Site              = JNM-ECA800
Spectrometer      = DELTA2_NMR

Field_Strength    = 18.79305132[T] (800[MHz])
X_Acq_Duration    = 4.36207616[s]
X_Domain          = 1H
X_Freq            = 800.14000039[MHz]
X_Offset          = 4.87183237[ppm]
X_Points          = 65536
X_Prescans        = 1
X_Resolution      = 0.22924863[Hz]
X_Sweep           = 15.02403846[kHz]
X_Sweep_Clipped   = 12.01923077[kHz]
Irr_Domain        = Proton
Irr_Freq          = 800.14000039[MHz]
Irr_Offset        = 5[ppm]
Tri_Domain        = Proton
Tri_Freq          = 800.14000039[MHz]
Tri_Offset        = 5[ppm]
Clipped           = FALSE
Scans             = 32
Total_Scans       = 32

Relaxation_Delay  = 5[s]
Recvr_Gain        = 38
Temp_Get          = 20[dC]
X_90_Width        = 14.25[us]
X_Acq_Time        = 4.36207616[s]
X_Angle           = 45[deg]
X_Atn             = 2.8[dB]
X_Pulse           = 7.125[us]
Irr_Mode          = Off
Tri_Mode          = Off
Dante_Attenuator  = 52[dB]
Dante_Interval    = 0.1[ms]
Dante_Loop        = 490
Dante_Presat      = TRUE
Dante_Pulse       = 2[us]
Initial_Wait      = 1[s]
Presat_Time       = 5[s]
Repetition_Time   = 9.36207616[s]
```

jasminumoside G (2)

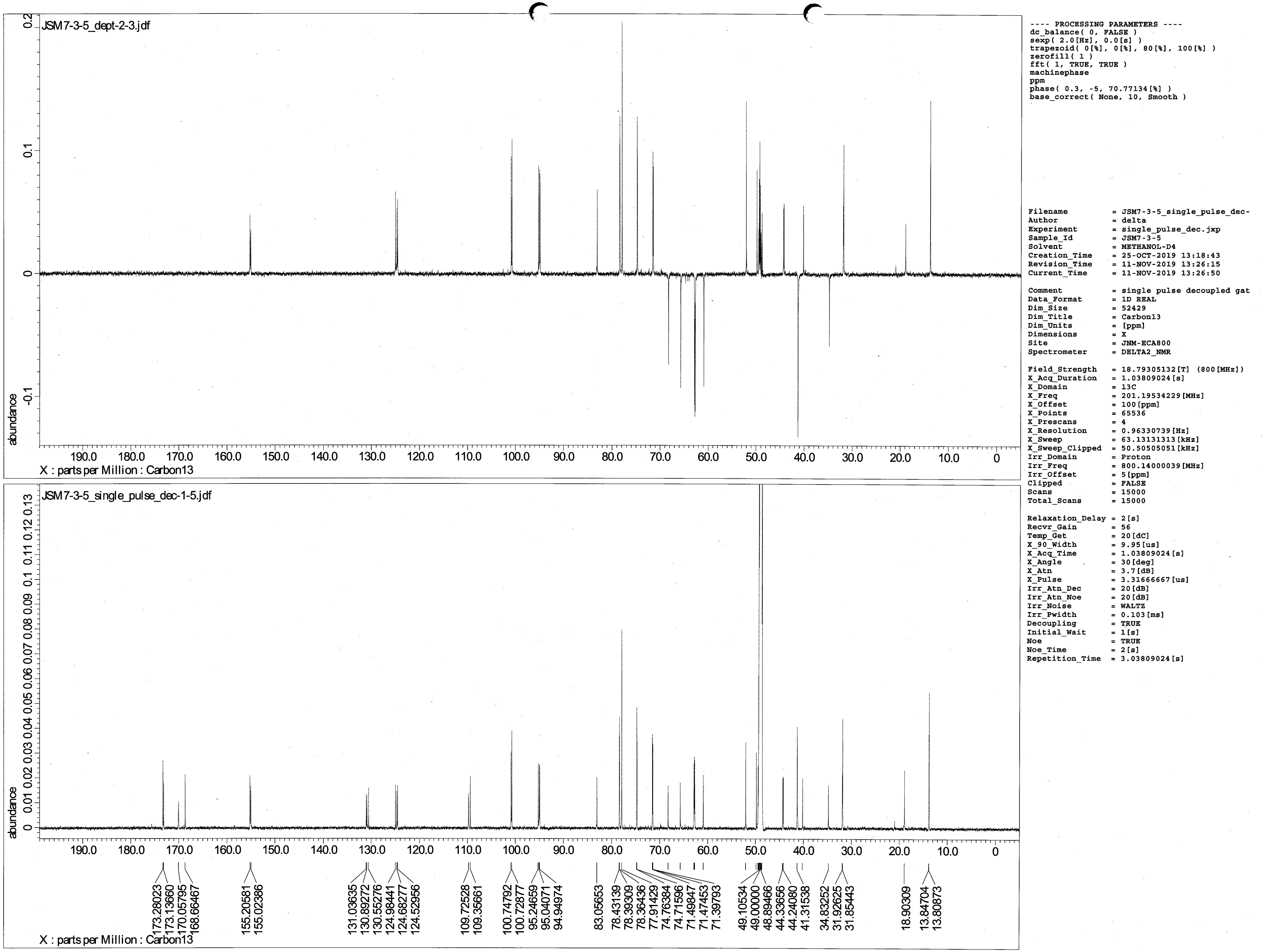

jasminumoside G (2)

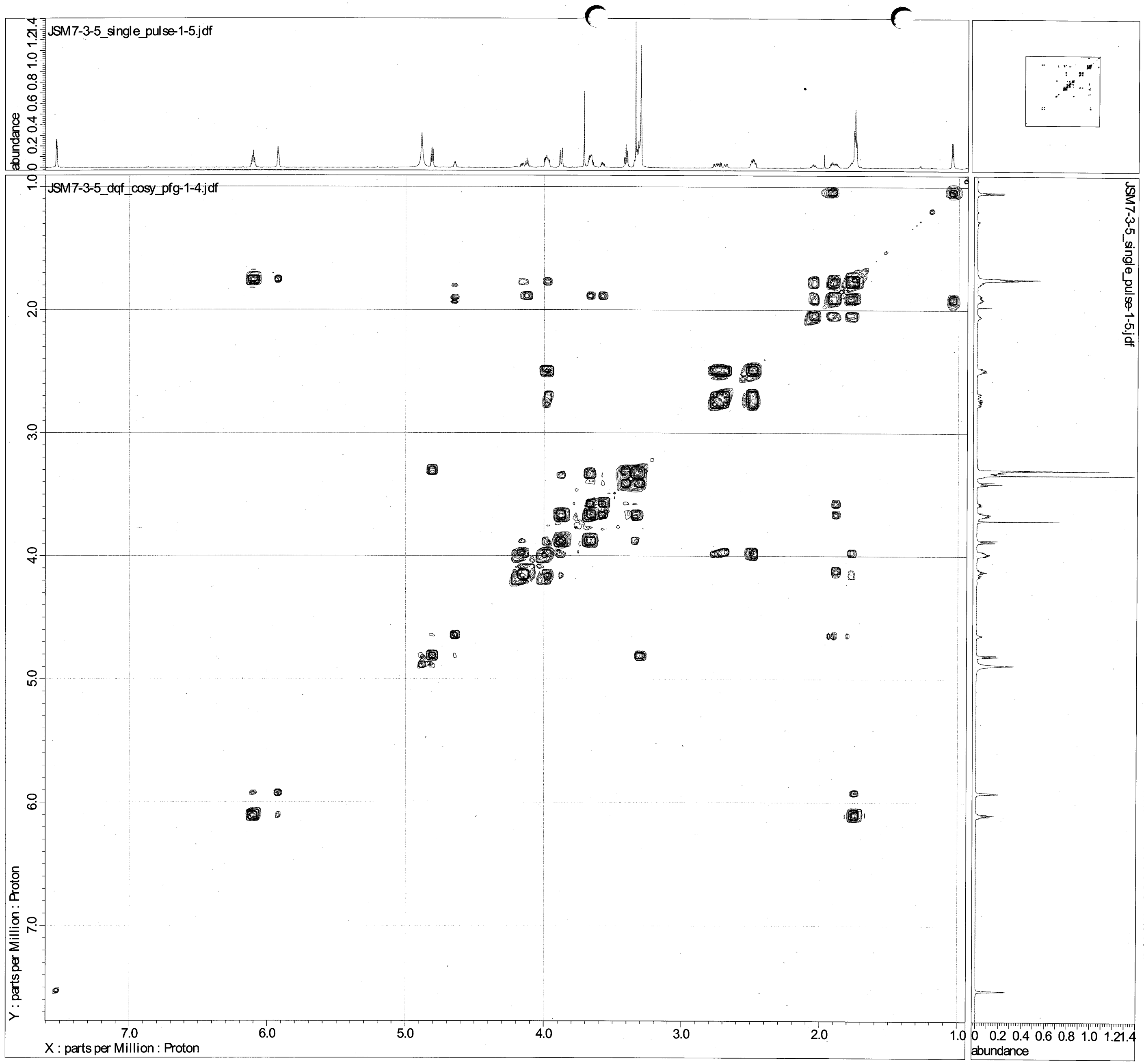


```
---- PROCESSING PARAMETERS ----
sinbell_auto
fft( 1, TRUE, TRUE )
ppm
[transpose]
sinbell_auto
zerofill( 4 )
fft( 1, TRUE, TRUE )
ppm
abs
symmetrize( Cosy, 24 )
[transpose]
```

```
Filename            = JSM7-3-5_dqf_cosy_pfg-1-4.
Author              = delta
Experiment          = dqf_cosy_pfg.jxp
Sample_Id           = JSM7-3-5
Solvent             = METHANOL-D4
Creation_Time       = 26-OCT-2019 04:33:56
Revision_Time       = 11-NOV-2019 18:40:35
Current_Time        = 11-NOV-2019 18:41:37

Comment             = gradient absolute value DQ
Data_Format         = 2D REAL REAL
Dim_Size            = 1024, 1024
Dim_Title           = Proton Proton
Dim_Units           = [ppm] [ppm]
Dimensions          = X Y
Site                = JNM-ECA800
Spectrometer        = DELTA2_NMR

Field_Strength      = 18.79305132[T] (800[MHz])
X_Acq_Duration      = 85.2992[ms]
X_Domain            = 1H
X_Freq              = 800.14000039[MHz]
X_Offset            = 4.83926439[ppm]
X_Points            = 1280
X_Prescans          = 4
X_Resolution        = 11.72343938[Hz]
X_Sweep             = 15.0060024[kHz]
X_Sweep_Clipped     = 12.00480192[kHz]
Y_Domain            = 1H
Y_Freq              = 800.14000039[MHz]
Y_Offset            = 4.83926439[ppm]
Y_Points            = 256
Y_Prescans          = 0
Y_Resolution        = 46.8937575[Hz]
Y_Sweep             = 12.00480192[kHz]
Irr_Domain          = Proton
Irr_Freq            = 800.14000039[MHz]
Irr_Offset          = 5[ppm]
Tri_Domain          = Proton
Tri_Freq            = 800.14000039[MHz]
Tri_Offset          = 5[ppm]
Clipped             = FALSE
Scans               = 8
Total_Scans         = 2048

Relaxation_Delay    = 1.5[s]
Recvr_Gain          = 38
Temp_Get            = 20[dC]
X_Acq_Time          = 85.2992[ms]
X_Atn               = 2.8[dB]
X_Pulse             = 14.25[us]
Y_Acq_Time          = 21.3248[ms]
Irr_Mode            = Off
Tri_Mode            = Off
Dante_Attenuator    = 52[dB]
Dante_Interval      = 0.1[ms]
Dante_Loop          = 147
Dante_Presat        = TRUE
Dante_Pulse         = 2[us]
Grad_1              = 1[ms]
Grad_1_Amp          = 60[mT/m]
Grad_2              = 1[ms]
Grad_2_Amp          = 45[mT/m]
Grad_3              = 1[ms]
Grad_3_Amp          = 0.15[T/m]
Grad_Recover        = 0.1[ms]
Grad_Selection      = 4:3:10
Grad_Shape          = SINE
Initial_Wait        = 1[s]
Presat_Time         = 1.5[s]
Repetition_Time     = 1.5852992[s]
Scramble            = 1[ms]
T1                  = 1[us]
Zf_Factor           = 4
```

jasminumoside G (2)

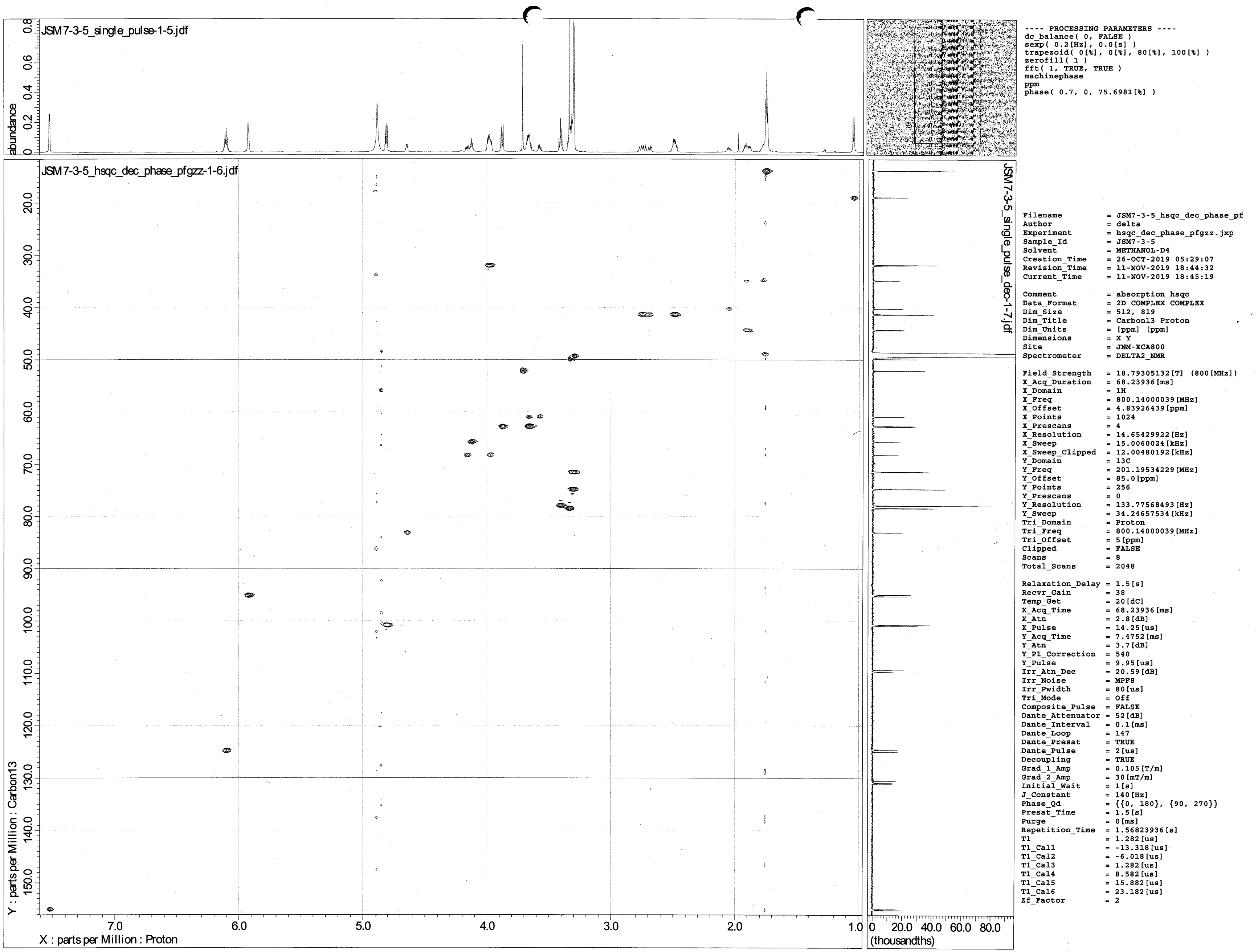

jasminumoside G (2)

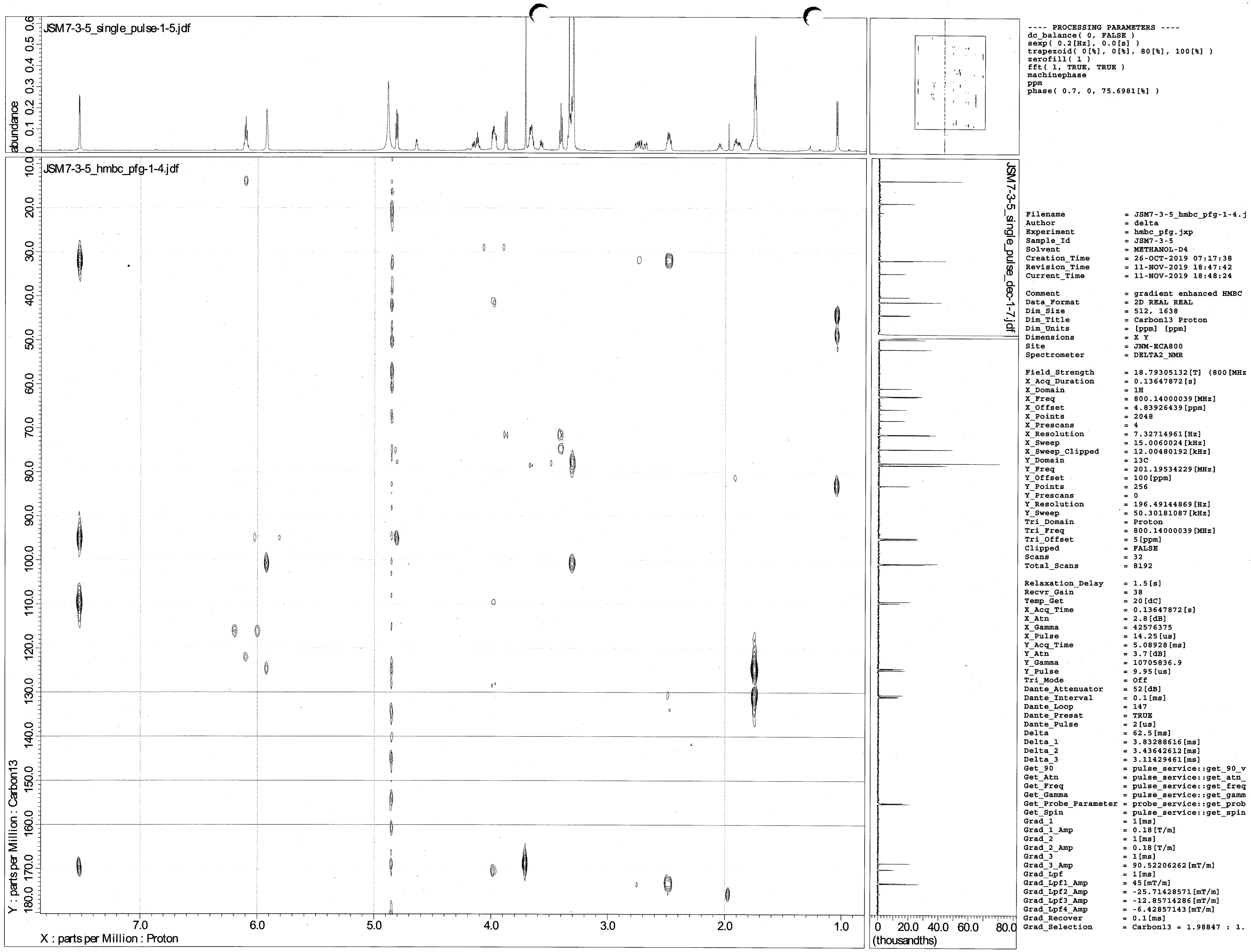


```
---- PROCESSING PARAMETERS ----
dc_balance( 0, FALSE )
sexp( 0.2[Hz], 0.0[s] )
trapezoid( 0[%], 0[%], 80[%], 100[%] )
zerofill( 1 )
fft( 1, TRUE, TRUE )
machinephase
ppm
phase( 0.7, 0, 75.6981[%] )

Filename              = JSM7-3-5_hmbc_pfg-1-4.j
Author                = delta
Experiment            = hmbc_pfg.jxp
Sample_Id             = JSM7-3-5
Solvent               = METHANOL-D4
Creation_Time         = 26-OCT-2019 07:17:38
Revision_Time         = 11-NOV-2019 18:47:42
Current_Time          = 11-NOV-2019 18:48:24

Comment               = gradient enhanced HMBC
Data_Format           = 2D REAL REAL
Dim_Size              = 512, 1638
Dim_Title             = Carbon13 Proton
Dim_Units             = [ppm] [ppm]
Dimensions            = X Y
Site                  = JNM-ECA800
Spectrometer          = DELTA2_NMR

Field_Strength        = 18.79305132[T] (800[MHz
X_Acq_Duration        = 0.13647872[s]
X_Domain              = 1H
X_Freq                = 800.14000039[MHz]
X_Offset              = 4.83926439[ppm]
X_Points              = 2048
X_Prescans            = 4
X_Resolution          = 7.32714961[Hz]
X_Sweep               = 15.0060024[kHz]
X_Sweep_Clipped       = 12.00480192[kHz]
Y_Domain              = 13C
Y_Freq                = 201.19534229[MHz]
Y_Offset              = 100[ppm]
Y_Points              = 256
Y_Prescans            = 0
Y_Resolution          = 196.49144869[Hz]
Y_Sweep               = 50.30181087[kHz]
Tri_Domain            = Proton
Tri_Freq              = 800.14000039[MHz]
Tri_Offset            = 5[ppm]
Clipped               = FALSE
Scans                 = 32
Total_Scans           = 8192

Relaxation_Delay      = 1.5[s]
Recvr_Gain            = 38
Temp_Get              = 20[dC]
X_Acq_Time            = 0.13647872[s]
X_Atn                 = 2.8[dB]
X_Gamma               = 42576375
X_Pulse               = 14.25[us]
Y_Acq_Time            = 5.08928[ms]
Y_Atn                 = 3.7[dB]
Y_Gamma               = 10705836.9
Y_Pulse               = 9.95[us]
Tri_Mode              = Off
Dante_Attenuator      = 52[dB]
Dante_Interval        = 0.1[ms]
Dante_Loop            = 147
Dante_Presat          = TRUE
Dante_Pulse           = 2[us]
Delta                 = 62.5[ms]
Delta_1               = 3.83288616[ms]
Delta_2               = 3.43642612[ms]
Delta_3               = 3.11429461[ms]
Get_90                = pulse_service::get_90_v
Get_Atn               = pulse_service::get_atn_
Get_Freq              = pulse_service::get_freq
Get_Gamma             = pulse_service::get_gamm
Get_Probe_Parameter   = probe_service::get_prob
Get_Spin              = pulse_service::get_spin
Grad_1                = 1[ms]
Grad_1_Amp            = 0.18[T/m]
Grad_2                = 1[ms]
Grad_2_Amp            = 0.18[T/m]
Grad_3                = 1[ms]
Grad_3_Amp            = 90.52206262[mT/m]
Grad_Lpf              = 1[ms]
Grad_Lpf1_Amp         = 45[mT/m]
Grad_Lpf2_Amp         = -25.71428571[mT/m]
Grad_Lpf3_Amp         = -12.85714286[mT/m]
Grad_Lpf4_Amp         = -6.42857143[mT/m]
Grad_Recover          = 0.1[ms]
Grad_Selection        = Carbon13 = 1.98847 : 1.
```

jasminumoside G (2)

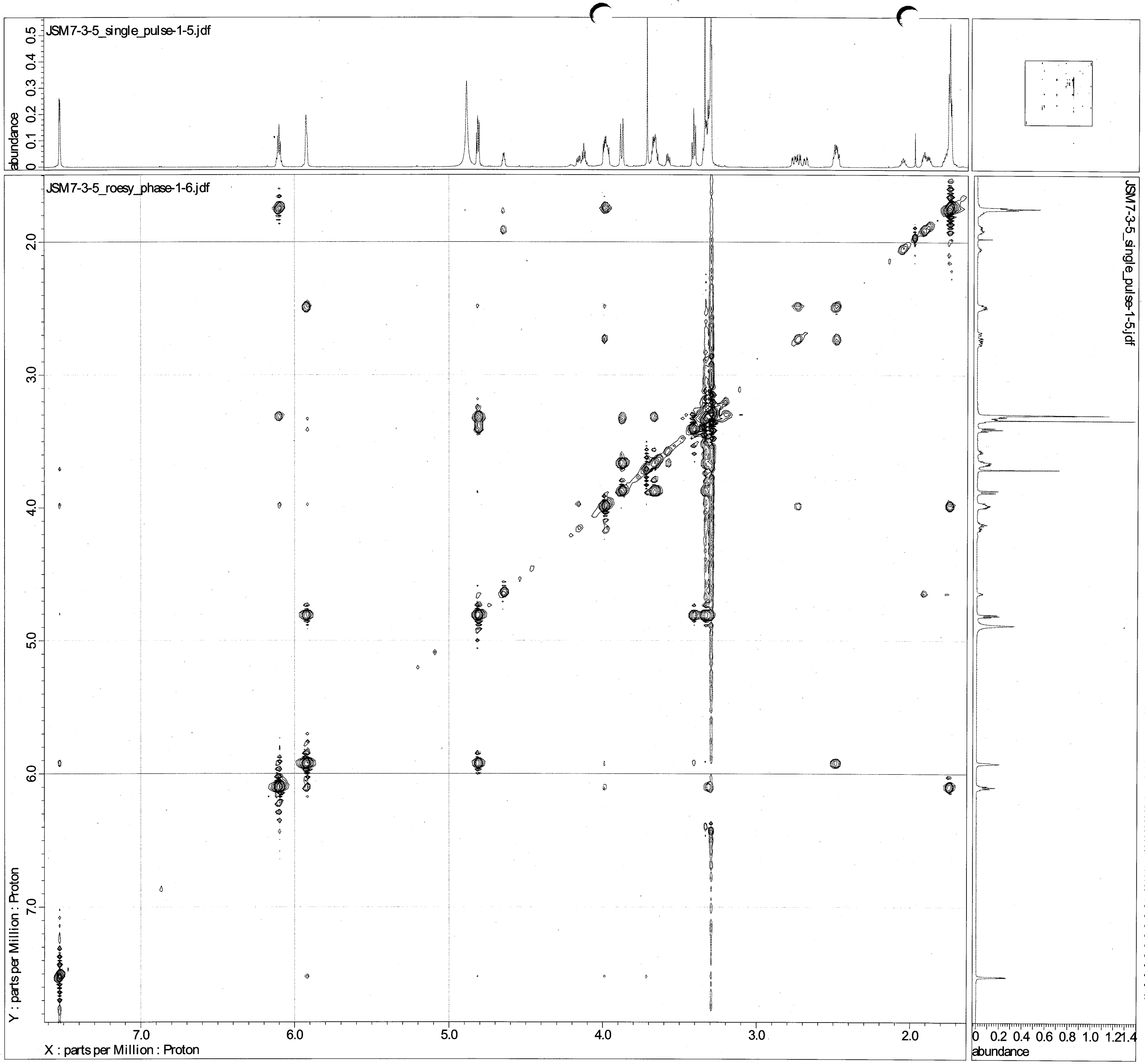


```
---- PROCESSING PARAMETERS ----
sexp( 5.0[Hz], 0.0[s] )
trapezoid( 0[%], 0[%], 80[%], 100[%] )
zerofill( 1 )
fft( 1, TRUE, TRUE )
ppm
machinephase
phase( -9, -35, 32.39609[%] )
[transpose]
sexp( 5.0[Hz], 0.0[s] )
trapezoid( 0[%], 0[%], 80[%], 100[%] )
zerofill( 2 )
fft( 1, TRUE, TRUE )
ppm
machinephase
[transpose]

Filename            = JSM7-3-5_roesy_phase-1-6.
Author              = delta
Experiment          = roesy_phase.jxp
Sample_Id           = JSM7-3-5
Solvent             = METHANOL-D4
Creation_Time       = 26-OCT-2019 11:10:51
Revision_Time       = 11-NOV-2019 18:51:58
Current_Time        = 11-NOV-2019 18:52:38

Comment             = absorption roesy
Data_Format         = 2D COMPLEX COMPLEX
Dim_Size            = 512, 819
Dim_Title           = Proton Proton
Dim_Units           = [ppm] [ppm]
Dimensions          = X Y
Site                = JNM-ECA800
Spectrometer        = DELTA2_NMR

Field_Strength      = 18.79305132[T] (800[MHz])
X_Acq_Duration      = 68.23936[ms]
X_Domain            = 1H
X_Freq              = 800.14000039[MHz]
X_Offset            = 4.83926439[ppm]
X_Points            = 1024
X_Prescans          = 4
X_Resolution        = 14.65429922[Hz]
X_Sweep             = 15.0060024[kHz]
X_Sweep_Clipped     = 12.00480192[kHz]
Y_Domain            = 1H
Y_Freq              = 800.14000039[MHz]
Y_Offset            = 4.83926439[ppm]
Y_Points            = 256
Y_Prescans          = 0
Y_Resolution        = 46.8937575[Hz]
Y_Sweep             = 12.00480192[kHz]
Irr_Domain          = Proton
Irr_Freq            = 800.14000039[MHz]
Irr_Offset          = 5[ppm]
Tri_Domain          = Proton
Tri_Freq            = 800.14000039[MHz]
Tri_Offset          = 5[ppm]
Clipped             = FALSE
Scans               = 16
Total_Scans         = 4096

Relaxation_Delay    = 1.5[s]
Recvr_Gain          = 38
Temp_Get            = 20[dC]
Mix_Time            = 1.5[s]
X_Acq_Time          = 68.23936[ms]
X_Atn               = 2.8[dB]
X_Pulse             = 14.25[us]
X_Spinlock_Atn      = 20.8[dB]
X_Spinlock_Mode     = 18[dB]down
Y_Acq_Time          = 21.3248[ms]
Y_P1_Correction     = 180
Irr_Mode            = Off
Tri_Mode            = Off
Dante_Attenuator    = 52[dB]
Dante_Interval      = 0.1[ms]
Dante_Loop          = 147
Dante_Presat        = TRUE
Dante_Pulse         = 2[us]
Initial_Wait        = 1[s]
Phase_1             = {{0, 180, 180, 0, 90, 270
Presat_Time         = 1.5[s]
Repetition_Time     = 1.56823936[s]
Spinlock_Strength   = 2.19298246[kHz]
T1                  = 32.53[us]
T1_Cal1             = 32.53[us]
T1_Cal2             = 74.18[us]
T1_Cal3             = 0.11583[ms]
T1_Cal4             = 0.15748[ms]
T1_Cal5             = 0.19913[ms]
T1_Cal6             = 0.24078[ms]
Temp1               = 18[dB]
Temp2               = 8
Zf_Factor           = 3
```

jasminumoside G (2)

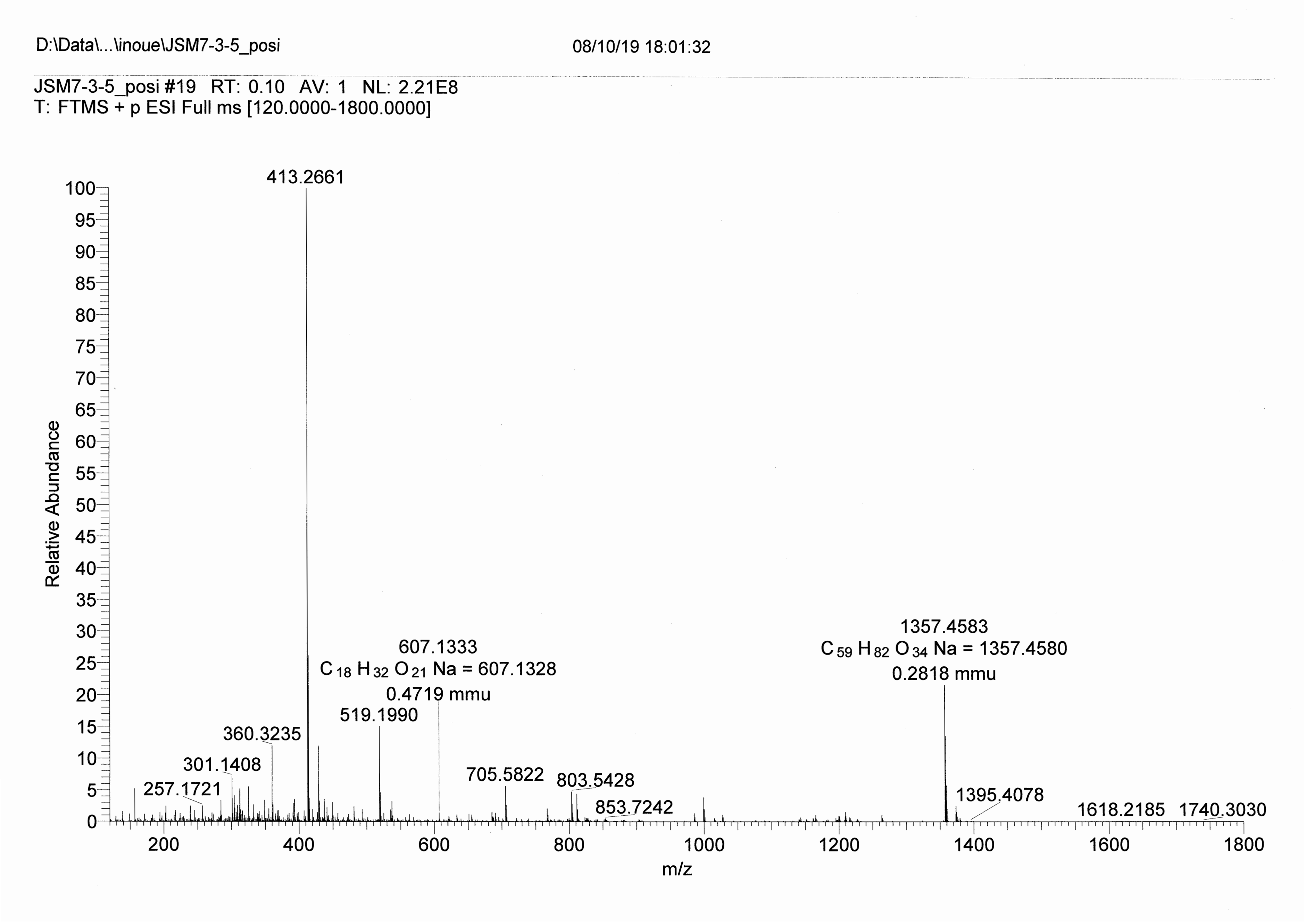

jasminumoside G (2)

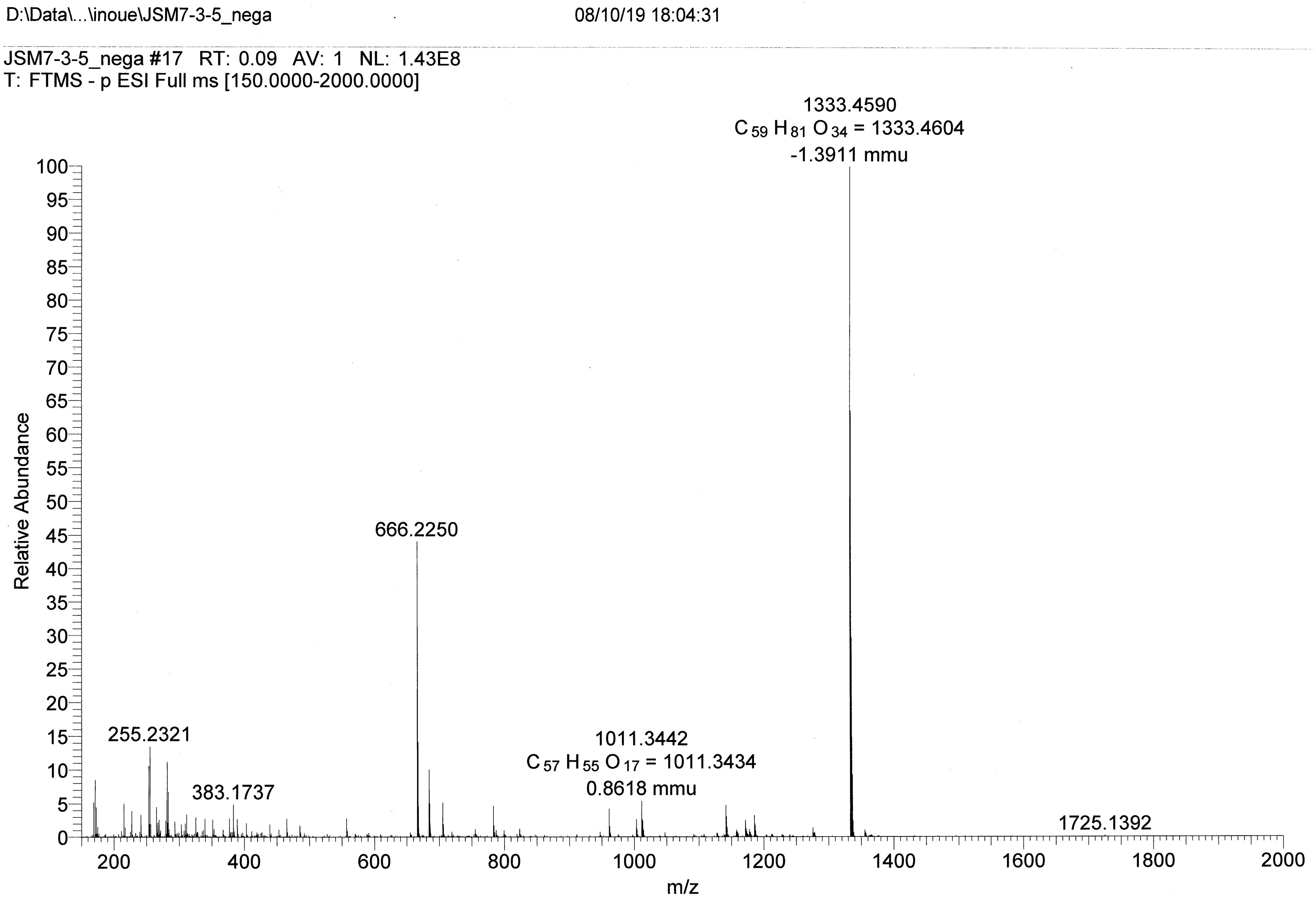

jasminumoside H (3)

SHIMADZU

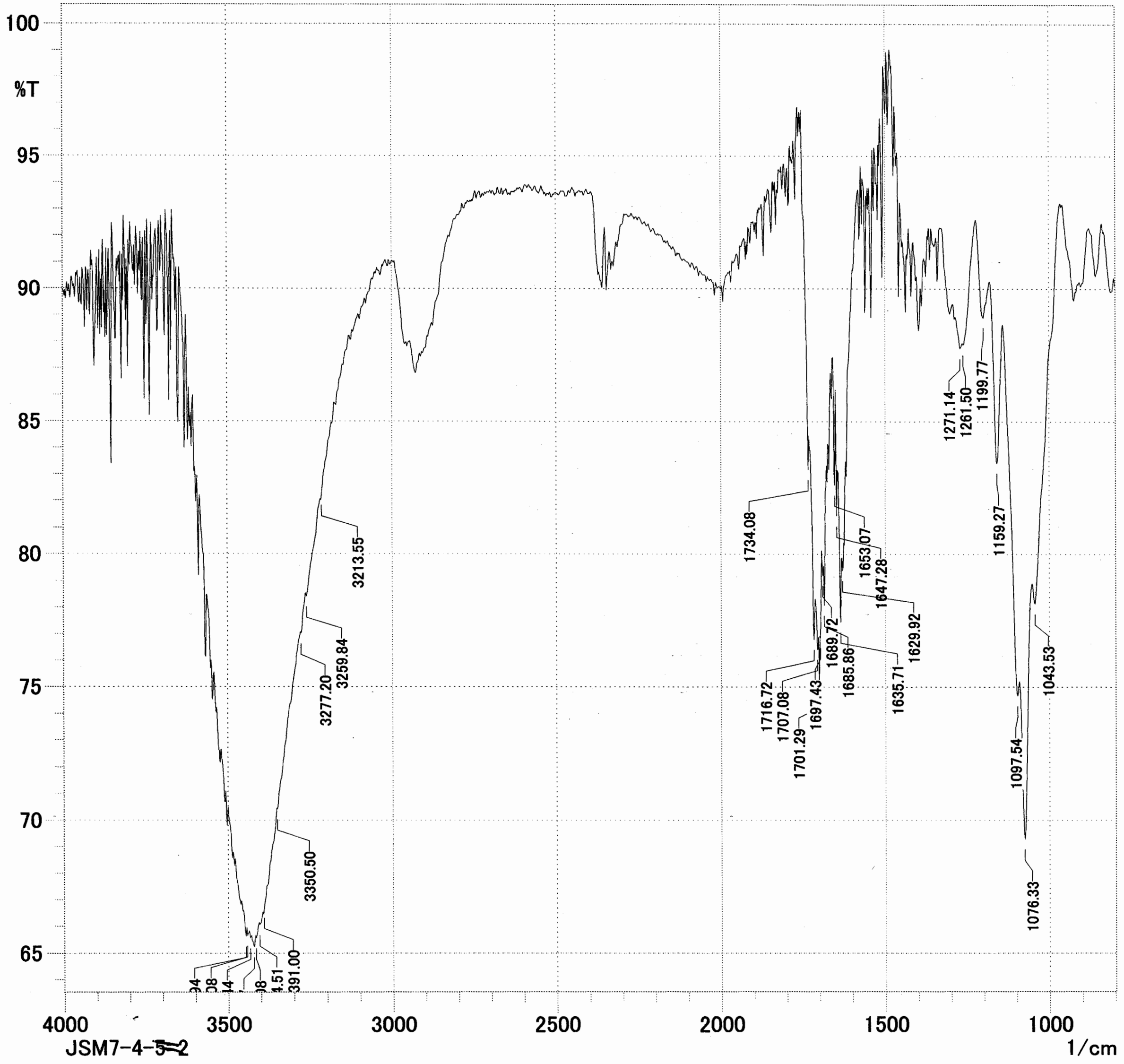

	ﾋﾟｰｸ	高さ	補正高さ	ﾍﾞｰｽ (H)	ﾍﾞｰｽ (L)	面積	補正面積
1	1043.53	78.15	2.34	1053.18	966.38	6.04	0.36
2	1076.33	69.3	7.43	1091.76	1055.11	4.9	0.76
3	1097.54	74.67	1.53	1141.91	1093.69	4.37	0.12
4	1159.27	83.43	5.8	1180.49	1143.84	2.34	0.54
5	1199.77	88.92	2.42	1222.92	1180.49	1.92	0.27
6	1261.5	87.9	0.28	1263.43	1222.92	1.84	0.03
7	1271.14	87.75	0.56	1284.65	1263.43	1.16	0.03
8	1629.92	79.38	1.26	1631.85	1614.49	1.48	0.14
9	1635.71	77.46	3.34	1645.35	1631.85	1.29	0.09
10	1647.28	81.43	2.73	1651.14	1645.35	0.46	0.04
11	1653.07	82.59	3.91	1658.85	1651.14	0.56	0.08
12	1685.86	78.08	2.22	1687.79	1678.14	0.9	0.04
13	1689.72	79.2	0.54	1693.57	1687.79	0.57	0.01
14	1697.43	76.86	1.14	1699.36	1693.57	0.61	0.02
15	1701.29	75.03	1.87	1703.22	1699.36	0.46	0.02
16	1707.08	76.42	1.04	1712.86	1703.22	1.09	0.02
17	1716.72	76.79	2.93	1728.29	1712.86	1.53	0.1
18	1734.08	83.18	2.29	1755.3	1732.15	1.04	0.03
19	3213.55	82.01	0.17	3215.47	3182.68	2.53	0
20	3259.84	78.42	0.43	3263.7	3219.33	4.28	0.06
21	3277.2	77.02	0.24	3279.13	3263.7	1.68	0.01
22	3350.5	70.42	0.21	3352.43	3279.13	9.65	0.01
23	3391	66.44	0.19	3392.93	3352.43	6.67	0.03
24	3404.51	66.05	0.14	3406.44	3396.79	1.73	0.01
25	3416.08	65.57	0.17	3418.01	3408.36	1.75	0.01
26	3421.87	65.24	0.43	3429.58	3418.01	2.13	0.02
27	3433.44	65.57	0.17	3437.3	3429.58	1.41	0
28	3443.08	65.66	0.25	3445.01	3437.3	1.4	0.01
29	3446.94	65.63	0.44	3454.66	3445.01	1.73	0.01

コメント;
JSM7-4-~~5=2~~ 4

日 時; 2019/12/21 17:46:48
積 算;
分 解;
ｱﾎﾟﾀﾞｲｾﾞｰｼｮﾝ;
分析者;Administrator

jasminumoside H (3)

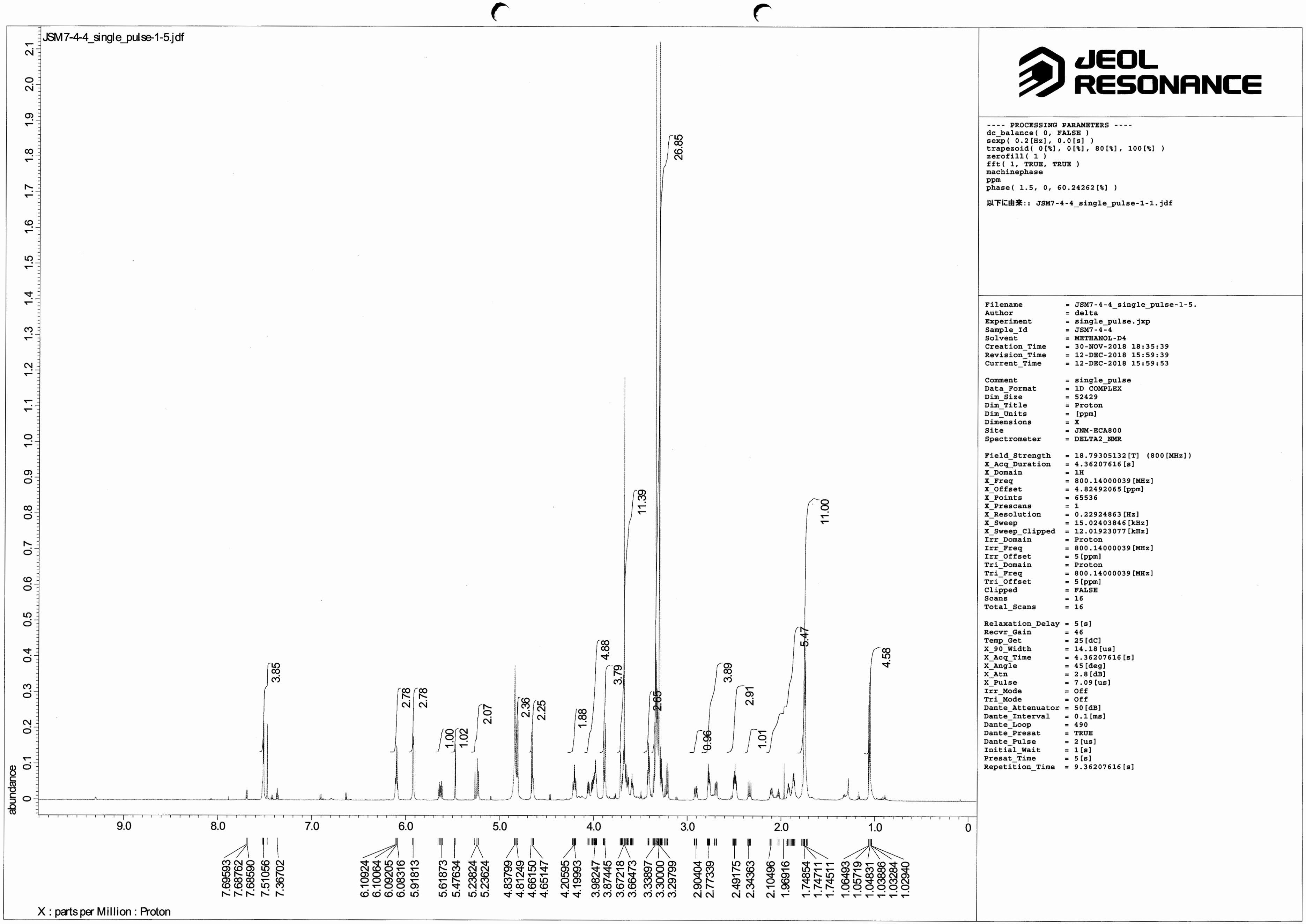

jasminumoside H (3)

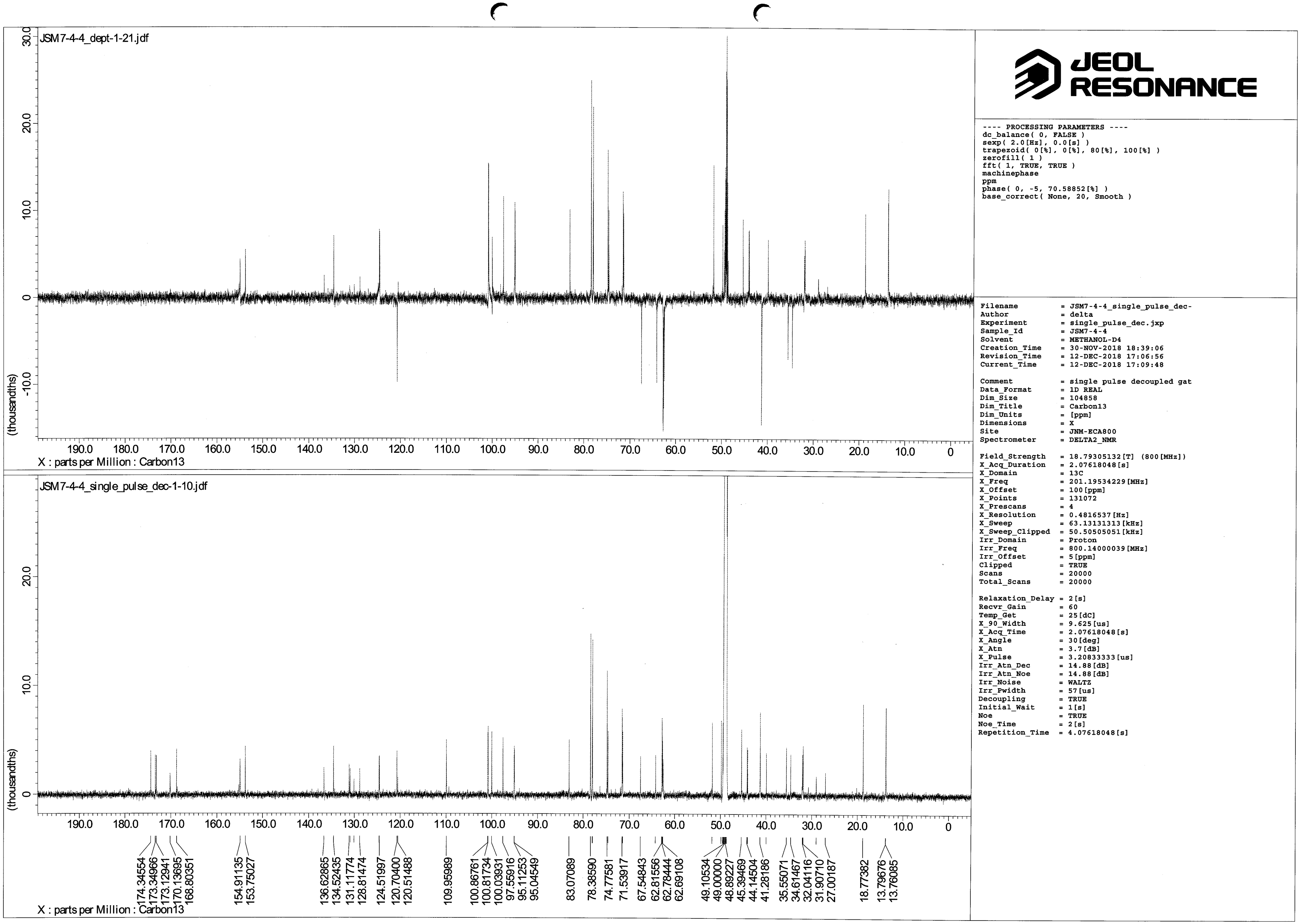

jasminumoside H (3)

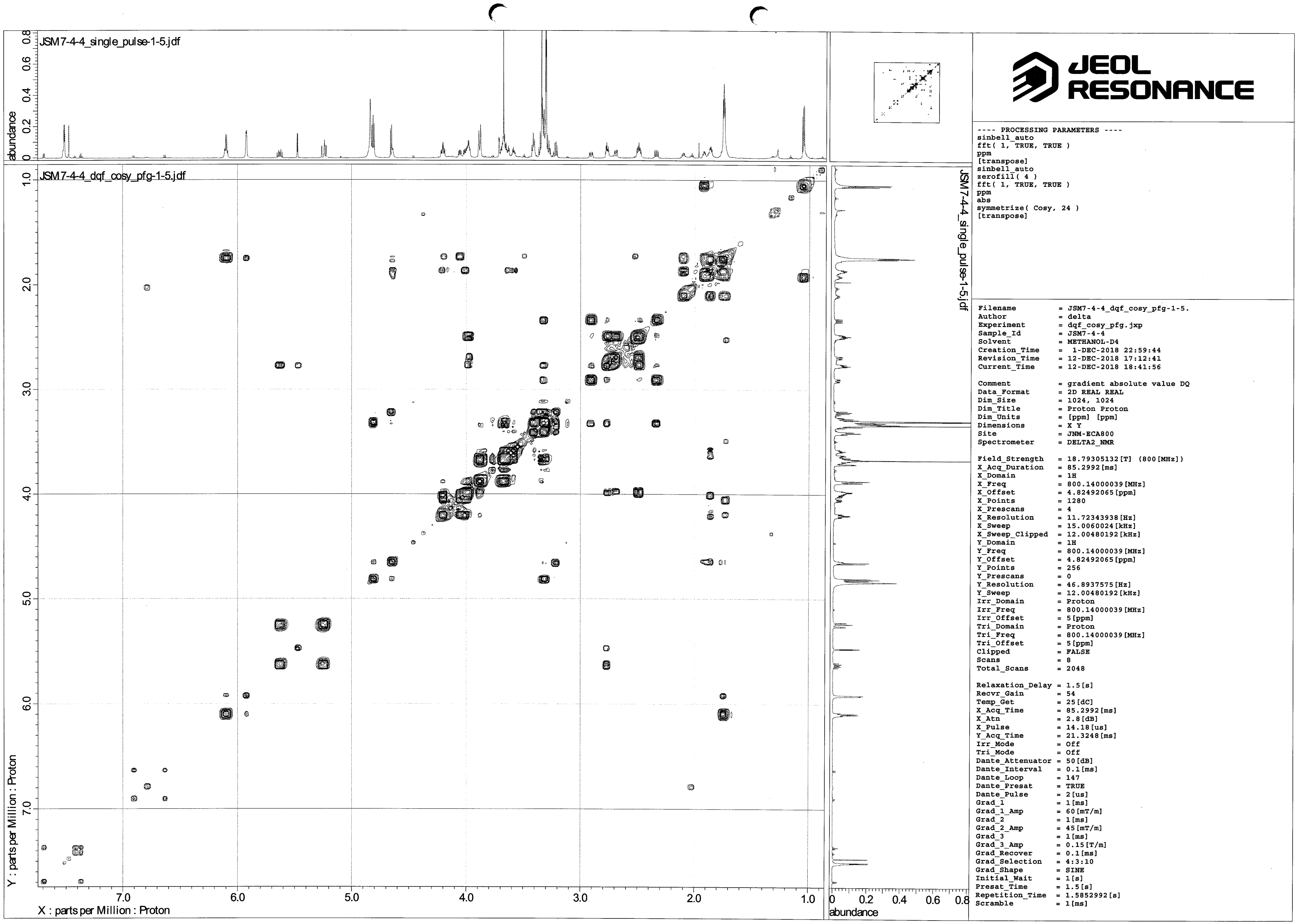

jasminumoside H (3)

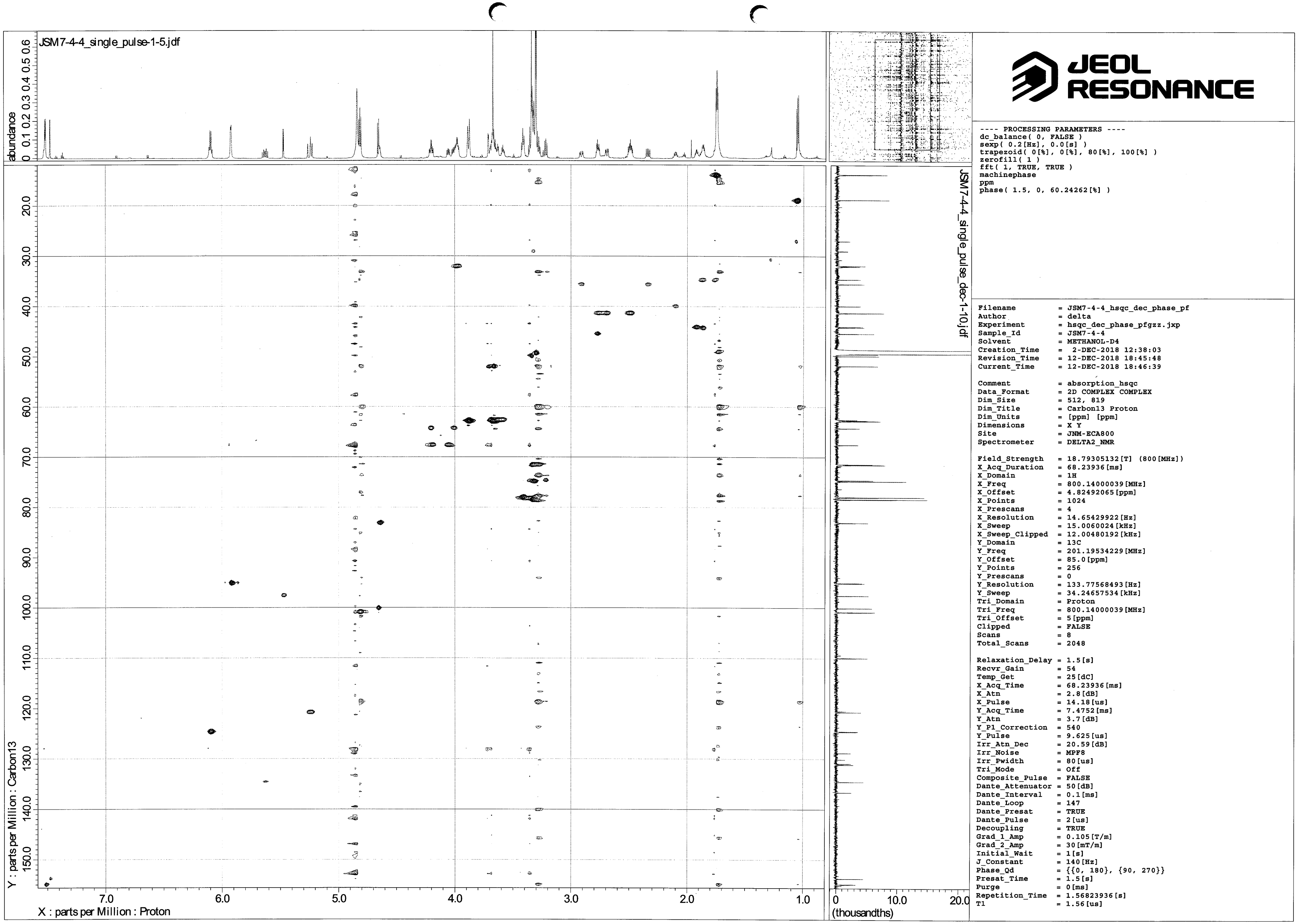

jasminumoside H (3)

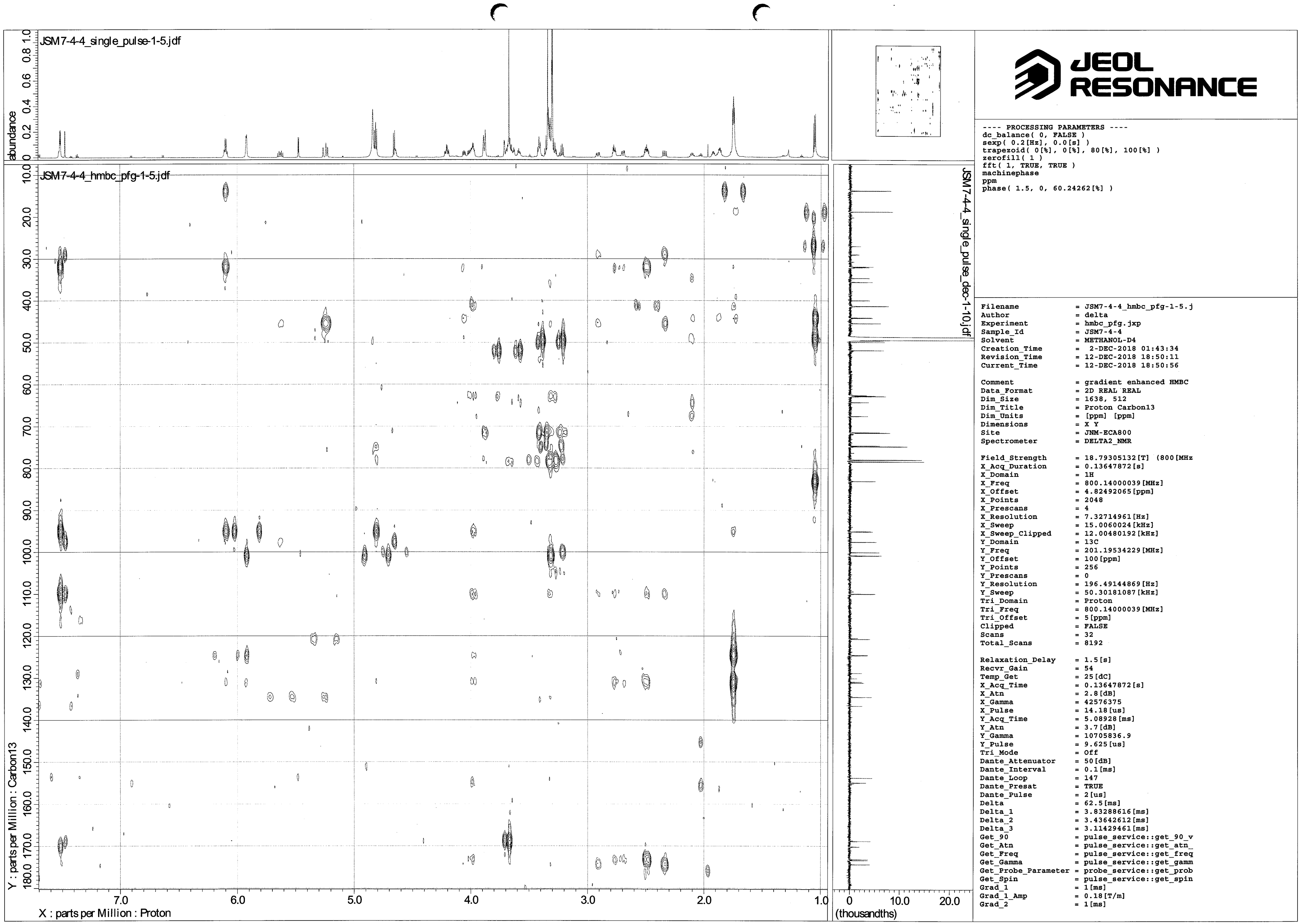

jasminumoside H (3)

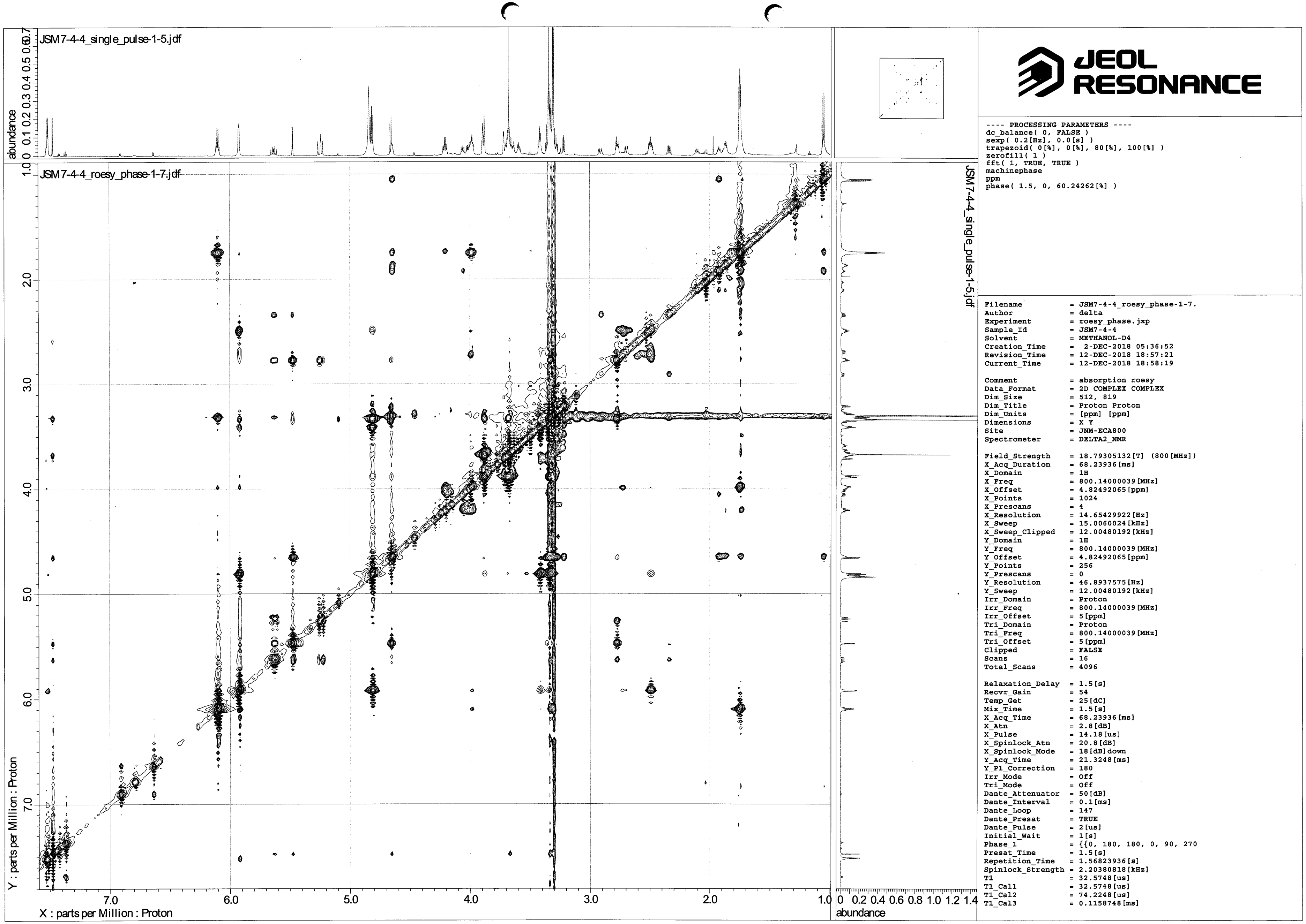

jasminumoside H (3)

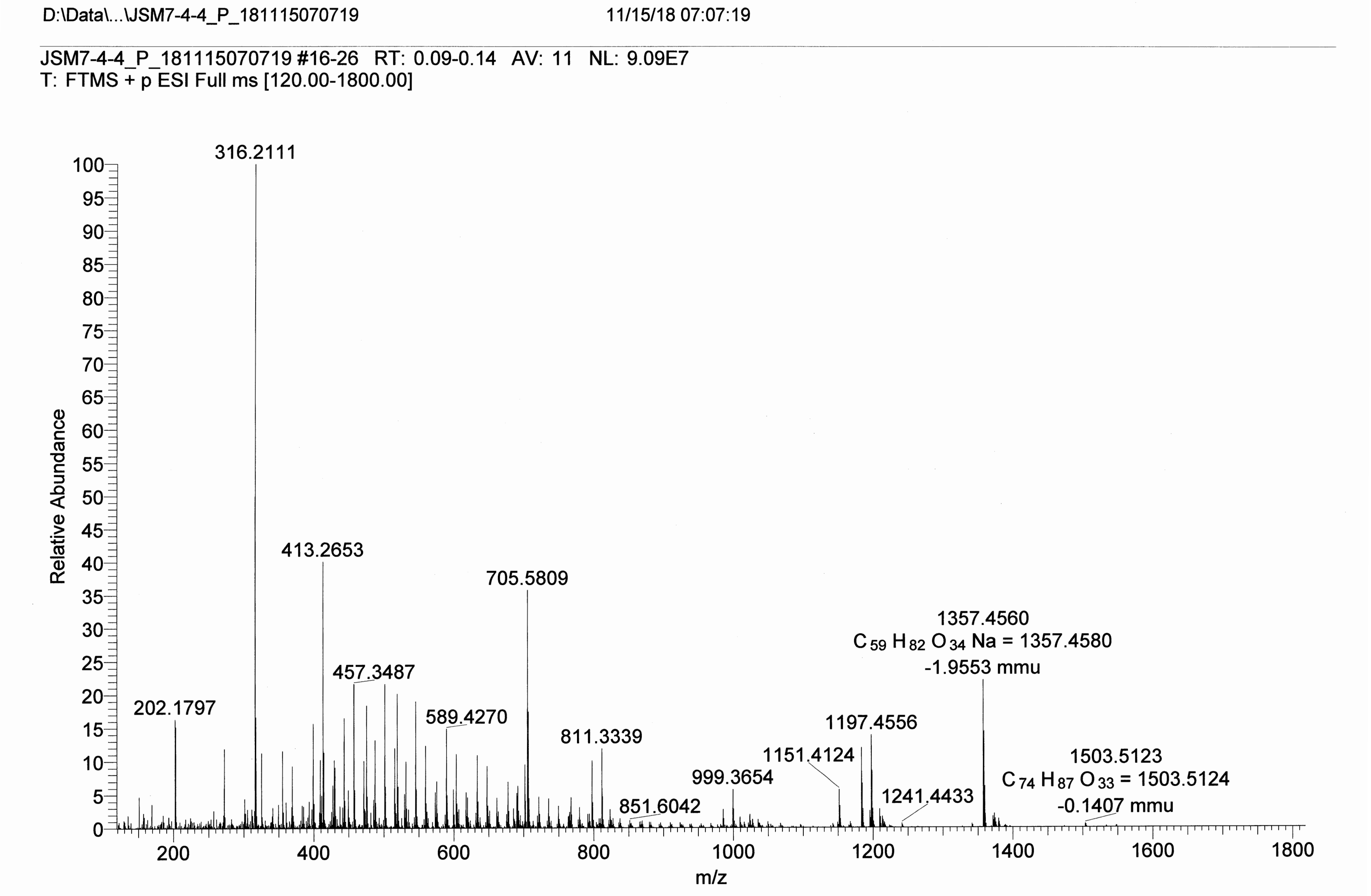

jasminumoside H (3)

D:\Data\...\JSM7-4-4_N_181115071201 11/15/18 07:12:01

JSM7-4-4_N_181115071201 #13-34 RT: 0.07-0.18 AV: 22 NL: 4.04E7
T: FTMS - p ESI Full ms [150.00-2000.00]

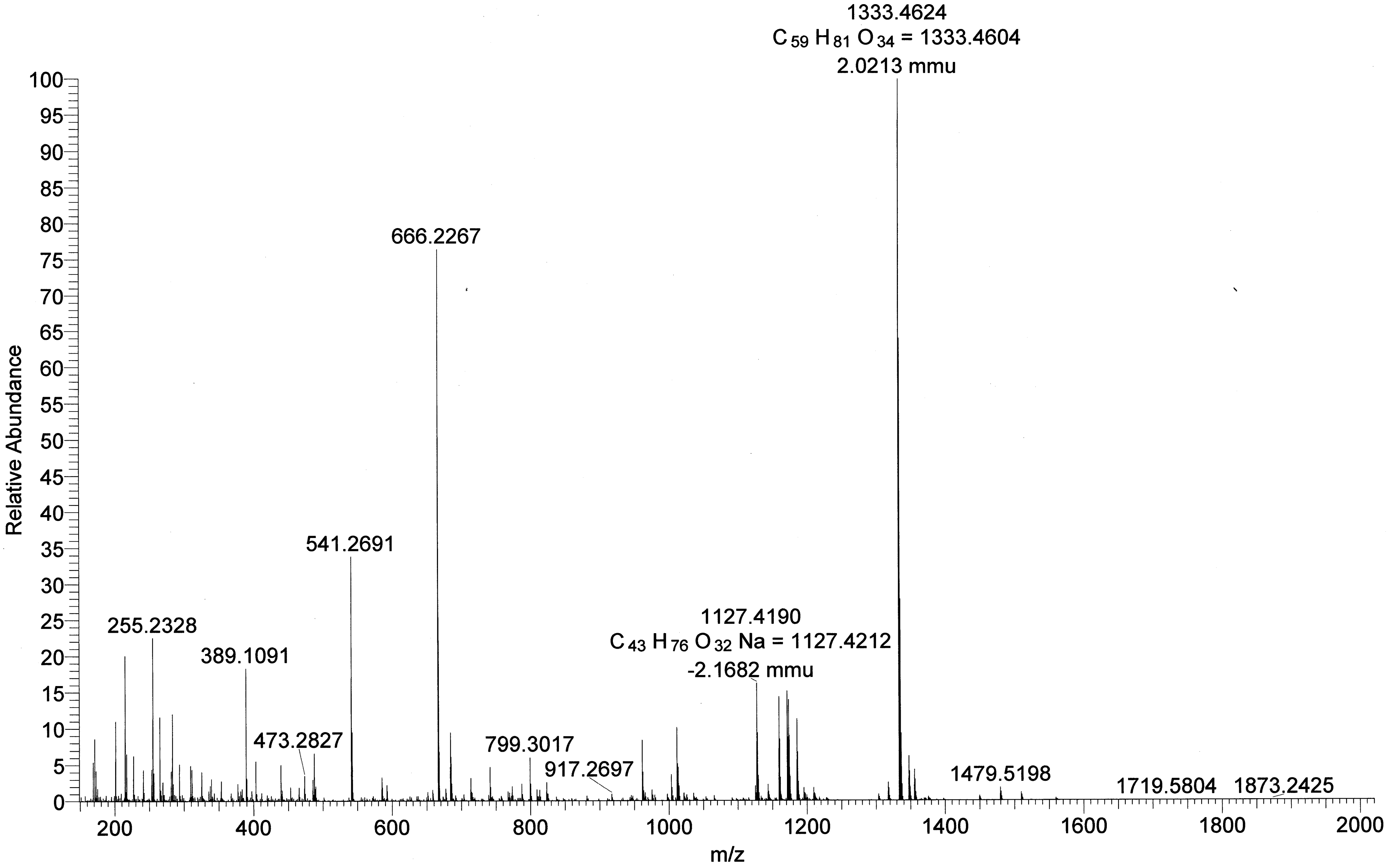

jasminumoside I (4)

SHIMADZU

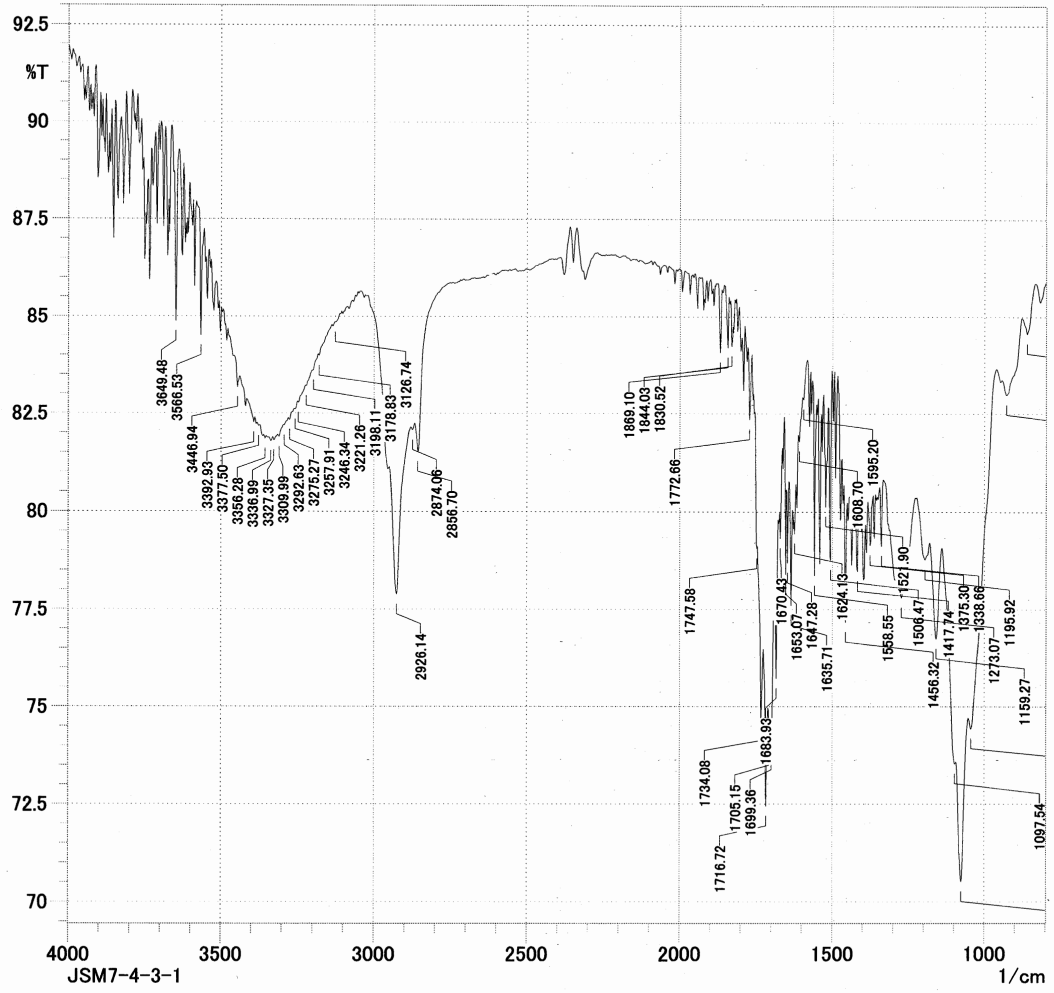

コメント;
JSM7-4-3-1

日 時; 2019/12/23 17:33:46
積 算; 20
分 解; 4 [1/cm]
ｱﾎﾟﾀﾞｲｾﾞｰｼｮﾝ; Happ-Genzel
分析者;Administrator

	ﾋﾟｰｸ	高さ	補正高さ	ﾍﾞｰｽ (H)	ﾍﾞｰｽ (L)	面積	補正面積
1	860.29	84.558	0.74	875.72	835.21	2.848	0.077
2	927.8	82.989	0.706	941.3	877.65	4.946	0.187
3	1043.53	74.436	1.038	1051.25	962.52	9.262	0.283
4	1076.33	70.517	3.499	1091.76	1053.18	5.396	0.387
5	1097.54	73.534	0.512	1139.98	1093.69	5.441	-0.001
6	1159.27	76.747	2.368	1180.49	1141.91	4.176	0.252
7	1195.92	78.777	0.783	1222.92	1182.41	4.088	0.104
8	1273.07	77.804	0.664	1282.72	1228.71	5.584	0.126
9	1338.66	79.133	1.59	1344.44	1332.87	1.118	0.043
10	1375.3	79.15	1.011	1381.09	1367.59	1.324	0.03
11	1417.74	78.484	1.762	1425.46	1413.88	1.157	0.05
12	1456.32	77.171	3.11	1462.11	1452.46	1.005	0.085
13	1506.47	78.769	4.456	1512.26	1502.61	0.881	0.112
14	1521.9	80.13	2.697	1529.62	1514.19	1.378	0.113
15	1558.55	78.371	4.607	1564.34	1552.76	1.037	0.097
16	1595.2	82.863	0.271	1599.06	1581.7	1.366	0.006
17	1608.7	81.796	0.434	1612.56	1599.06	1.142	0.015
18	1624.13	79.442	0.654	1626.06	1620.27	0.566	0.013
19	1635.71	77.599	2.727	1641.49	1631.85	0.98	0.063
20	1647.28	78.7	1.967	1651.14	1643.42	0.753	0.033
21	1653.07	78.401	2.624	1658.85	1651.14	0.725	0.039
22	1670.43	79.317	1.088	1672.36	1664.64	0.732	0.018
23	1683.93	75.728	3.106	1691.64	1678.14	1.487	0.092
24	1699.36	73.885	1.681	1703.22	1693.57	1.21	0.064
25	1705.15	74.008	0.647	1710.93	1703.22	0.986	0.011
26	1716.72	72.446	3.092	1726.36	1710.93	1.973	0.101
27	1734.08	74.641	2.527	1745.65	1728.29	2.008	0.084
28	1747.58	79.06	0.747	1755.3	1745.65	0.919	0.027
29	1772.66	82.354	1.56	1776.52	1766.87	0.777	0.038
30	1830.52	84.23	1.194	1834.38	1820.88	0.966	0.043
31	1844.03	84.199	1.39	1853.67	1840.17	0.948	0.039
32	1869.1	84.072	1.701	1874.89	1863.32	0.814	0.041
33	2856.7	81.528	1.155	2866.34	2791.12	5.608	0.069
34	2874.06	82.077	0.093	2877.92	2868.27	0.825	0.003
35	2926.14	77.881	3.588	2949.29	2879.85	6.614	0.501
36	3126.74	84.845	0.026	3128.67	3105.53	1.638	0.004
37	3178.83	83.989	0.073	3180.75	3138.32	3.122	0.002
38	3198.11	83.61	0.045	3200.04	3182.68	1.329	0.001
39	3221.26	83.209	0.047	3223.19	3201.97	1.672	0.003
40	3246.34	82.782	0.064	3248.27	3223.19	2.028	0.003
41	3257.91	82.608	0.045	3259.84	3250.19	0.797	0.002
42	3275.27	82.338	0.104	3279.13	3269.48	0.81	0.003
43	3292.63	82.189	0.035	3294.56	3281.06	1.144	0.001
44	3309.99	81.906	0.095	3313.85	3296.49	1.491	0.003
45	3327.35	81.817	0.085	3331.21	3321.56	0.839	0.002
46	3336.99	81.794	0.102	3344.71	3331.21	1.174	0.003
47	3356.28	81.863	0.076	3360.14	3352.43	0.669	0.001
48	3377.5	82.177	0.041	3381.36	3375.57	0.492	0.001
49	3392.93	82.275	0.195	3416.08	3389.07	2.244	0.008
50	3446.94	83.18	0.51	3456.59	3439.23	1.357	0.015
51	3566.53	84.507	2.944	3572.32	3558.82	0.871	0.079

jasminumoside I (4)

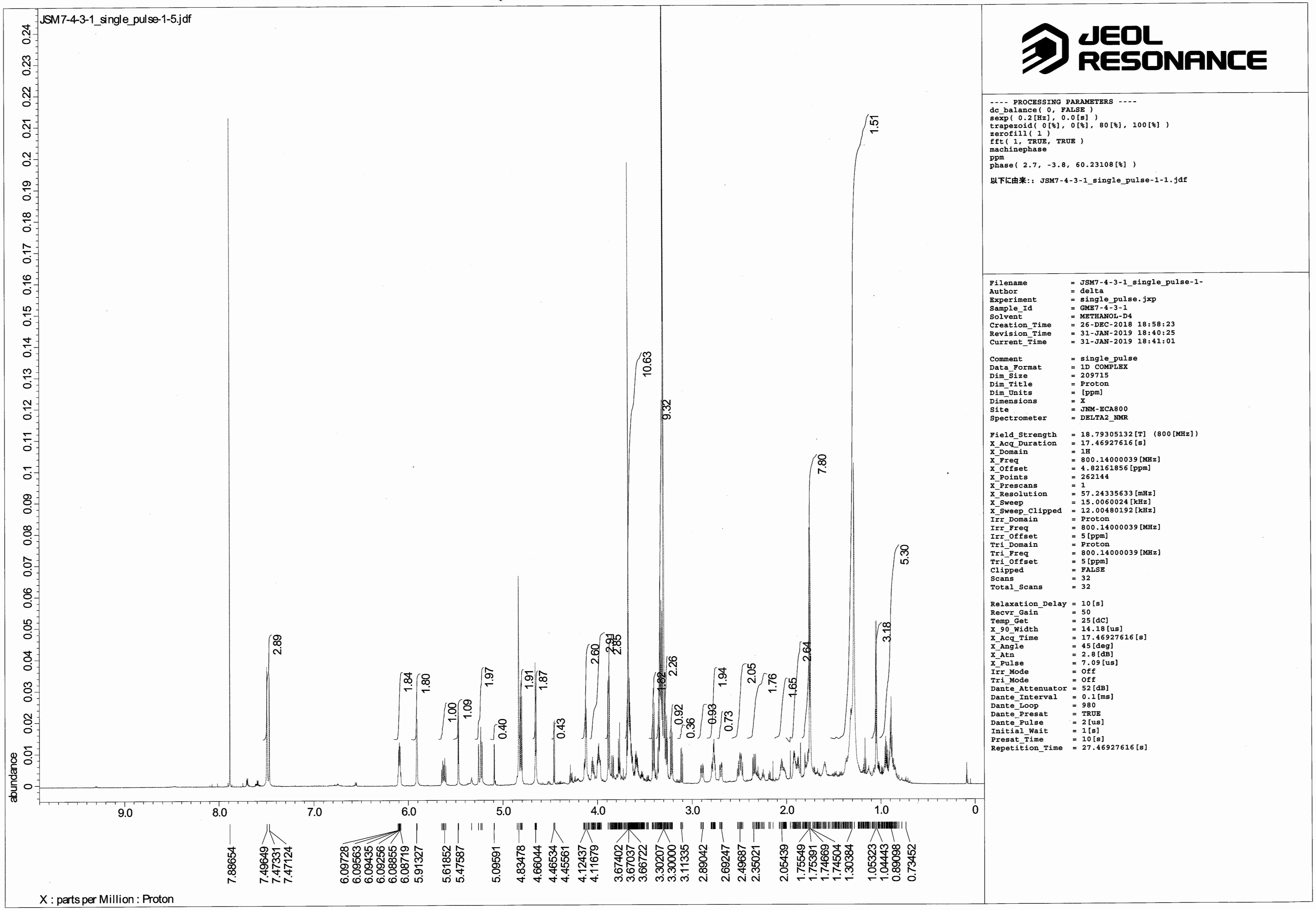

jasminumoside I (4)

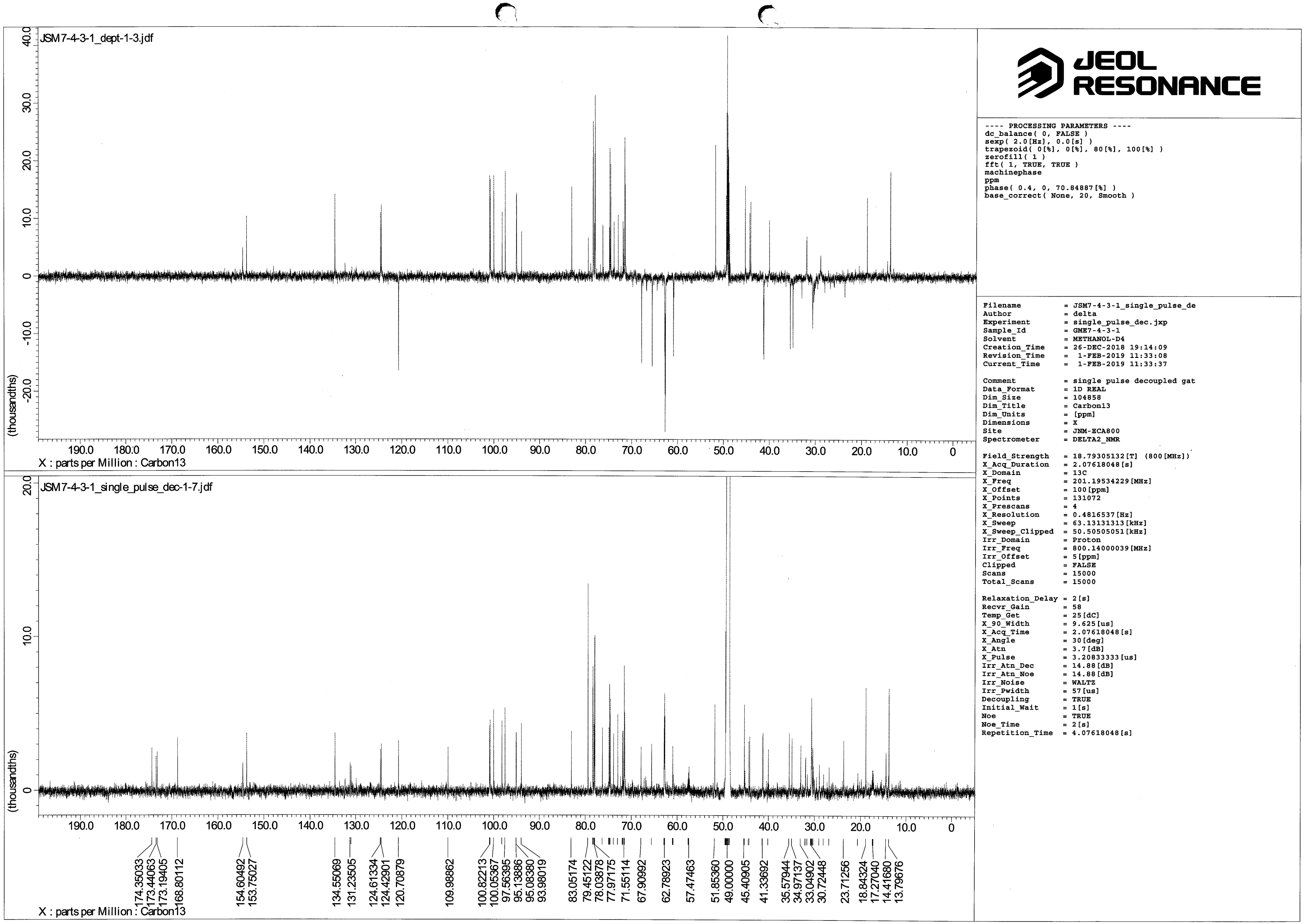

jasminumoside I (4)

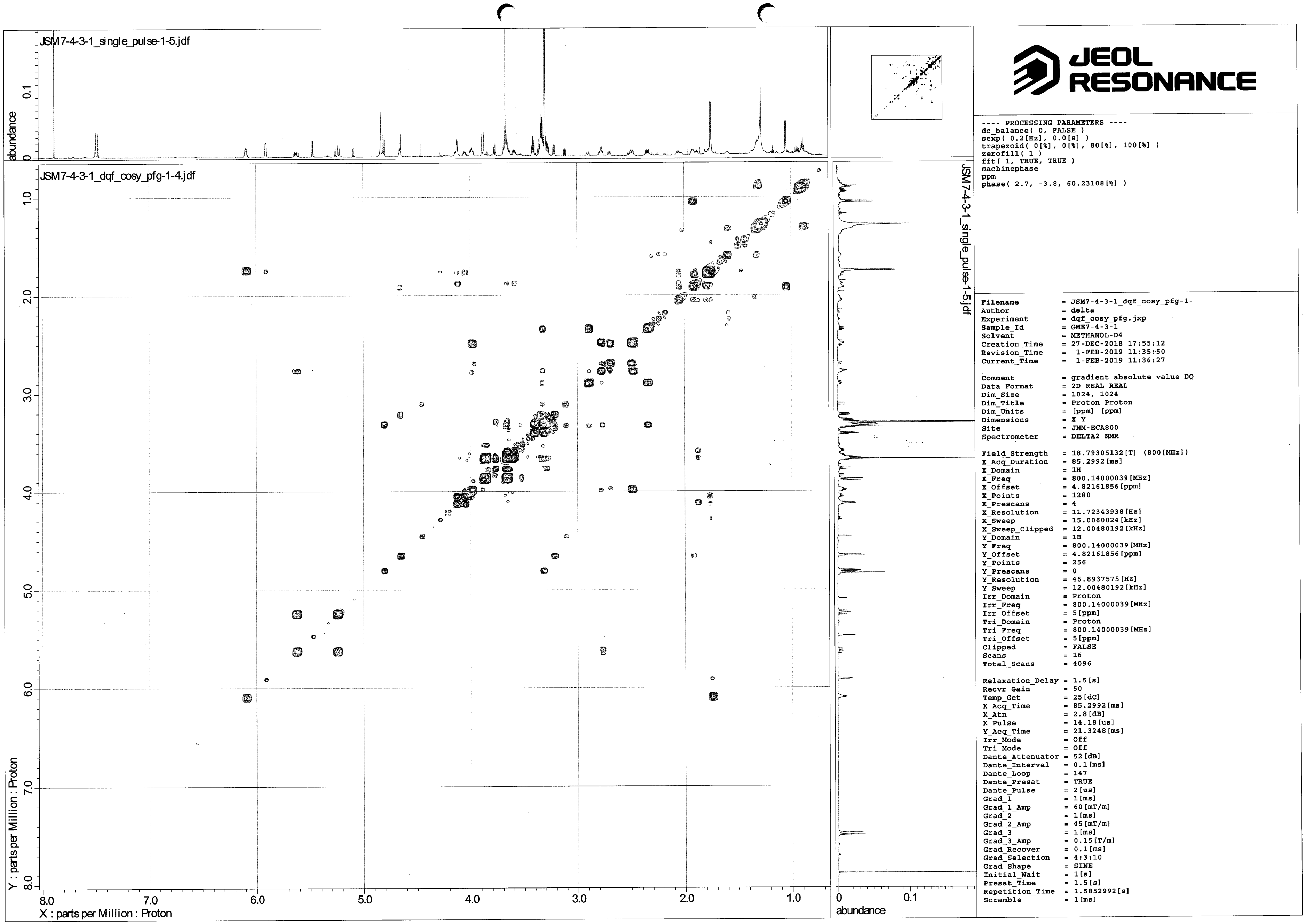

jasminumoside I (4)

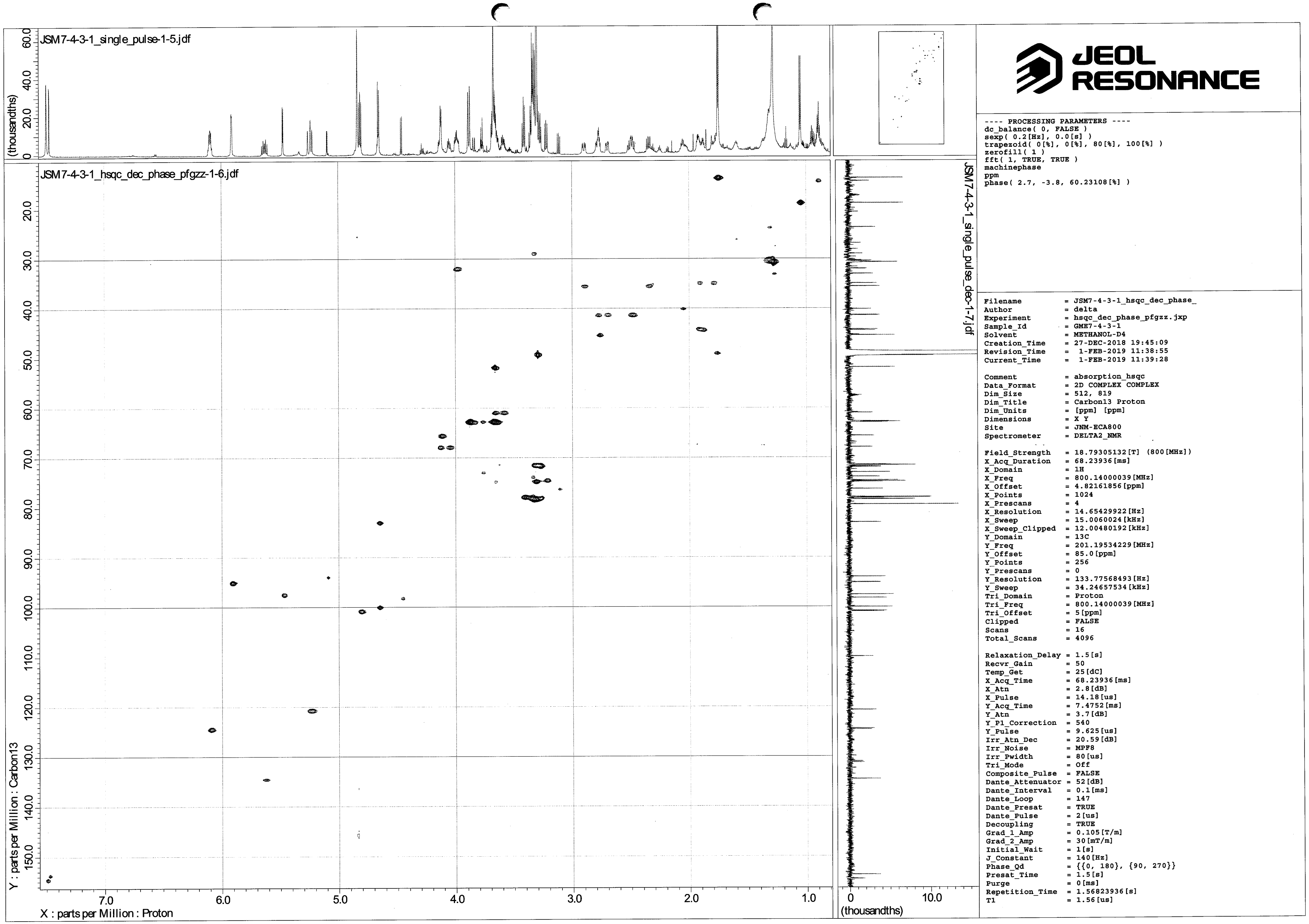

jasminumoside I (4)

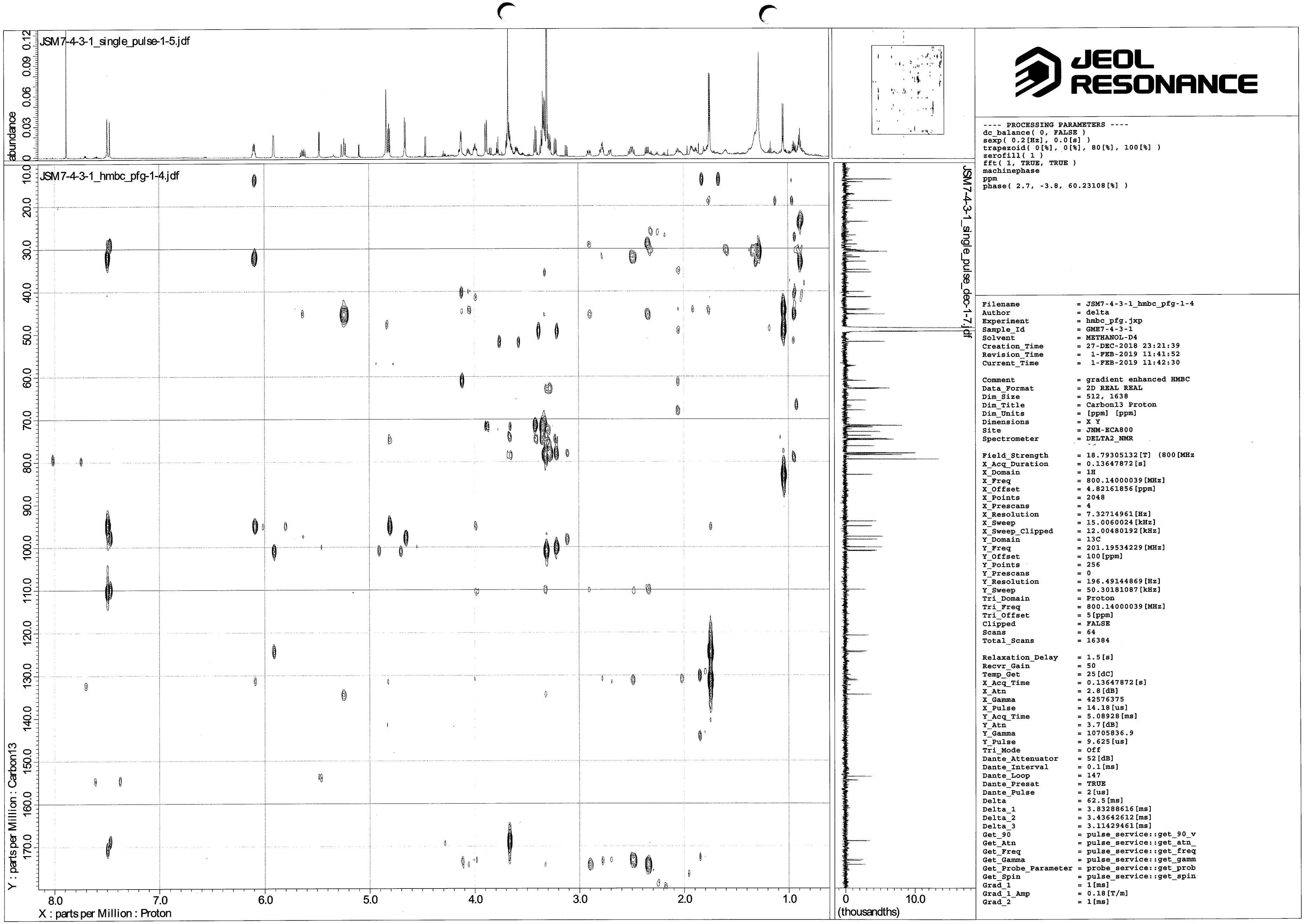

jasminumoside I (4)

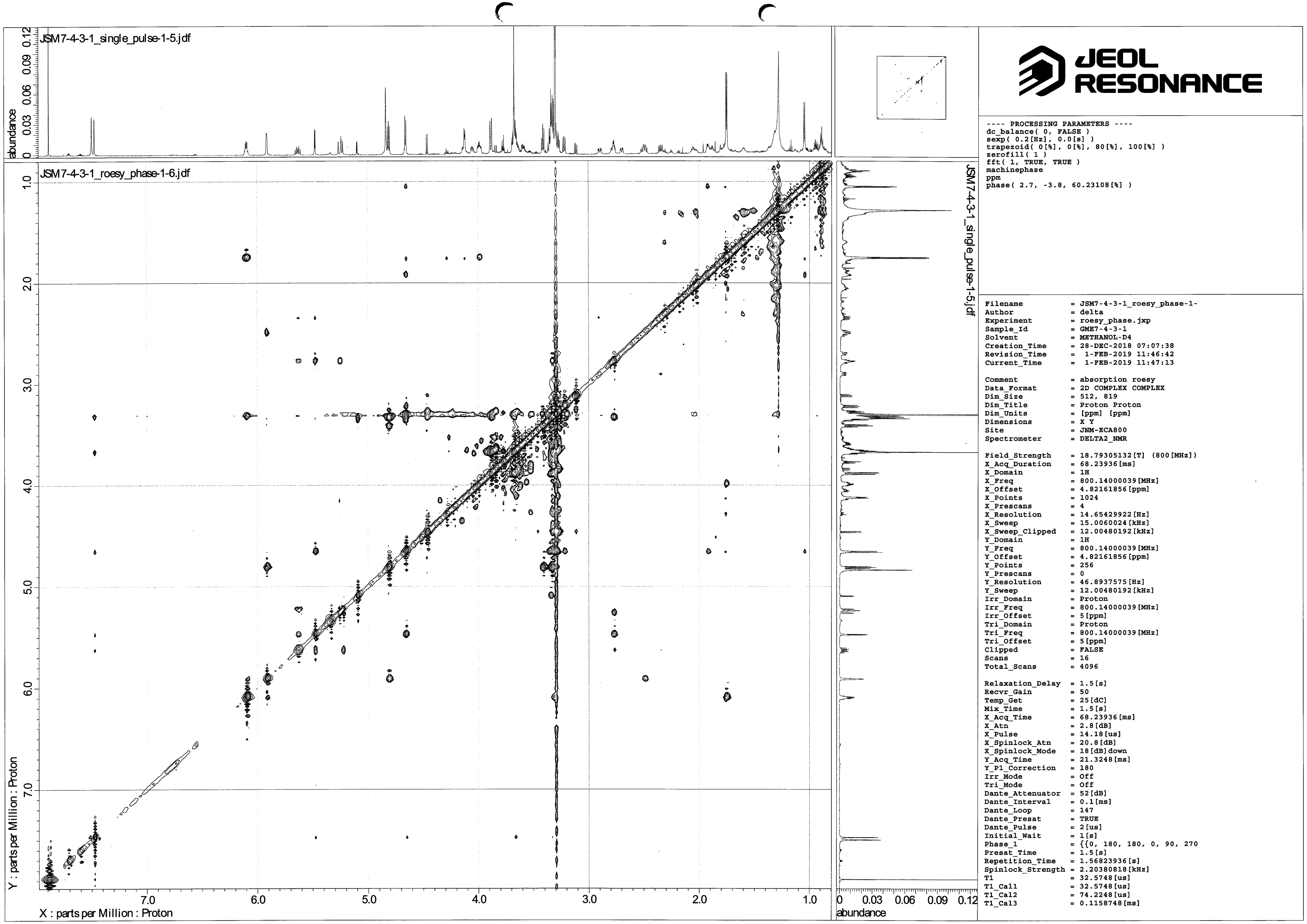

jasminumoside I (4)

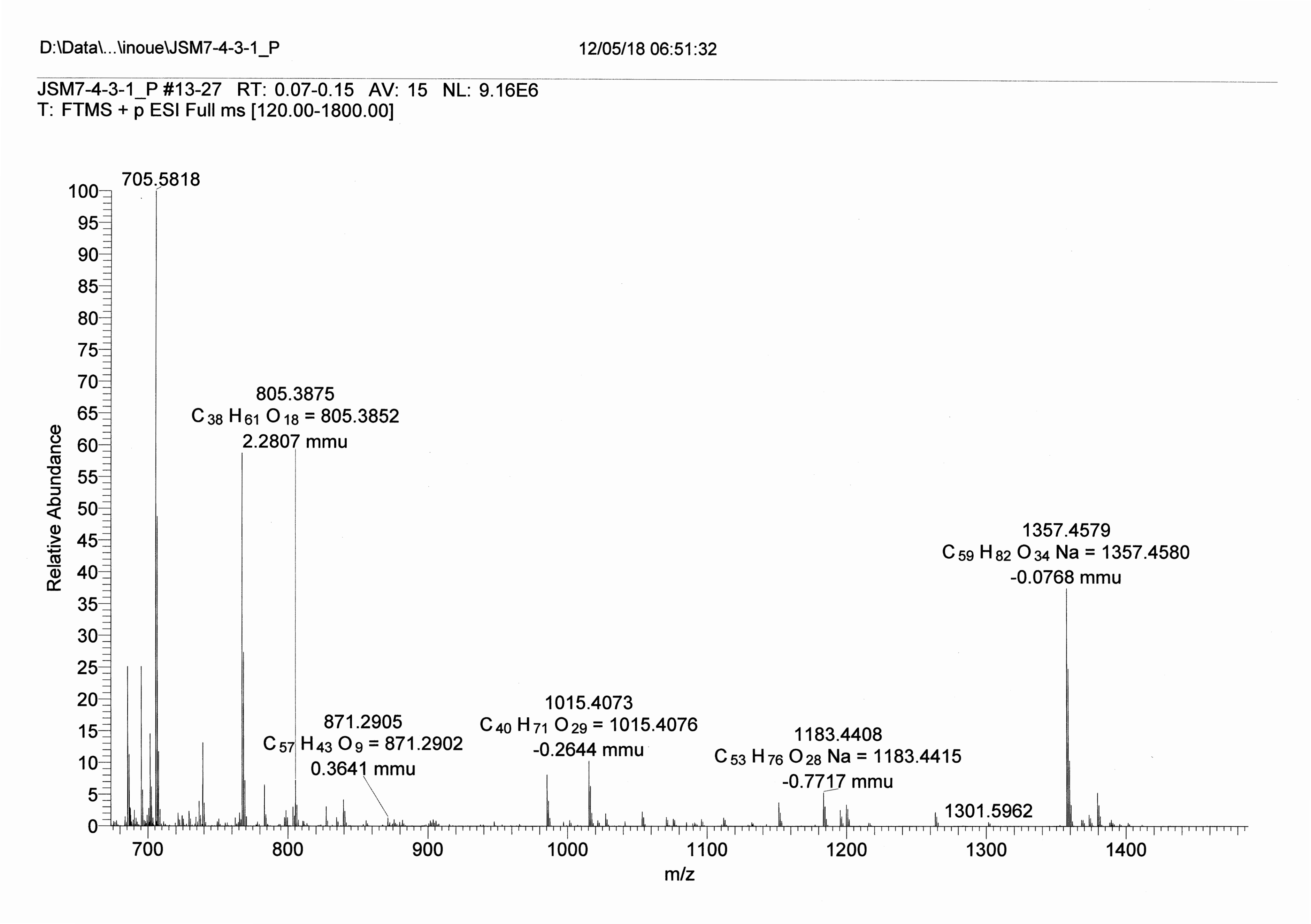

jasminumoside I (4)

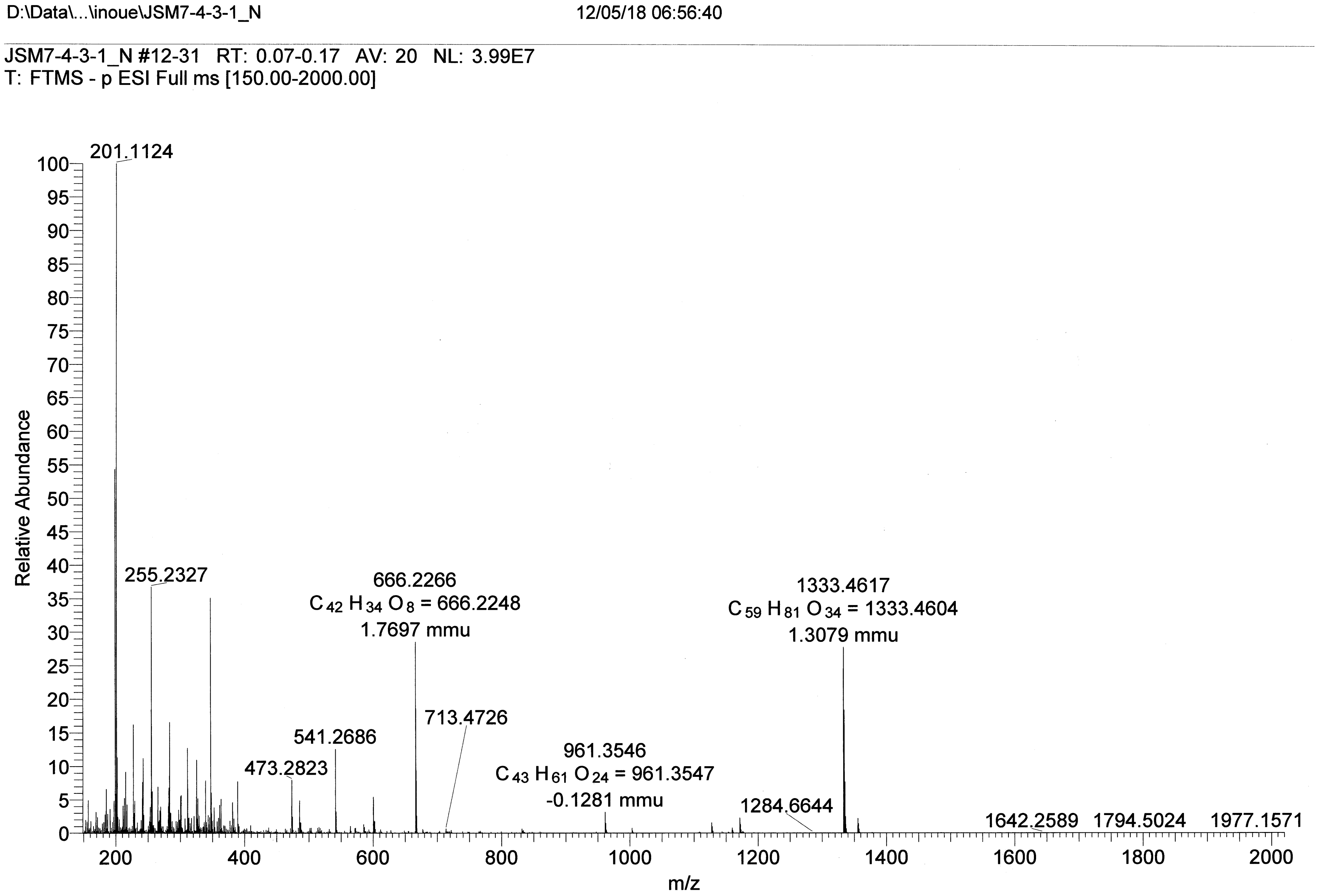

jasminumoside J (5)

SHIMADZU

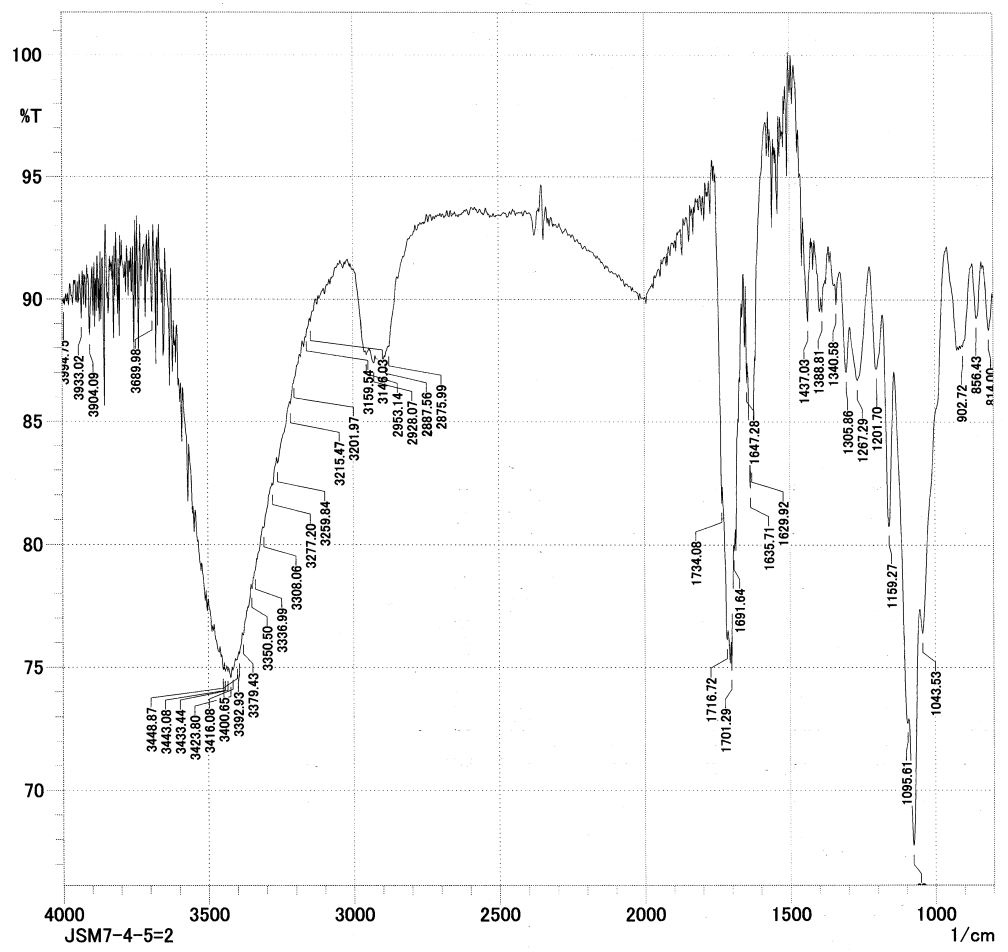

	ﾋﾟｰｸ	高さ	補正高さ	ﾍﾞｰｽ (H)	ﾍﾞｰｽ (L)	面積	補正面積
1	814	88.74	1.96	831.36	802.42	1.37	0.15
2	856.43	89.22	2.25	869.93	841	1.3	0.18
3	902.72	88.07	0.44	906.58	871.86	1.64	0.05
4	1043.53	76.37	2.53	1053.18	960.59	7.11	0.43
5	1076.33	67.74	7.03	1091.76	1055.11	5.31	0.77
6	1095.61	72.73	1.27	1141.91	1091.76	4.96	0.01
7	1159.27	80.75	7.35	1180.49	1141.91	2.85	0.73
8	1201.7	87.16	3.08	1224.85	1182.41	2.25	0.37
9	1267.29	86.69	3.12	1292.36	1226.78	3.54	0.58
10	1305.86	87.02	2.65	1327.08	1294.29	1.71	0.2
11	1340.58	89.8	0.96	1344.44	1329.01	0.66	0.02
12	1388.81	89.49	0.48	1390.74	1379.16	0.51	0.01
13	1437.03	89.1	3.5	1452.46	1427.39	1.05	0.21
14	1629.92	83.29	0.85	1631.85	1581.7	1.83	0.03
15	1635.71	82.29	2.09	1645.35	1633.78	0.85	0.07
16	1647.28	86.6	1.84	1651.14	1645.35	0.32	0.03
17	1691.64	79.75	0.9	1693.57	1674.28	1.59	0.09
18	1701.29	74.88	3.71	1712.86	1693.57	2.23	0.19
19	1716.72	76.12	1.54	1732.15	1712.86	1.99	0.05
20	1734.08	81.68	1.72	1755.3	1732.15	1.26	0.03
21	2875.99	88.05	0.24	2877.92	2848.98	1.4	0.02
22	2887.56	87.63	0.08	2889.49	2879.85	0.54	0
23	2928.07	87.39	0.36	2943.5	2924.21	1.11	0.02
24	2953.14	87.72	0.18	2955.07	2945.43	0.54	0
25	3146.03	89.08	0.27	3149.89	3140.25	0.47	0.01
26	3159.54	88.44	0.13	3161.46	3149.89	0.59	0
27	3201.97	86.56	0.12	3203.9	3184.61	1.15	0
28	3215.47	85.71	0.25	3217.4	3207.76	0.63	0.01
29	3259.84	83.29	0.24	3261.77	3219.33	3.1	0.03
30	3277.2	82.38	0.23	3279.13	3263.7	1.25	0.01
31	3308.06	80.67	0.26	3311.92	3279.13	2.9	0.02
32	3336.99	78.92	0.13	3338.92	3311.92	2.64	0.01
33	3350.5	78.21	0.25	3352.43	3340.85	1.22	0.01
34	3379.43	76.29	0.36	3383.29	3354.35	3.26	0.04
35	3392.93	75.53	0.23	3394.86	3383.29	1.39	0.01
36	3400.65	75.31	0.16	3406.44	3396.79	1.18	0.01
37	3416.08	74.87	0.2	3418.01	3406.44	1.44	0.01
38	3423.8	74.58	0.41	3429.58	3418.01	1.46	0.02
39	3433.44	74.82	0.2	3437.3	3429.58	0.97	0
40	3443.08	74.82	0.32	3445.01	3437.3	0.97	0.01
41	3448.87	74.91	0.41	3454.66	3445.01	1.2	0.01
42	3689.98	89.49	3.14	3697.7	3686.13	0.47	0.08
43	3904.09	88.52	2.23	3907.95	3898.3	0.46	0.06
44	3933.02	89.22	1.77	3938.81	3929.17	0.43	0.04
45	3994.75	89.83	0.2	4000.54	3992.82	0.36	0

ｺﾒﾝﾄ;
JSM7-4-5=2

日 時; 2019/12/21 16:58:48
積 算;
分 解;
ｱﾎﾟﾀﾞｲｾﾞｰｼｮﾝ;
分析者;Administrator

jasminumoside J (5)

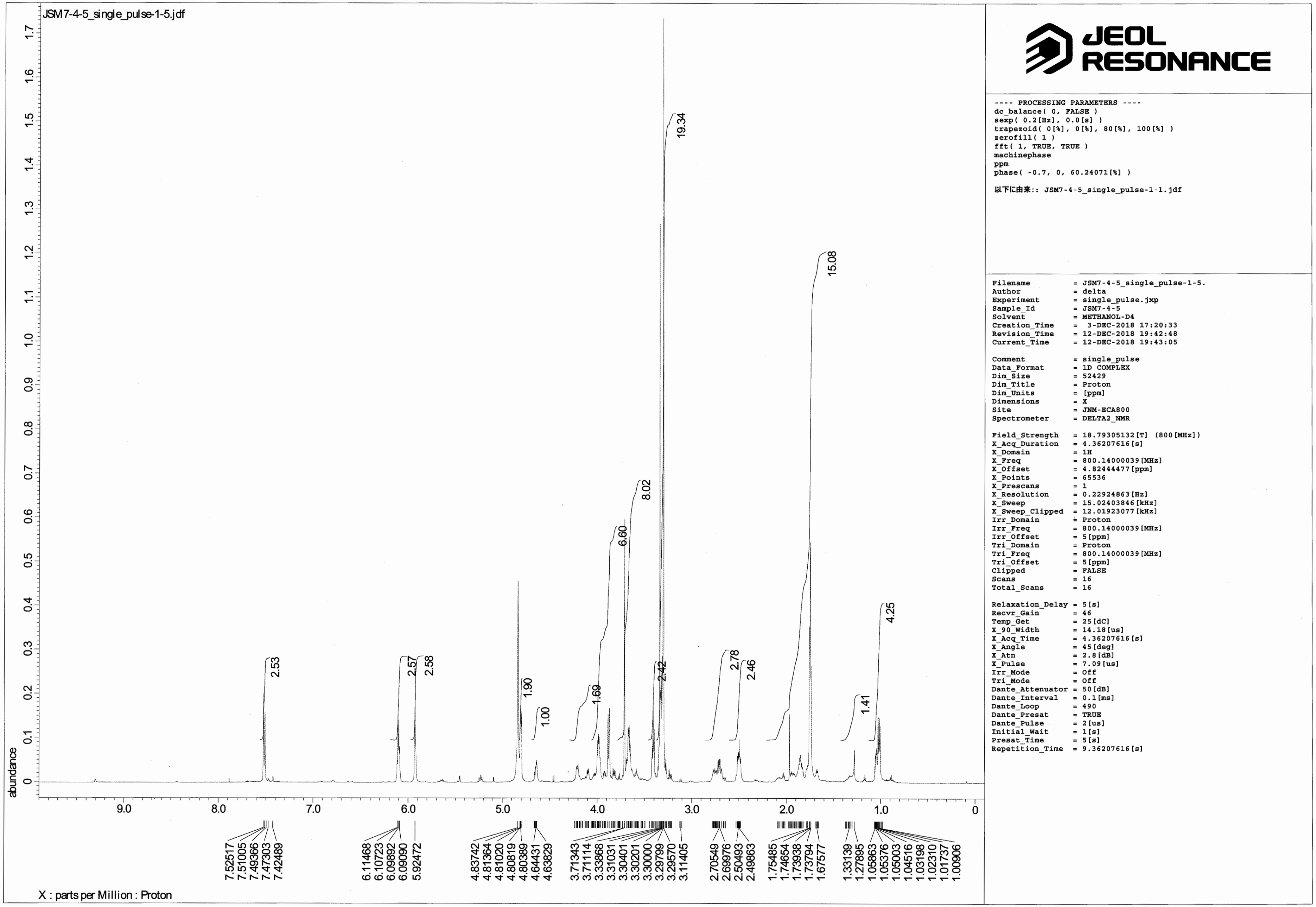

jasminumoside J (5)

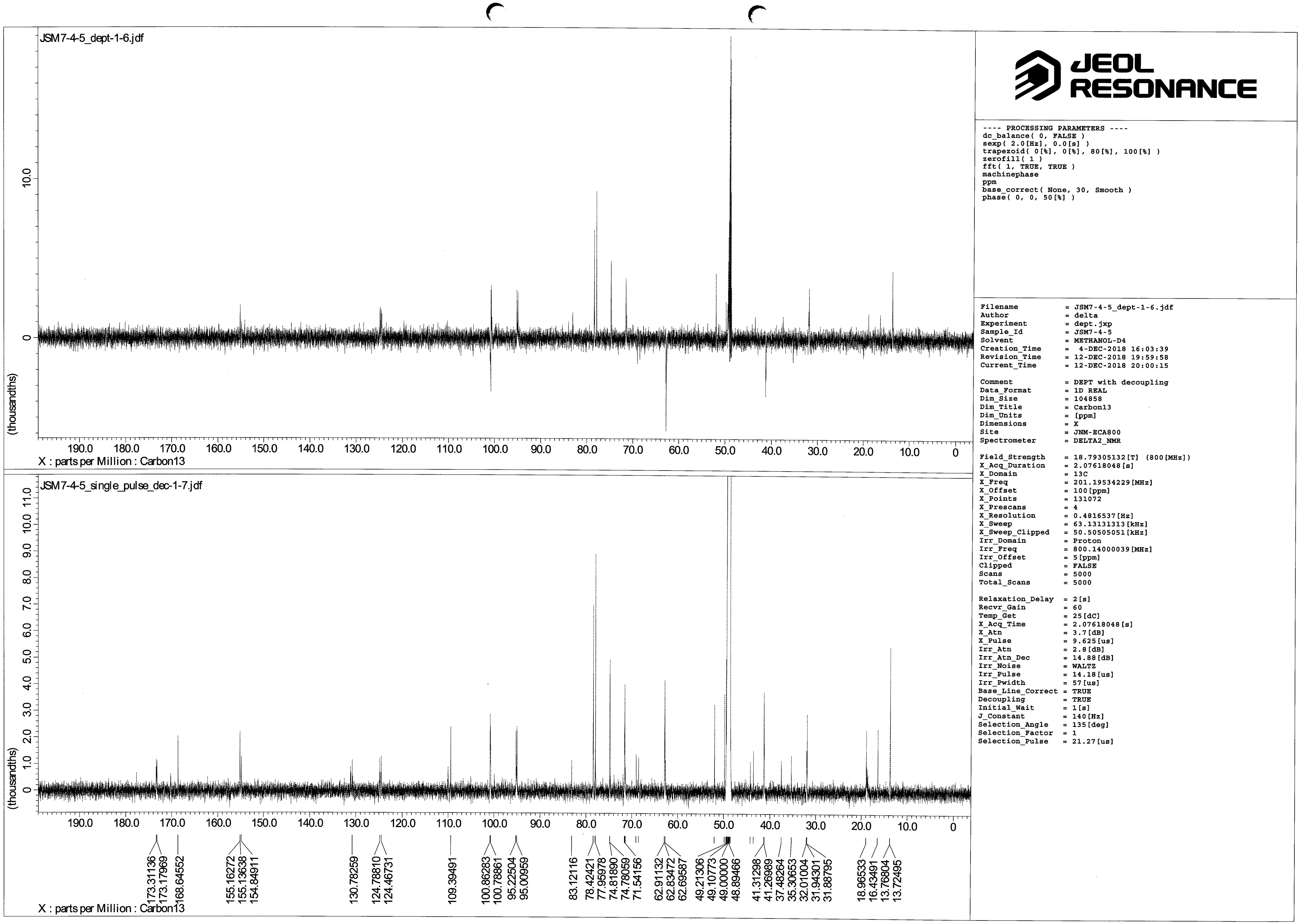

jasminumoside J (5)

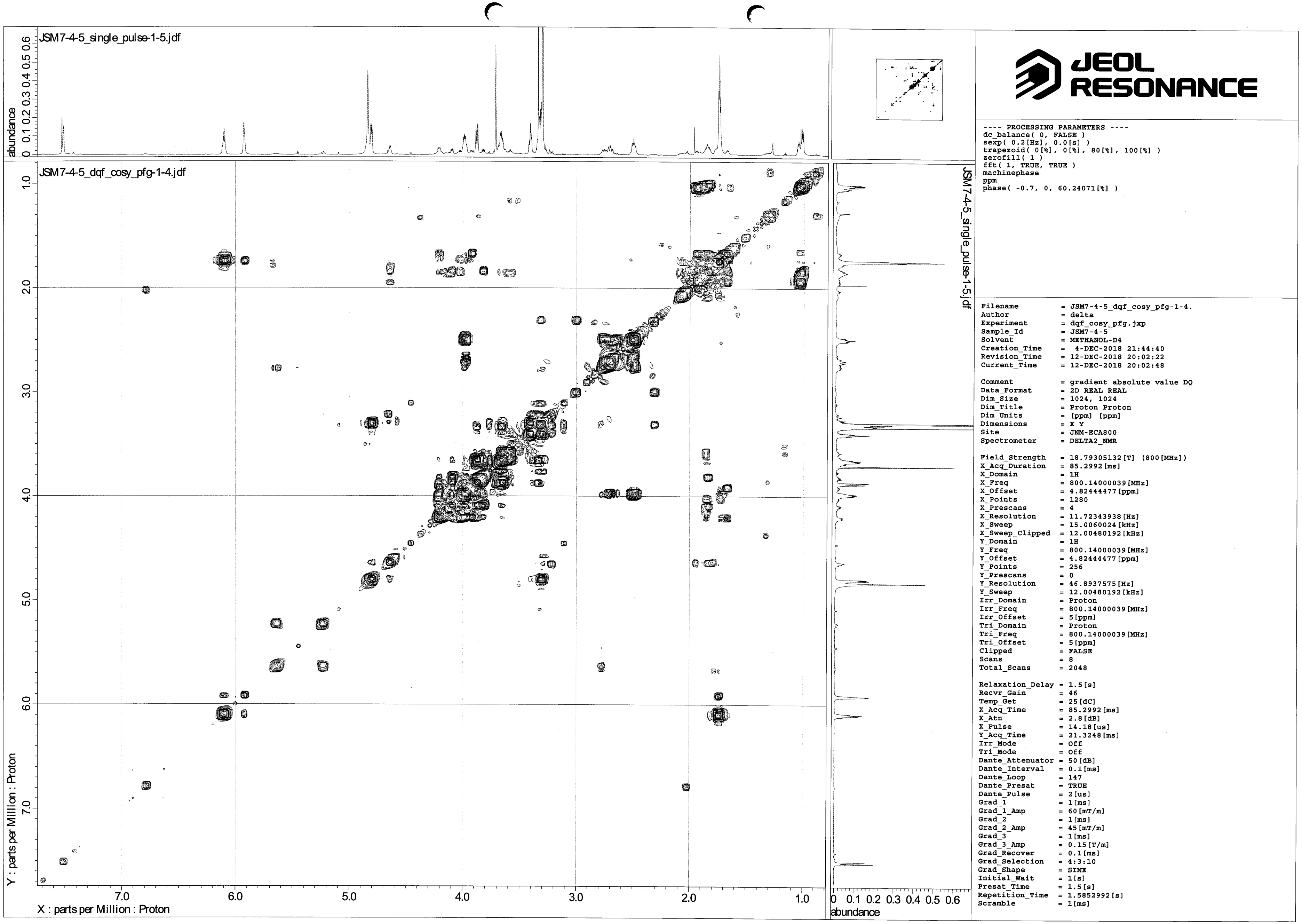

jasminumoside J (5)

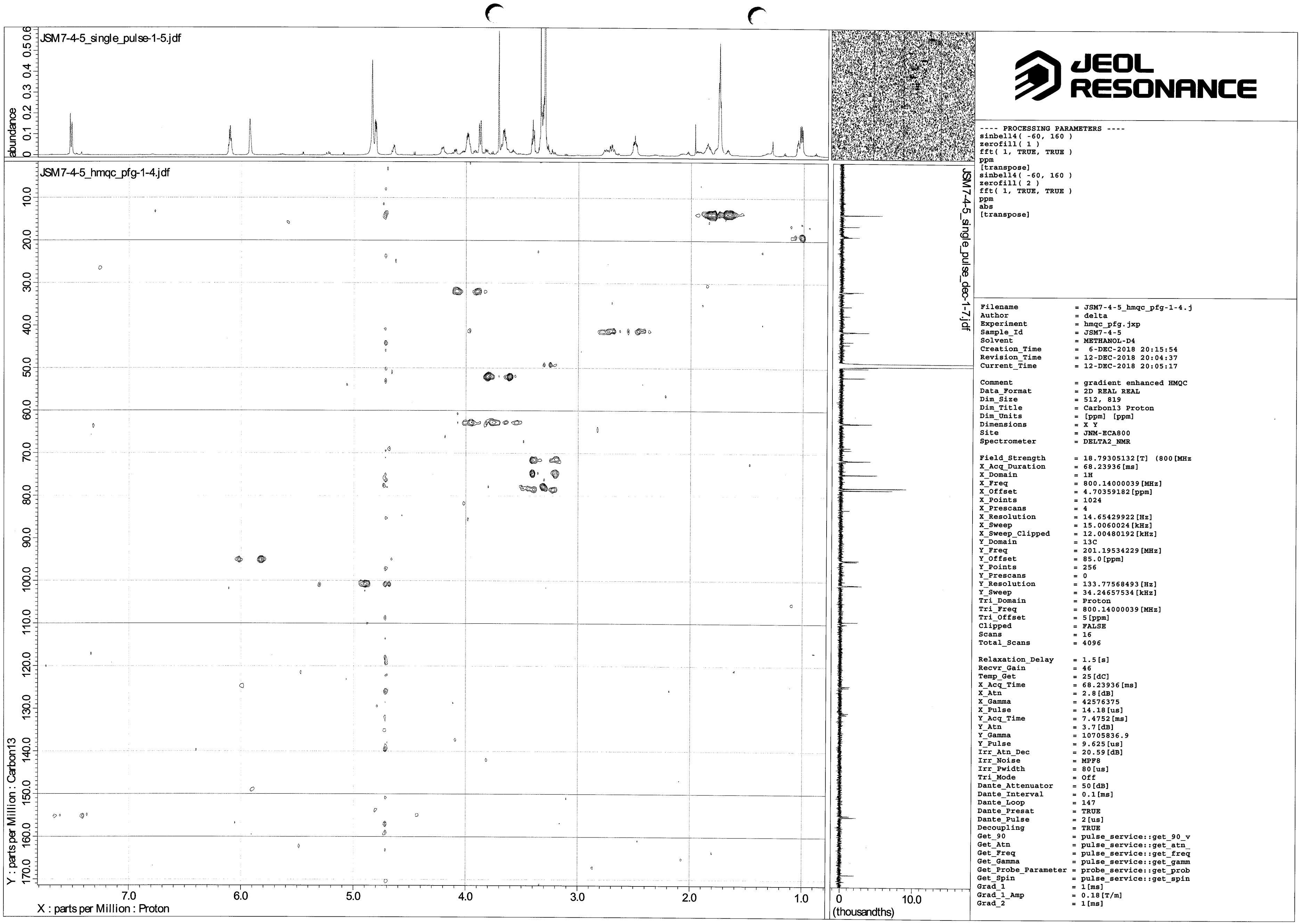

jasminumoside J (5)

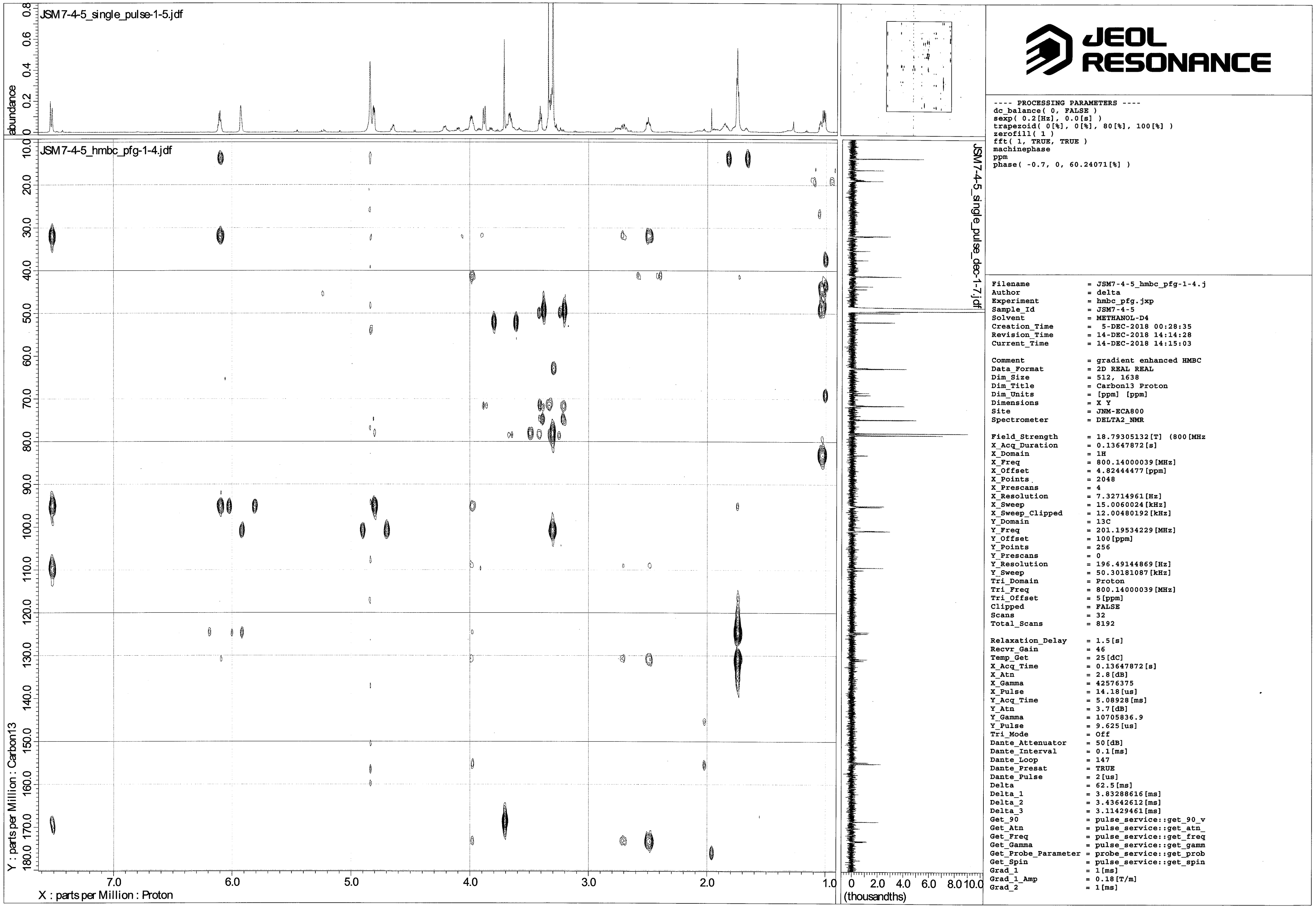

jasminumoside J (5)

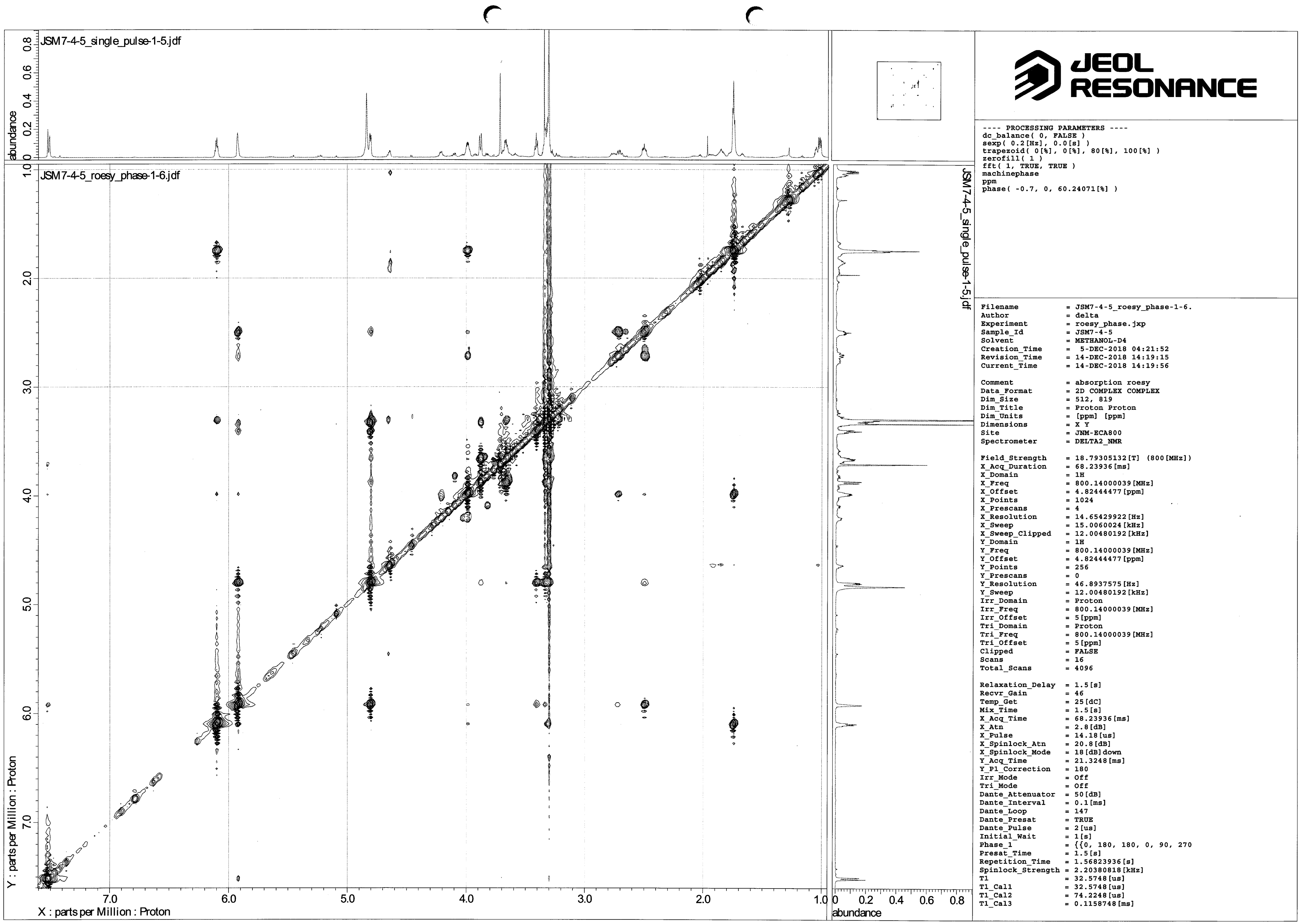

jasminumoside J (5)

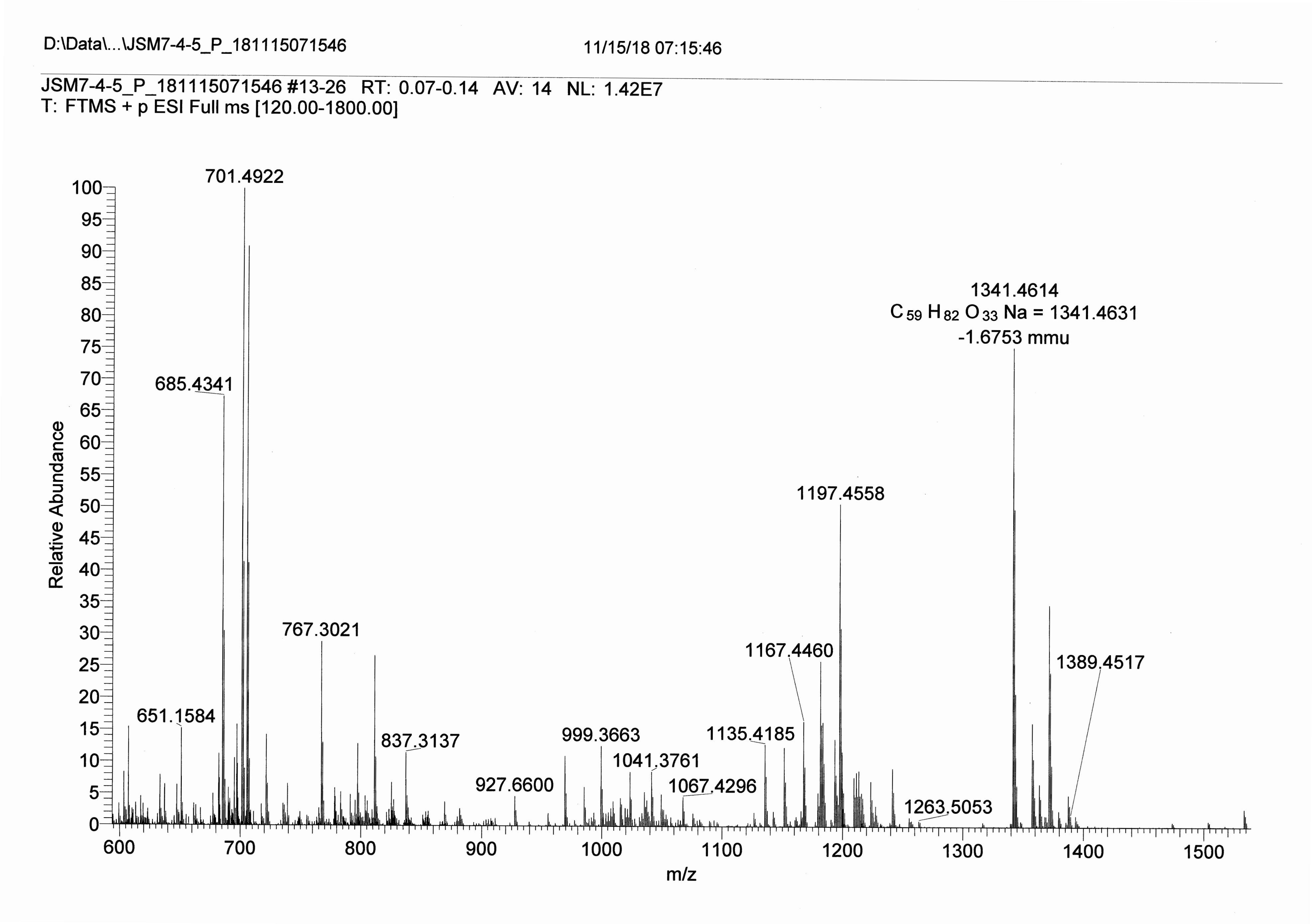

jasminumoside J (5)

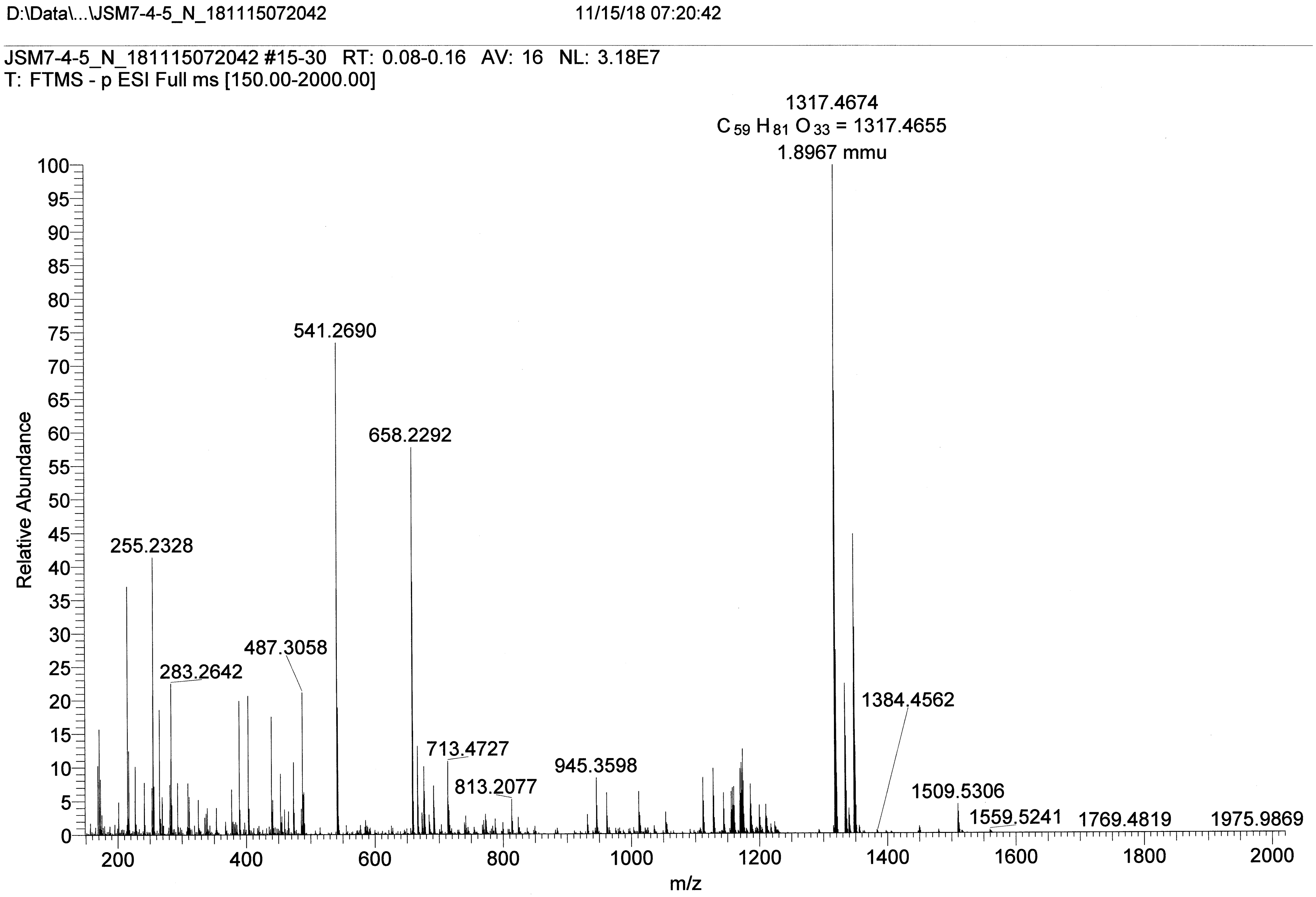

jasminumoside K (6)

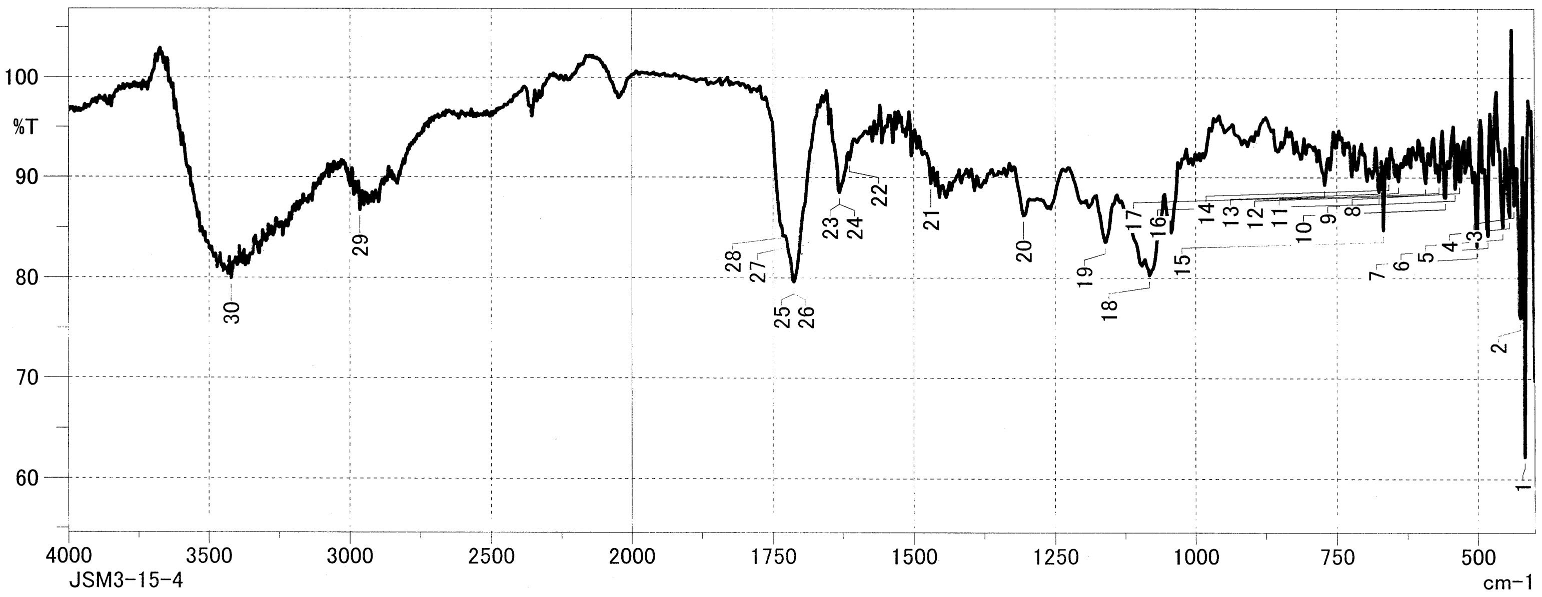

	ピーク
1	416.62
2	424.34
3	435.91
4	443.63
5	455.20
6	482.20
7	501.49
8	532.35
9	540.07
10	557.43
11	569.00
12	592.15
13	640.37
14	657.73
15	667.37
16	675.09
17	771.53
18	1082.07
19	1161.15
20	1305.81
21	1469.76
22	1614.42
23	1631.78
24	1631.78
25	1712.79
26	1712.79
27	1730.15
28	1735.93
29	2964.59
30	3421.72

	項目	値
	分析日時	2018/02/03 16:08:01
	分析者	System Administrator
	ファイル名	C:¥Users¥admin¥Desktop¥shokuhin01-312.ispd
	スペクトル名	shokuhin01-312
	サンプル名	
	サンプルID	
	オプション	
	コメント	JSM3-15-4
	積算回数	45
	分解	4 [cm-1]
	アポダイズ関数	Happ-Genzel

jasminumoside K (6)

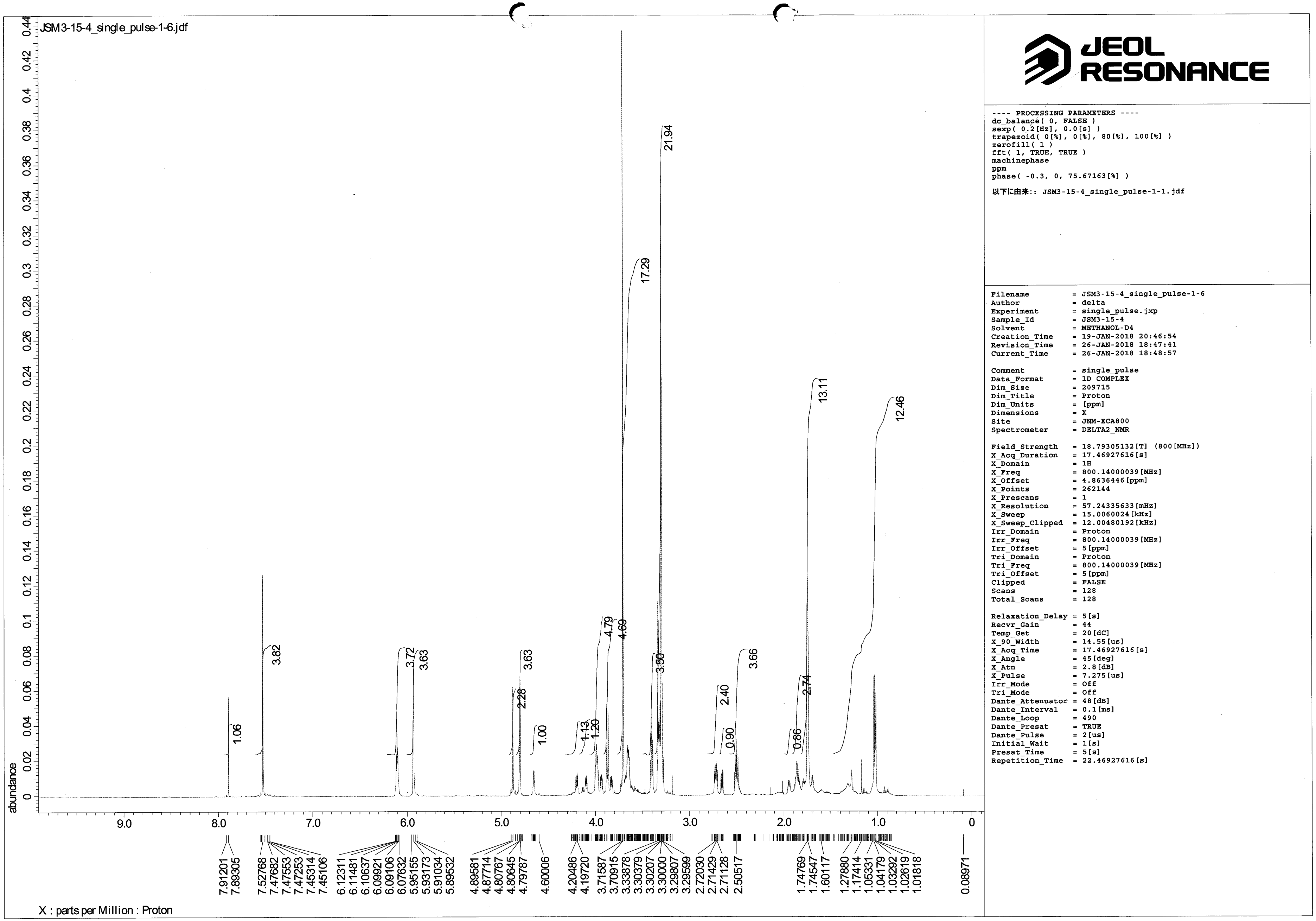

jasminumoside K (6)

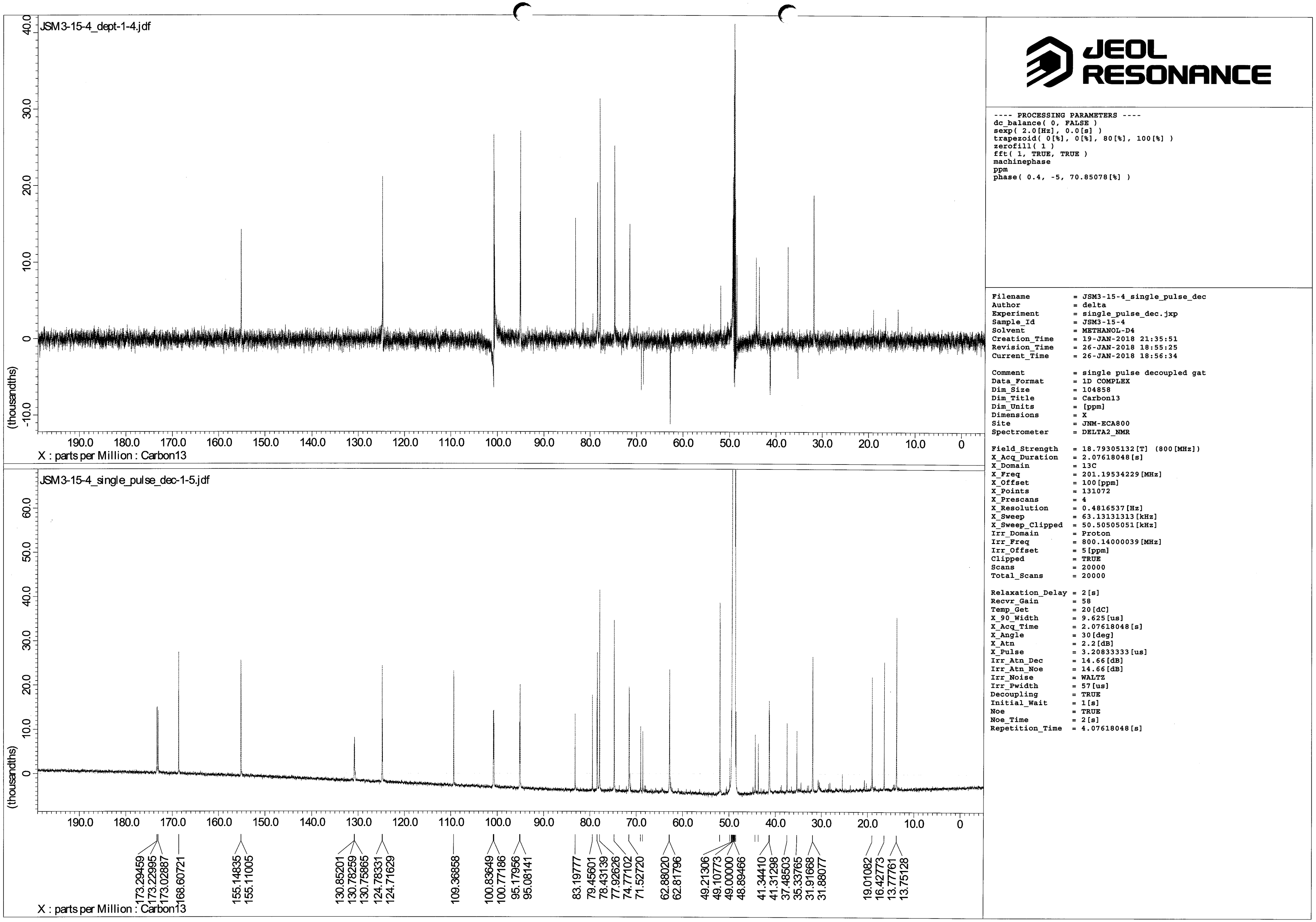

jasminumoside K (6)

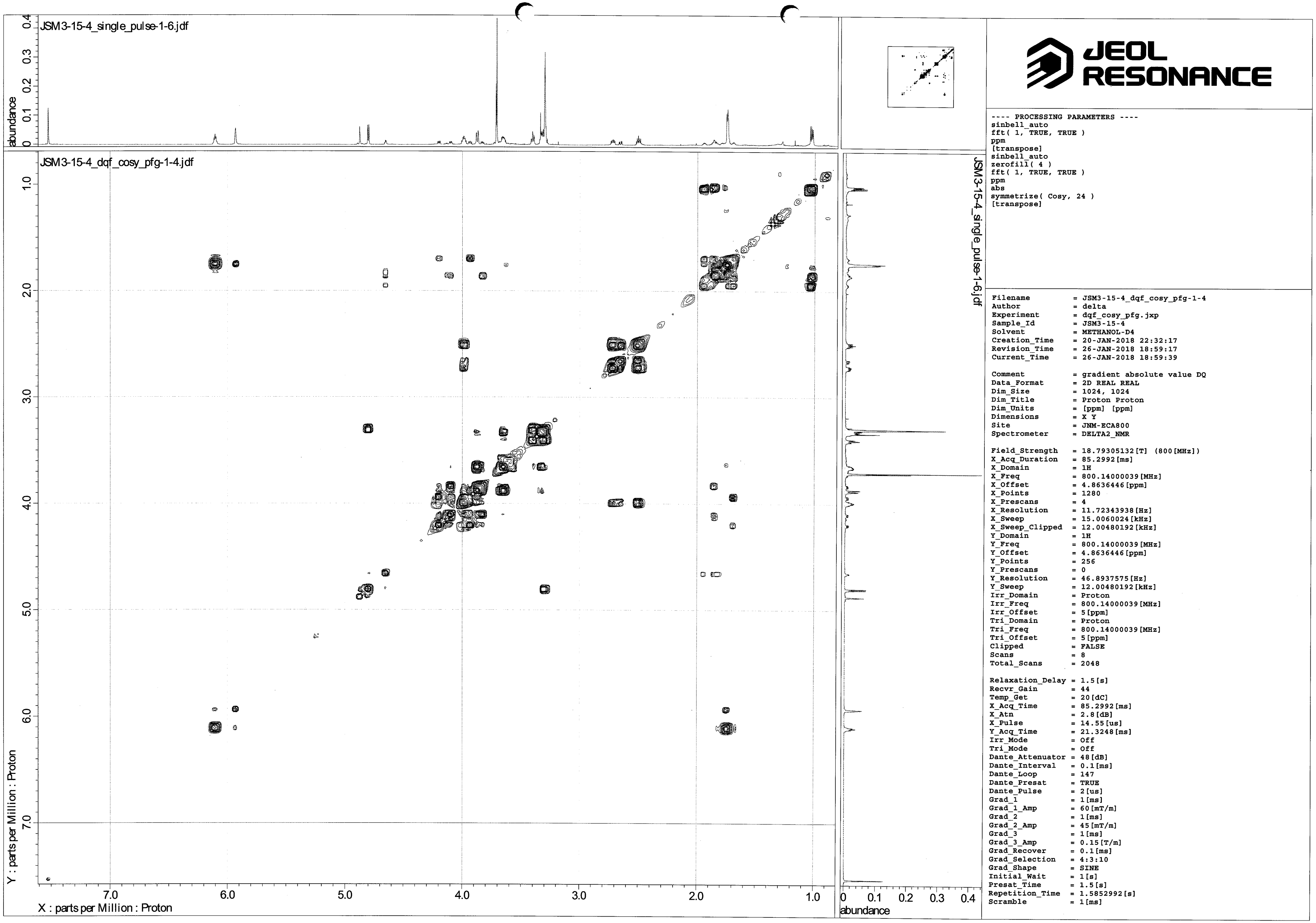

jasminumoside K (6)

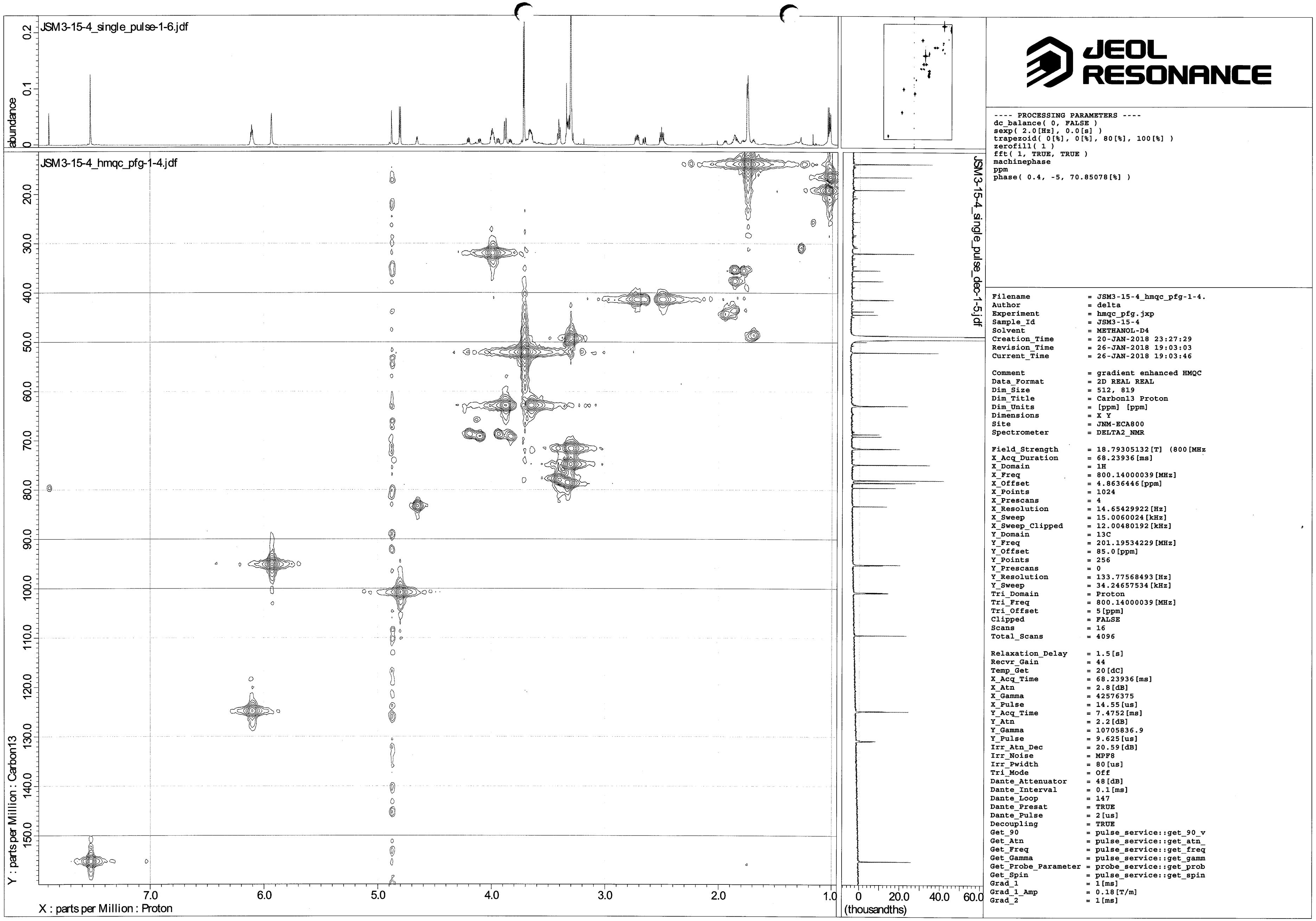

jasminumoside K (6)

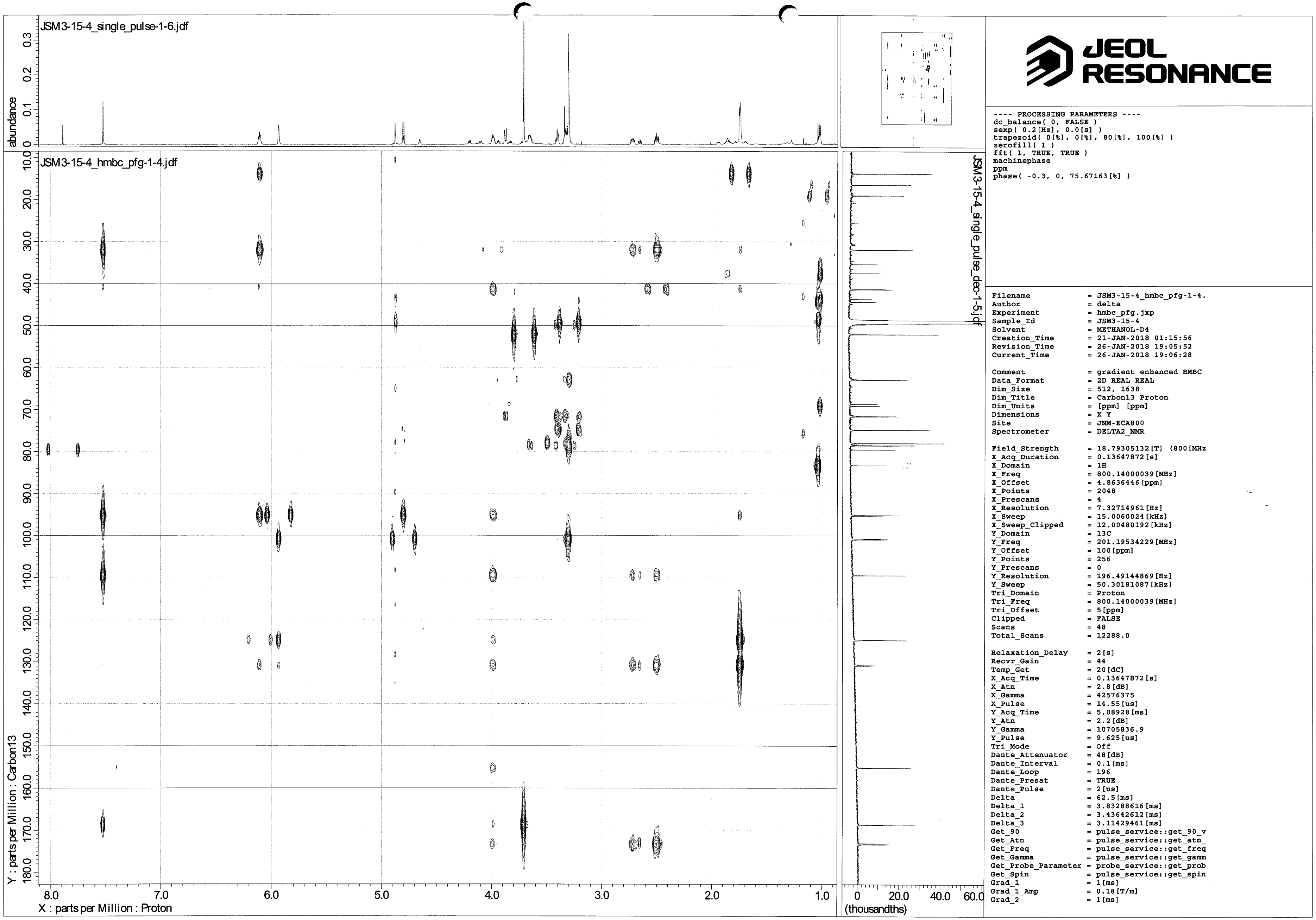

jasminumoside K (6)

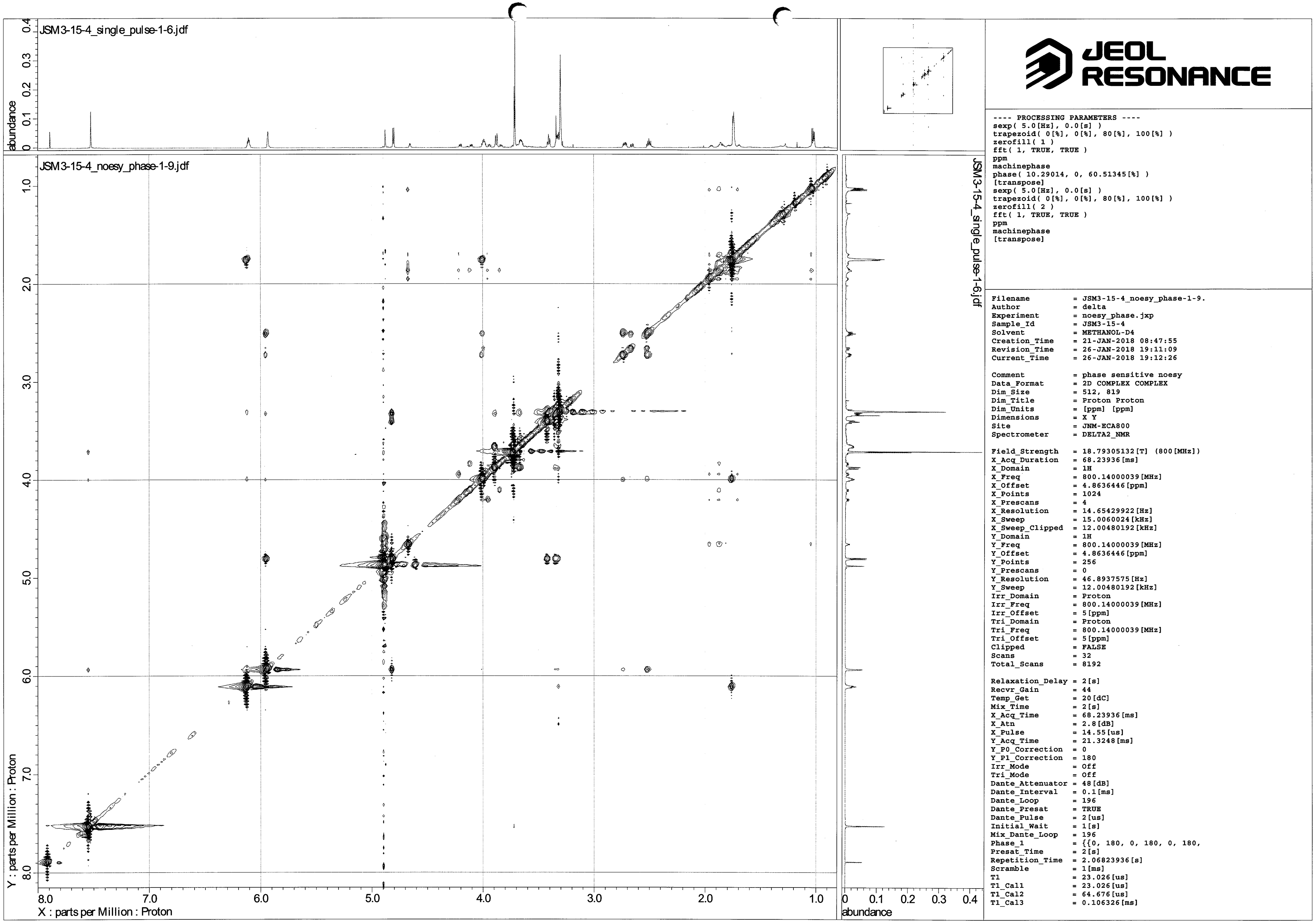

jasminumoside K (6)

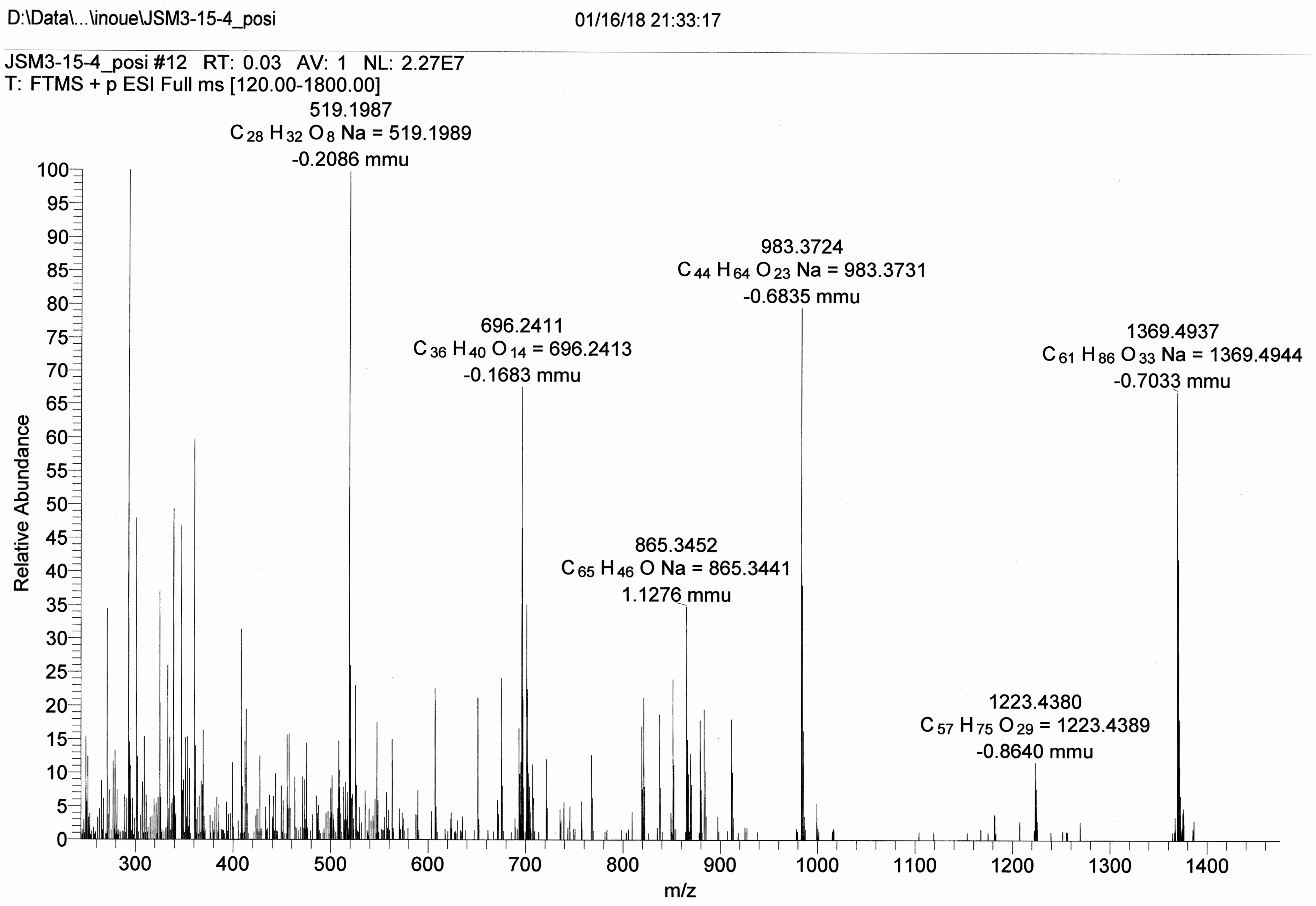

jasminumoside K (6)

D:\Data\...\JSM3-15-4_NEGA_180116213518 01/16/18 21:35:18

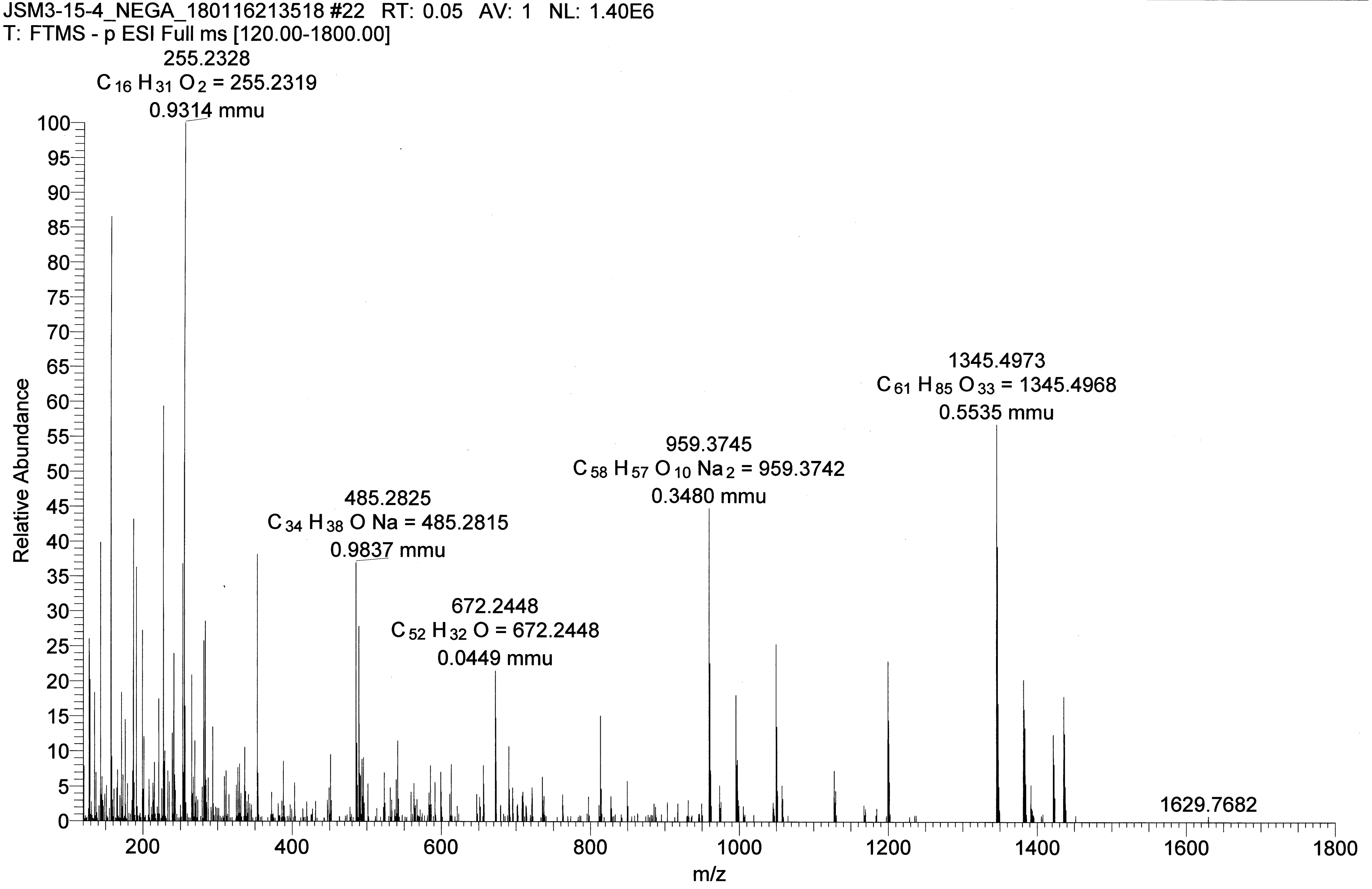

Supplement: Supplementary file 1 — Supplementary Material 1 [file 11418_2026_2018_MOESM1_ESM.pdf]
